# Supplementary material for: Quantitative Proteomic Approach Reveals Altered Metabolic Pathways in Response to the Inhibition of Lysine Deacetylases in A549 Cells under Normoxia and Hypoxia
Source: Int J Mol Sci. 2021 Mar 25;22(7):3378. doi: 10.3390/ijms22073378 (PMC8036653; doi:10.3390/ijms22073378)
Supplement: Supplementary file 1 [file ijms-22-03378-s001.zip › 20210323 Supplementary Files IJMS/Supplemental Data-revised 23 March 2021.docx]

## Supplemental Data

**Figure S1.** IC_50_ determination for trichostatin A (TSA) and nicotinamide (NAM). A549 cells were treated with different concentrations of trichostatin A (A) and nicotinamide (B). After 24 h of incubation cell viability was determined and represented as percentage of cells with respect to the control viability. Values are presented as mean ± standard deviation. ^24h^IC_50_ (TSA) = 3 μM; ^24h^IC_50_ (NAM) = 50 mM.

**Figure S2.** Cell cycle analysis of KDACI-treated cells under normoxia and hypoxia. A, B and C. A549 cells were treated with 1 μM of TSA, 20 mM of NAM and both 1 μM TSA and 20 mM NAM for 24 h of incubation under normoxia and hypoxia. Cells incubated in medium without KDACIs served as control. Bars represent the means ± standard error of the mean of three independent experiments. The asterisks above bars indicate statistically significant differences compared to normoxic control cells. The asterisks above curly brackets indicate statistically significant differences between hypoxic and normoxic treatments and between hypoxic treatments and hypoxic control cells. Statistical significance was assessed by a two-tailed Student’s t-test. *, p ≤ 0.05; **, p ≤ 0.01; ***, p ≤ 0.001.

**Figure S3.** Intracellular ROS levels of KDACI-treated A549 cells under normoxia and hypoxia. A549 cells were treated with 1 μM TSA, 20 mM NAM and both 1 μM TSA and 20 mM NAM under normoxia and hypoxia. Cells incubated in medium without KDACIs served as control. Bars represent the means ± standard error of the mean of three independent experiments. The asterisks above curly brackets indicate statistically significant differences between normoxic treatments and normoxic control cells and between hypoxic treatments and hypoxic control cells. Statistical significance was assessed by a two-tailed Student’s t-test. ***, p ≤ 0.001.; a. u., arbitrary units.

**Figure S4.** Representative FACS plots showing the gating strategy of the cell cycle analysis of KDACI-treated cells under normoxia and hypoxia. A549 cells were treated with 1 μM of TSA (b), 20 mM of NAM (c) and both 1 μM TSA and 20 mM NAM (d) for 24 h of incubation under normoxia. A549 cells were treated with 1 μM of TSA (f), 20 mM of NAM (g) and both 1 μM TSA and 20 mM NAM (h) for 24 h of incubation under hypoxia. Cells incubated in medium without KDACIs under normoxia (a) and hypoxia (e) served as control.

**Table S1.** Total list of quantified proteins. A549 cells were treated with 1 μM TSA, 20 mM NAM and both 1 μM TSA and 20 mM NAM under normoxia and hypoxia. Cells incubated in medium without KDACIs served as controls. The total list of quantified proteins is presented for TSA treatment under normoxia, NAM treatment under normoxia, TSA/NAM treatment under normoxia, control cells under hypoxia, TSA treatment under hypoxia, NAM treatment under hypoxia and TSA/NAM treatment under hypoxia with respect to control cells under normoxia. The UniProtKB accession number, number of peptides identified for each protein and name is presented with the ratio and p-value for each treatment. Values of the ratio and p-value of significantly (p-value ≤ 0.05) dysregulated proteins are marked in bold. Up-regulated proteins are colored in green (iTRAQ ratio > 1) and down-regulated proteins are colored in red (iTRAQ ratio < 1).

**Table S2.** Gene ontology enrichment analysis on biological process. A549 cells were treated with 1 μM TSA, 20 mM NAM and both 1 μM TSA and 20 mM NAM under normoxia and hypoxia. Cells incubated in medium without KDACIs served as controls. GO enrichment analysis of the Biological process is shown for TSA treatment under normoxia, NAM treatment under normoxia, TSA/NAM treatment under normoxia, control cells under hypoxia, TSA treatment under hypoxia, NAM treatment under hypoxia and TSA/NAM treatment under hypoxia with respect to control cells under normoxia. The list of the biological process is presented with the percentage of proteins and the p-value related to each process for each treatment. Significantly (p-value ≤ 0.05) enriched processes are colored in green if they belong to the list of up-regulated proteins or in red if they belong to the list of down-regulated proteins.

**Table S3.** Gene ontology enrichment analysis on PIR keywords. A549 cells were treated with 1 μM TSA, 20 mM NAM and both 1 μM TSA and 20 mM NAM under normoxia and hypoxia. Cells incubated in medium without KDACIs served as controls. GO enrichment analysis of the Protein Information Resource (PIR) keywords is shown for TSA treatment under normoxia, NAM treatment under normoxia, TSA/NAM treatment under normoxia, control cells under hypoxia, TSA treatment under hypoxia, NAM treatment under hypoxia and TSA/NAM treatment under hypoxia with respect to control cells under normoxia. The list of the PIR keyword is presented with the percentage of proteins and the p-value related to each keyword for each treatment. Significantly (p-value ≤ 0.05) enriched processes are colored in blue.

**Table S4.** List of the top 500 chemicals obtained from network analysis by node embeddings for each condition with drugs in clinical trials phase IV targeting each network of deregulated proteins upon TSA and NAM under normoxic and hypoxic treatments.

**Table S4-bis.** List of the top 70 chemicals obtained from network analysis by node embeddings with drugs in clinical trials phase IV targeting the network of deregulated proteins upon TSA and NAM under hypoxia. This list we used for creating the Figure 6 by excluding chemicals/metabolites

**Figure S1**

**
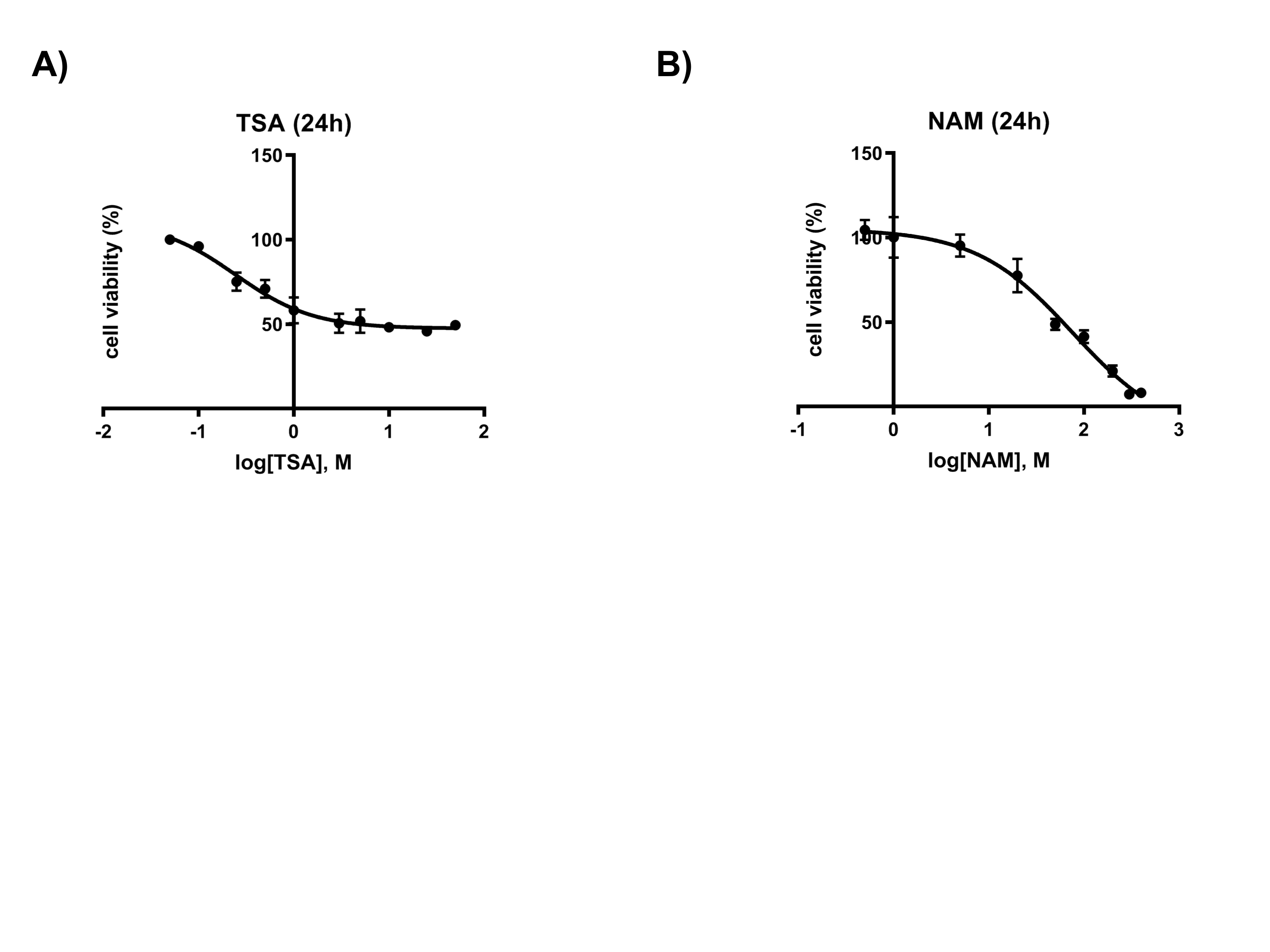
**

**Figure S2**

**Figure S3**

**Figure S4**
