# Supplementary material for: Quantitative Proteomic Approach Reveals Altered Metabolic Pathways in Response to the Inhibition of Lysine Deacetylases in A549 Cells under Normoxia and Hypoxia
Source: Int J Mol Sci. 2021 Mar 25;22(7):3378. doi: 10.3390/ijms22073378 (PMC8036653; doi:10.3390/ijms22073378)
Supplement: Supplementary file 1 [file ijms-22-03378-s001.zip › 20210323 Supplementary Files IJMS/Supplemental_Tables S1-S2-S3.docx]

**Table S1**

| **UniProtKB Accession Number** | **Protein Name** | **Peptides Number** | **TSA Normoxia** | | **NAM Normoxia** | | **TSA + NAM Normoxia** | | **Hypoxia** | | **TSA Hypoxia** | | **NAM Hypoxia** | | **TSA + NAM Hypoxia** | |
| --- | --- | --- | --- | --- | --- | --- | --- | --- | --- | --- | --- | --- | --- | --- | --- | --- |
|  |  |  | **ratio** | **p-value** | **ratio** | **p-value** | **ratio** | **p-value** | **ratio** | **p-value** | **ratio** | **p-value** | **ratio** | **p-value** | **ratio** | **p-value** |
| P63261 | Actin. cytoplasmic 2 | 63 | 0.92 | 0.398 | 0.88 | 0.356 | 0.83 | 0.244 | 0.91 | 0.350 | 0.90 | 0.382 | 0.76 | 0.225 | 0.80 | 0.234 |
| P05783 | Keratin. type I cytoskeletal 18 | 35 | 0.79 | 0.175 | 0.78 | 0.238 | 0.70 | 0.102 | 0.93 | 0.399 | 0.72 | 0.200 | 0.72 | 0.191 | 0.77 | 0.244 |
| P07900 | Heat shock protein HSP 90-alpha | 32 | 1.19 | 0.283 | 0.98 | 0.478 | 1.01 | 0.492 | 0.88 | 0.347 | 0.89 | 0.408 | 0.72 | 0.145 | 0.91 | 0.407 |
| P00352 | Retinal dehydrogenase 1 | 31 | 0.75 | 0.227 | 0.98 | 0.480 | 0.82 | 0.289 | 1.02 | 0.481 | 0.80 | 0.279 | 0.87 | 0.332 | 0.90 | 0.381 |
| P14618 | Pyruvate kinase PKM | 31 | 1.22 | 0.382 | 1.33 | 0.323 | 1.20 | 0.378 | 1.08 | 0.430 | 1.23 | 0.383 | 1.14 | 0.410 | 1.15 | 0.399 |
| Q15149 | Plectin | 31 | 1.00 | 0.495 | 1.01 | 0.495 | 0.93 | 0.415 | 1.07 | 0.427 | 1.05 | 0.449 | 0.99 | 0.485 | 1.01 | 0.489 |
| P04406 | Glyceraldehyde-3-phosphate dehydrogenase | 30 | 1.06 | 0.437 | 1.13 | 0.361 | 1.11 | 0.406 | 1.36 | 0.174 | 1.32 | 0.255 | 1.21 | 0.307 | 1.20 | 0.339 |
| P07355 | Annexin A2 | 29 | 0.74 | 0.267 | 0.87 | 0.363 | 0.75 | 0.268 | 0.89 | 0.354 | 0.73 | 0.277 | 0.84 | 0.347 | 0.78 | 0.284 |
| P05787 | Keratin. type II cytoskeletal 8 | 28 | 0.85 | 0.271 | 0.78 | 0.170 | 0.75 | 0.130 | 1.00 | 0.498 | 0.86 | 0.309 | 0.73 | 0.114 | 0.83 | 0.255 |
| O43707 | Alpha-actinin-4 | 26 | 0.93 | 0.417 | 0.96 | 0.434 | 0.86 | 0.347 | 0.97 | 0.463 | 0.89 | 0.379 | 0.80 | 0.275 | 1.02 | 0.483 |
| P21333 | Filamin-A | 26 | 0.91 | 0.397 | 0.89 | 0.367 | 0.89 | 0.360 | 0.93 | 0.402 | 0.85 | 0.324 | 0.78 | 0.223 | 0.93 | 0.413 |
| P11142 | Heat shock cognate 71 kDa protein | 25 | 0.69 | 0.147 | 0.91 | 0.390 | 0.75 | 0.175 | 0.94 | 0.421 | 0.73 | 0.230 | 0.72 | 0.141 | 0.79 | 0.258 |
| P07437 | Tubulin beta chain | 24 | 0.57 | 0.100 | 0.83 | 0.361 | 0.63 | 0.206 | 0.91 | 0.425 | 0.54 | 0.104 | 0.61 | 0.103 | 0.75 | 0.363 |
| Q5VTE0 | Putative elongation factor 1-alpha-like 3 | 24 | 0.94 | 0.407 | 1.11 | 0.313 | 0.85 | 0.211 | 1.12 | 0.305 | 0.96 | 0.438 | 0.92 | 0.324 | 1.05 | 0.399 |
| P62805 | Histone H4 | 23 | 0.93 | 0.411 | 0.95 | 0.438 | 0.97 | 0.454 | 0.97 | 0.462 | 0.98 | 0.478 | 0.94 | 0.436 | 1.07 | 0.419 |
| P68371 | Tubulin beta-4B chain | 23 | 0.76 | 0.362 | 0.75 | 0.264 | 0.86 | 0.398 | 0.82 | 0.332 | 0.67 | 0.210 | 0.88 | 0.389 | 0.98 | 0.482 |
| P06733 | Alpha-enolase | 22 | 1.39 | 0.175 | **1.78** | **0.032** | 1.50 | 0.093 | 1.41 | 0.156 | **1.71** | **0.025** | 1.66 | 0.068 | 1.59 | 0.070 |
| P08238 | Heat shock protein HSP 90-beta | 22 | 0.72 | 0.158 | 0.99 | 0.474 | 0.83 | 0.303 | 0.92 | 0.377 | 0.72 | 0.225 | 0.68 | 0.135 | 0.81 | 0.319 |
| Q9BVA1 | Tubulin beta-2B chain | 20 | **2.85** | **0.029** | 1.51 | 0.264 | **2.17** | **0.030** | 0.72 | 0.300 | **2.66** | **0.032** | 1.55 | 0.290 | 1.84 | 0.066 |
| P22626 | Heterogeneous nuclear ribonucleoproteins A2/B1 | 19 | 0.82 | 0.347 | 1.06 | 0.456 | 0.79 | 0.312 | 0.85 | 0.335 | 0.81 | 0.345 | 0.87 | 0.387 | 0.82 | 0.309 |
| Q5QNW6 | Histone H2B type 2-F | 19 | **0.48** | **0.037** | 0.78 | 0.251 | 0.63 | 0.112 | 0.79 | 0.266 | 0.71 | 0.186 | 0.65 | 0.135 | 0.59 | 0.091 |
| P08729 | Keratin. type II cytoskeletal 7 | 18 | 0.73 | 0.193 | 0.78 | 0.223 | 0.65 | 0.086 | 0.98 | 0.476 | 0.77 | 0.232 | 0.70 | 0.102 | 0.78 | 0.227 |
| P10809 | 60 kDa heat shock protein. mitochondrial | 18 | 0.95 | 0.441 | 0.99 | 0.478 | 0.96 | 0.452 | 0.94 | 0.419 | 0.94 | 0.432 | 0.91 | 0.393 | 1.01 | 0.498 |
| P11413 | Glucose-6-phosphate 1-dehydrogenase | 18 | 0.82 | 0.234 | 1.09 | 0.396 | 0.86 | 0.264 | 0.96 | 0.440 | 0.86 | 0.341 | 0.91 | 0.395 | 0.95 | 0.436 |
| Q13509 | Tubulin beta-3 chain | 18 | 1.35 | 0.080 | 1.05 | 0.427 | 1.19 | 0.209 | 1.21 | 0.171 | 1.28 | 0.172 | 0.92 | 0.293 | 1.25 | 0.114 |
| O60218 | Aldo-keto reductase family 1 member B10 | 17 | 0.80 | 0.247 | 1.12 | 0.333 | 0.93 | 0.390 | 1.15 | 0.313 | 0.88 | 0.314 | 1.04 | 0.435 | 1.03 | 0.457 |
| P08107 | Heat shock 70 kDa protein 1A/1B | 17 | 0.97 | 0.469 | 0.89 | 0.350 | 0.85 | 0.344 | 0.67 | 0.093 | 0.83 | 0.318 | 0.69 | 0.154 | 0.74 | 0.219 |
| P09651 | Heterogeneous nuclear ribonucleoprotein A1 | 17 | 0.98 | 0.479 | 1.05 | 0.457 | 0.88 | 0.382 | 0.93 | 0.401 | 0.90 | 0.420 | 0.78 | 0.301 | 0.90 | 0.396 |
| P13639 | Elongation factor 2 | 17 | 0.86 | 0.320 | 0.94 | 0.398 | 0.89 | 0.351 | 0.96 | 0.439 | 0.88 | 0.316 | 0.76 | 0.187 | 0.91 | 0.386 |
| P42330 | Aldo-keto reductase family 1 member C3 | 17 | 1.19 | 0.268 | 1.81 | 0.055 | 1.39 | 0.199 | 1.24 | 0.193 | 1.31 | 0.259 | 1.36 | 0.274 | 1.44 | 0.168 |
| Q9BQE3 | Tubulin alpha-1C chain | 17 | 0.90 | 0.370 | 0.87 | 0.319 | 0.86 | 0.300 | 1.02 | 0.478 | 0.90 | 0.380 | 0.83 | 0.252 | 0.93 | 0.402 |
| P06899 | Histone H2B type 1-J | 16 | 0.65 | 0.195 | 0.71 | 0.241 | 0.58 | 0.139 | 0.53 | 0.109 | 0.57 | 0.140 | 0.91 | 0.418 | 0.76 | 0.293 |
| P12814 | Alpha-actinin-1 | 16 | 0.98 | 0.458 | 0.81 | 0.285 | 0.79 | 0.168 | 1.11 | 0.311 | 0.97 | 0.449 | 0.88 | 0.155 | 0.74 | 0.192 |
| P35579 | Myosin-9 | 16 | 1.04 | 0.471 | 1.03 | 0.471 | 1.26 | 0.298 | 1.14 | 0.360 | 1.07 | 0.454 | 1.21 | 0.359 | 1.00 | 0.498 |
| P49327 | Fatty acid synthase | 16 | 1.26 | 0.313 | 1.05 | 0.460 | 0.95 | 0.399 | 1.14 | 0.274 | 1.22 | 0.316 | 1.05 | 0.459 | 1.01 | 0.493 |
| Q00610 | Clathrin heavy chain 1 | 16 | 1.30 | 0.235 | 1.35 | 0.135 | 1.42 | 0.189 | 1.17 | 0.283 | 1.47 | 0.135 | 1.38 | 0.204 | 1.23 | 0.240 |
| Q05639 | Elongation factor 1-alpha 2 | 15 | 0.98 | 0.474 | **1.52** | **0.036** | 0.86 | 0.376 | 1.32 | 0.294 | 0.87 | 0.298 | 1.34 | 0.319 | 1.09 | 0.341 |
| Q16881 | Thioredoxin reductase 1. cytoplasmic | 15 | 1.20 | 0.307 | 1.40 | 0.157 | 1.17 | 0.329 | 1.39 | 0.149 | 1.14 | 0.365 | 1.31 | 0.230 | 1.21 | 0.305 |
| O75369 | Filamin-B | 14 | 0.89 | 0.355 | 0.75 | 0.165 | 0.88 | 0.317 | 0.94 | 0.415 | 0.87 | 0.329 | 0.79 | 0.207 | 0.91 | 0.363 |
| P00338 | L-lactate dehydrogenase A chain | 14 | 0.89 | 0.382 | 1.10 | 0.396 | 0.93 | 0.401 | 1.71 | 0.071 | 1.31 | 0.275 | 1.19 | 0.326 | 1.17 | 0.341 |
| P04083 | Annexin A1 | 14 | 1.01 | 0.499 | 0.93 | 0.412 | 0.88 | 0.385 | 0.96 | 0.451 | 0.93 | 0.435 | 0.93 | 0.430 | 0.89 | 0.370 |
| P06748 | Nucleophosmin | 14 | 0.90 | 0.378 | 0.89 | 0.347 | 0.88 | 0.365 | 1.02 | 0.474 | 0.94 | 0.421 | 0.91 | 0.386 | 0.97 | 0.451 |
| P52895 | Aldo-keto reductase family 1 member C2 | 14 | 0.99 | 0.483 | 1.57 | 0.134 | 1.28 | 0.287 | 1.52 | 0.190 | 1.22 | 0.325 | 1.32 | 0.267 | 1.29 | 0.262 |
| P06576 | ATP synthase subunit beta. mitochondrial | 13 | 1.24 | 0.320 | 1.20 | 0.318 | 1.31 | 0.279 | 1.08 | 0.398 | 1.23 | 0.310 | 1.11 | 0.409 | 1.29 | 0.234 |
| P15559 | NAD(P)H dehydrogenase [quinone] 1 | 13 | 1.00 | 0.497 | 1.39 | 0.238 | 1.20 | 0.327 | 0.96 | 0.445 | 0.95 | 0.459 | 1.03 | 0.485 | 1.23 | 0.308 |
| P29401 | Transketolase | 13 | 0.95 | 0.440 | 1.04 | 0.469 | 0.93 | 0.418 | 0.99 | 0.486 | 0.99 | 0.488 | 0.89 | 0.406 | 1.07 | 0.442 |
| P68366 | Tubulin alpha-4A chain | 13 | 2.33 | 0.118 | 0.98 | 0.479 | 2.62 | 0.146 | 0.85 | 0.387 | 0.98 | 0.481 | 2.17 | 0.181 | 1.02 | 0.484 |
| P78371 | T-complex protein 1 subunit beta | 13 | 1.31 | 0.272 | 1.51 | 0.175 | 1.30 | 0.285 | 1.10 | 0.384 | 1.44 | 0.182 | 1.22 | 0.305 | 1.28 | 0.249 |
| P06744 | Glucose-6-phosphate isomerase | 12 | 0.77 | 0.186 | 0.95 | 0.392 | 0.69 | 0.155 | **1.74** | **0.022** | 1.23 | 0.128 | 0.89 | 0.348 | 1.04 | 0.431 |
| P08670 | Vimentin | 12 | 0.72 | 0.089 | 0.82 | 0.224 | 0.67 | 0.118 | 0.94 | 0.404 | 0.77 | 0.179 | 0.79 | 0.229 | 0.73 | 0.175 |
| P23246 | Splicing factor. proline- and glutamine-rich | 12 | 1.03 | 0.469 | 0.97 | 0.456 | 0.90 | 0.392 | 1.00 | 0.496 | 0.80 | 0.306 | 0.88 | 0.353 | 0.87 | 0.364 |
| P07737 | Profilin-1 | 11 | 0.83 | 0.275 | 0.92 | 0.391 | 0.95 | 0.438 | 0.99 | 0.485 | 0.77 | 0.254 | 0.81 | 0.243 | 1.06 | 0.435 |
| P62937 | Peptidyl-prolyl cis-trans isomerase A | 11 | 1.59 | 0.295 | 0.94 | 0.427 | 1.27 | 0.383 | 1.04 | 0.469 | 1.09 | 0.429 | 1.59 | 0.308 | 1.19 | 0.339 |
| P15121 | Aldose reductase | 10 | 1.35 | 0.209 | 1.27 | 0.156 | 1.28 | 0.243 | 1.24 | 0.240 | 1.35 | 0.154 | 1.21 | 0.277 | 1.27 | 0.222 |
| P26641 | Elongation factor 1-gamma | 10 | 0.86 | 0.312 | 0.92 | 0.359 | 0.94 | 0.402 | 0.94 | 0.407 | 0.88 | 0.292 | 0.90 | 0.391 | 0.95 | 0.399 |
| P30838 | Aldehyde dehydrogenase. dimeric NADP-preferring | 10 | **0.65** | **0.048** | 0.76 | 0.202 | 0.73 | 0.142 | 0.86 | 0.279 | 0.64 | 0.132 | 0.74 | 0.141 | 0.81 | 0.270 |
| P31943 | Heterogeneous nuclear ribonucleoprotein H | 10 | **0.75** | **0.016** | 0.78 | 0.199 | **0.58** | **0.040** | 0.82 | 0.089 | **0.41** | **0.000** | **0.63** | **0.007** | **0.63** | **0.005** |
| P38646 | Stress-70 protein. mitochondrial | 10 | 0.94 | 0.409 | 0.97 | 0.456 | 0.96 | 0.444 | 0.88 | 0.282 | 0.97 | 0.446 | 0.86 | 0.294 | 0.98 | 0.466 |
| P60174 | Triosephosphate isomerase | 10 | 1.11 | 0.365 | 1.04 | 0.447 | 1.09 | 0.368 | 1.26 | 0.226 | 1.27 | 0.249 | 1.12 | 0.331 | 1.24 | 0.237 |
| Q14204 | Cytoplasmic dynein 1 heavy chain 1 | 10 | 1.16 | 0.365 | 1.24 | 0.304 | 1.34 | 0.241 | 1.17 | 0.369 | 1.13 | 0.360 | 1.47 | 0.117 | 1.26 | 0.254 |
| P00558 | Phosphoglycerate kinase 1 | 9 | 2.04 | 0.107 | 1.32 | 0.220 | 2.34 | 0.138 | 1.80 | 0.125 | 1.65 | 0.190 | 2.89 | 0.087 | 1.57 | 0.143 |
| P04075 | Fructose-bisphosphate aldolase A | 9 | 0.97 | 0.458 | 0.93 | 0.418 | 0.86 | 0.325 | 1.30 | 0.141 | 1.32 | 0.174 | 1.00 | 0.492 | 1.25 | 0.197 |
| P11021 | 78 kDa glucose-regulated protein | 9 | 1.08 | 0.403 | 0.97 | 0.452 | 1.04 | 0.454 | 0.92 | 0.375 | 1.13 | 0.322 | 0.77 | 0.253 | 1.17 | 0.321 |
| P04264 | Keratin. type II cytoskeletal 1 | 8 | 0.77 | 0.286 | **0.65** | **0.005** | 0.65 | 0.296 | 1.18 | 0.273 | 0.98 | 0.481 | 0.66 | 0.311 | 0.69 | 0.247 |
| P07237 | Protein disulfide-isomerase | 8 | 1.34 | 0.258 | 1.17 | 0.340 | 1.35 | 0.282 | 1.07 | 0.442 | 1.46 | 0.246 | 1.15 | 0.383 | 1.25 | 0.317 |
| P53396 | ATP-citrate synthase | 8 | 1.09 | 0.403 | 1.07 | 0.438 | 1.09 | 0.396 | 0.99 | 0.488 | 1.02 | 0.480 | 1.06 | 0.448 | 1.12 | 0.383 |
| P78527 | DNA-dependent protein kinase catalytic subunit | 8 | 1.26 | 0.301 | 1.33 | 0.195 | 1.16 | 0.342 | 1.31 | 0.279 | 1.45 | 0.186 | 1.32 | 0.184 | 1.16 | 0.351 |
| O60701 | UDP-glucose 6-dehydrogenase | 7 | 0.98 | 0.478 | 1.44 | 0.209 | 1.17 | 0.372 | 1.38 | 0.190 | 1.06 | 0.460 | 1.28 | 0.315 | 1.25 | 0.309 |
| P01023 | Alpha-2-macroglobulin | 7 | 1.76 | 0.146 | 1.50 | 0.167 | 2.12 | 0.056 | 1.01 | 0.490 | **2.44** | **0.038** | 1.92 | 0.072 | 1.58 | 0.163 |
| P04843 | Dolichyl-diphosphooligosaccharide--protein glycosyltransferase subunit 1 | 7 | 0.86 | 0.349 | 1.08 | 0.416 | 0.93 | 0.423 | 1.16 | 0.228 | 1.03 | 0.471 | 1.13 | 0.375 | 1.06 | 0.444 |
| P05141 | ADP/ATP translocase 2 | 7 | 1.07 | 0.353 | 1.12 | 0.265 | 0.97 | 0.428 | 1.01 | 0.480 | 1.27 | 0.226 | 1.08 | 0.381 | 1.01 | 0.487 |
| P09211 | Glutathione S-transferase P | 7 | 1.05 | 0.430 | 0.87 | 0.311 | 1.09 | 0.345 | 1.10 | 0.375 | 1.14 | 0.340 | 1.07 | 0.405 | 1.09 | 0.350 |
| P15311 | Ezrin | 7 | 0.81 | 0.194 | 0.82 | 0.251 | 0.87 | 0.261 | 0.95 | 0.399 | 0.71 | 0.126 | 0.73 | 0.168 | 0.76 | 0.194 |
| P18206 | Vinculin | 7 | 1.27 | 0.332 | 0.97 | 0.468 | 1.36 | 0.220 | 0.96 | 0.464 | 1.37 | 0.226 | 1.05 | 0.455 | 1.19 | 0.374 |
| P42704 | Leucine-rich PPR motif-containing protein. mitochondrial | 7 | 1.34 | 0.198 | 1.08 | 0.412 | 1.27 | 0.262 | 0.92 | 0.302 | 1.39 | 0.205 | 1.07 | 0.404 | 1.14 | 0.369 |
| P52209 | 6-phosphogluconate dehydrogenase. decarboxylating | 7 | 0.70 | 0.099 | 1.03 | 0.462 | 0.79 | 0.149 | 0.91 | 0.350 | 0.82 | 0.287 | 0.80 | 0.235 | 0.85 | 0.310 |
| P55072 | Transitional endoplasmic reticulum ATPase | 7 | 0.79 | 0.137 | 0.96 | 0.441 | 1.02 | 0.474 | 0.91 | 0.374 | 0.90 | 0.399 | 0.88 | 0.311 | 1.07 | 0.410 |
| P61981 | 14-3-3 protein gamma | 7 | 0.90 | 0.153 | 0.90 | 0.168 | 0.82 | 0.186 | **0.81** | **0.024** | **0.80** | **0.026** | 0.91 | 0.179 | 0.96 | 0.332 |
| P63104 | 14-3-3 protein zeta/delta | 7 | 0.63 | 0.075 | 0.85 | 0.211 | 0.66 | 0.121 | 0.69 | 0.156 | 0.63 | 0.072 | 0.64 | 0.053 | 0.69 | 0.087 |
| P69905 | Hemoglobin subunit alpha | 7 | 1.25 | 0.246 | 0.99 | 0.480 | 1.21 | 0.247 | 1.22 | 0.172 | 1.22 | 0.337 | 1.19 | 0.298 | 1.38 | 0.159 |
| Q01813 | ATP-dependent 6-phosphofructokinase. platelet type | 7 | 1.01 | 0.492 | 1.15 | 0.381 | 1.25 | 0.249 | 1.30 | 0.202 | 1.14 | 0.403 | 1.32 | 0.293 | 1.26 | 0.190 |
| Q08211 | ATP-dependent RNA helicase A | 7 | 1.00 | 0.499 | 1.01 | 0.491 | 1.07 | 0.446 | 1.04 | 0.449 | 1.01 | 0.494 | 0.99 | 0.484 | 0.94 | 0.437 |
| P02545 | Prelamin-A/C | 6 | 0.84 | 0.339 | 0.85 | 0.264 | 0.83 | 0.313 | 0.82 | 0.333 | 1.02 | 0.486 | 0.63 | 0.156 | 0.72 | 0.209 |
| P08727 | Keratin. type I cytoskeletal 19 | 6 | 1.02 | 0.489 | 0.77 | 0.254 | **0.77** | **0.018** | 0.79 | 0.256 | 0.84 | 0.341 | 0.78 | 0.218 | 0.79 | 0.075 |
| P11586 | C-1-tetrahydrofolate synthase. cytoplasmic | 6 | 0.78 | 0.142 | **0.79** | **0.039** | 0.94 | 0.350 | 0.92 | 0.275 | 0.75 | 0.172 | 0.83 | 0.251 | 0.80 | 0.126 |
| P12235 | ADP/ATP translocase 1 | 6 | **2.68** | **0.034** | **2.92** | **0.022** | **2.46** | **0.046** | 0.92 | 0.444 | **4.68** | **0.002** | 1.58 | 0.210 | **3.27** | **0.014** |
| P18669 | Phosphoglycerate mutase 1 | 6 | 0.82 | 0.277 | 1.04 | 0.449 | 0.89 | 0.182 | 1.40 | 0.186 | 0.90 | 0.427 | 0.93 | 0.431 | 1.05 | 0.464 |
| P27348 | 14-3-3 protein theta | 6 | 0.81 | 0.293 | 0.88 | 0.339 | 0.68 | 0.166 | 0.72 | 0.140 | 0.72 | 0.192 | 0.80 | 0.243 | 0.70 | 0.112 |
| P31946 | 14-3-3 protein beta/alpha | 6 | 0.97 | 0.462 | 0.81 | 0.186 | 0.87 | 0.268 | 0.96 | 0.456 | 0.56 | 0.056 | **0.67** | **0.019** | 0.83 | 0.206 |
| P40926 | Malate dehydrogenase. mitochondrial | 6 | 1.54 | 0.121 | 1.28 | 0.194 | 1.36 | 0.119 | 1.11 | 0.359 | 1.48 | 0.095 | 1.10 | 0.366 | 1.48 | 0.113 |
| P52597 | Heterogeneous nuclear ribonucleoprotein F | 6 | 0.78 | 0.305 | 1.32 | 0.275 | 0.59 | 0.140 | 0.98 | 0.482 | 0.48 | 0.081 | 0.77 | 0.298 | 0.44 | 0.063 |
| P61978 | Heterogeneous nuclear ribonucleoprotein K | 6 | 0.73 | 0.289 | 0.77 | 0.246 | 0.69 | 0.253 | 0.79 | 0.252 | 0.63 | 0.208 | 0.64 | 0.177 | 0.68 | 0.233 |
| P62241 | 40S ribosomal protein S8 | 6 | 0.73 | 0.148 | 0.88 | 0.295 | **0.65** | **0.022** | 0.88 | 0.318 | 0.74 | 0.170 | 0.72 | 0.130 | 0.79 | 0.171 |
| P68871 | Hemoglobin subunit beta | 6 | **1.85** | **0.000** | **1.56** | **0.000** | **2.03** | **0.000** | **1.40** | **0.001** | **2.30** | **0.000** | **1.94** | **0.000** | **1.83** | **0.000** |
| Q00325 | Phosphate carrier protein. mitochondrial | 6 | **0.65** | **0.002** | 0.80 | 0.305 | 0.84 | 0.075 | 0.76 | 0.102 | 1.01 | 0.497 | 0.72 | 0.122 | 0.95 | 0.426 |
| Q04917 | 14-3-3 protein eta | 6 | 0.66 | 0.166 | 0.65 | 0.156 | **0.45** | **0.033** | 0.62 | 0.135 | 0.49 | 0.056 | 0.52 | 0.067 | 0.56 | 0.094 |
| Q13813 | Spectrin alpha chain. non-erythrocytic 1 | 6 | 0.97 | 0.457 | 1.03 | 0.466 | 1.00 | 0.499 | 1.09 | 0.395 | 0.84 | 0.331 | 1.10 | 0.407 | 0.88 | 0.356 |
| Q14697 | Neutral alpha-glucosidase AB | 6 | 0.97 | 0.457 | 1.10 | 0.300 | 1.01 | 0.492 | 0.90 | 0.403 | 0.84 | 0.283 | 1.08 | 0.394 | 1.10 | 0.412 |
| Q14974 | Importin subunit beta-1 | 6 | 0.79 | 0.323 | 1.02 | 0.491 | 0.89 | 0.385 | 1.09 | 0.412 | 0.89 | 0.402 | 0.95 | 0.456 | 0.92 | 0.429 |
| Q15233 | Non-POU domain-containing octamer-binding protein | 6 | 0.74 | 0.148 | 0.77 | 0.236 | 0.70 | 0.149 | 0.84 | 0.248 | 0.69 | 0.149 | 0.67 | 0.135 | 0.83 | 0.251 |
| O00571 | ATP-dependent RNA helicase DDX3X | 5 | 0.79 | 0.222 | 0.77 | 0.186 | 0.73 | 0.200 | 0.93 | 0.419 | 0.78 | 0.220 | 0.79 | 0.199 | 0.78 | 0.217 |
| P02788 | Lactotransferrin | 5 | **1.39** | **0.020** | 1.20 | 0.189 | **1.48** | **0.034** | 1.07 | 0.397 | **1.56** | **0.018** | **1.40** | **0.036** | **1.33** | **0.046** |
| P04792 | Heat shock protein beta-1 | 5 | 0.70 | 0.202 | 0.88 | 0.335 | 0.80 | 0.254 | 1.01 | 0.493 | 0.86 | 0.350 | 1.00 | 0.495 | 0.70 | 0.184 |
| P11216 | Glycogen phosphorylase. brain form | 5 | 1.14 | 0.398 | 1.13 | 0.407 | 0.95 | 0.418 | 1.06 | 0.446 | 1.19 | 0.354 | 1.26 | 0.181 | 1.14 | 0.324 |
| P11940 | Polyadenylate-binding protein 1 | 5 | 0.78 | 0.160 | **0.73** | **0.003** | 0.73 | 0.099 | 0.84 | 0.188 | 0.83 | 0.253 | **0.64** | **0.009** | **0.74** | **0.020** |
| P14174 | Macrophage migration inhibitory factor | 5 | 2.27 | 0.204 | 1.08 | 0.395 | 3.35 | 0.186 | 1.22 | 0.356 | 0.92 | 0.414 | 3.35 | 0.150 | 1.17 | 0.177 |
| P14625 | Endoplasmin | 5 | 1.08 | 0.426 | 0.97 | 0.465 | 0.88 | 0.325 | 1.00 | 0.496 | 1.10 | 0.403 | 0.91 | 0.372 | 1.01 | 0.489 |
| P15531 | Nucleoside diphosphate kinase A | 5 | **2.67** | **0.001** | **3.07** | **0.000** | **2.47** | **0.002** | **2.31** | **0.005** | **4.22** | **0.000** | **4.62** | **0.000** | **2.14** | **0.010** |
| P16403 | Histone H1.2 | 5 | 0.86 | 0.214 | 0.87 | 0.374 | 0.73 | 0.060 | 0.91 | 0.405 | 0.71 | 0.126 | 0.93 | 0.398 | 0.77 | 0.230 |
| P18124 | 60S ribosomal protein L7 | 5 | 0.67 | 0.137 | 0.65 | 0.128 | 0.72 | 0.057 | 0.98 | 0.472 | 0.95 | 0.431 | **0.59** | **0.044** | **0.75** | **0.012** |
| P21796 | Voltage-dependent anion-selective channel protein 1 | 5 | 0.86 | 0.340 | 0.88 | 0.310 | 0.80 | 0.156 | 0.93 | 0.374 | 0.98 | 0.473 | 0.72 | 0.167 | 1.00 | 0.487 |
| P22314 | Ubiquitin-like modifier-activating enzyme 1 | 5 | 1.79 | 0.123 | 1.37 | 0.204 | **2.14** | **0.033** | 1.25 | 0.257 | **2.06** | **0.047** | 1.61 | 0.216 | 1.56 | 0.134 |
| P25787 | Proteasome subunit alpha type-2 | 5 | 0.81 | 0.243 | 0.74 | 0.204 | 1.06 | 0.390 | 0.84 | 0.180 | 0.86 | 0.387 | 1.08 | 0.336 | 1.11 | 0.415 |
| P26038 | Moesin | 5 | 0.82 | 0.161 | 1.11 | 0.407 | 0.80 | 0.161 | 0.91 | 0.398 | 0.74 | 0.295 | 0.78 | 0.315 | 0.87 | 0.387 |
| P27797 | Calreticulin | 5 | 1.31 | 0.199 | 1.08 | 0.425 | 1.35 | 0.150 | 1.08 | 0.424 | 1.39 | 0.240 | 1.04 | 0.451 | 1.43 | 0.167 |
| P30101 | Protein disulfide-isomerase A3 | 5 | 1.16 | 0.378 | 1.05 | 0.435 | 1.05 | 0.414 | 1.09 | 0.402 | 1.13 | 0.299 | 1.28 | 0.162 | 1.14 | 0.273 |
| P37802 | Transgelin-2 | 5 | 0.77 | 0.173 | **0.70** | **0.035** | **0.68** | **0.028** | 0.95 | 0.430 | 0.81 | 0.107 | 0.73 | 0.100 | 0.79 | 0.166 |
| P38919 | Eukaryotic initiation factor 4A-III | 5 | 0.92 | 0.333 | 0.90 | 0.423 | 1.14 | 0.401 | **0.73** | **0.025** | **1.38** | **0.025** | 0.77 | 0.289 | 1.43 | 0.207 |
| P46781 | 40S ribosomal protein S9 | 5 | **0.71** | **0.020** | 0.86 | 0.193 | 0.74 | 0.113 | 0.93 | 0.372 | 0.87 | 0.296 | 0.75 | 0.136 | 0.83 | 0.163 |
| P49368 | T-complex protein 1 subunit gamma | 5 | 1.01 | 0.498 | 1.07 | 0.444 | 1.07 | 0.441 | 0.99 | 0.485 | 1.12 | 0.407 | 0.99 | 0.490 | 1.02 | 0.482 |
| P50914 | 60S ribosomal protein L14 | 5 | 1.16 | 0.344 | 1.36 | 0.195 | 1.18 | 0.295 | 0.98 | 0.475 | 1.30 | 0.248 | 1.13 | 0.350 | 1.27 | 0.223 |
| P54136 | Arginine--tRNA ligase. cytoplasmic | 5 | 0.95 | 0.383 | 1.14 | 0.241 | 1.07 | 0.393 | 1.01 | 0.478 | 1.19 | 0.252 | 1.04 | 0.434 | 1.14 | 0.223 |
| P61247 | 40S ribosomal protein S3a | 5 | 1.74 | 0.278 | 1.11 | 0.379 | 2.09 | 0.262 | 0.97 | 0.458 | 1.08 | 0.436 | 1.82 | 0.282 | 1.06 | 0.440 |
| P61313 | 60S ribosomal protein L15 | 5 | 0.94 | 0.441 | 1.19 | 0.363 | 1.07 | 0.430 | 1.11 | 0.361 | 1.19 | 0.378 | 0.82 | 0.331 | 1.02 | 0.478 |
| P62249 | 40S ribosomal protein S16 | 5 | 0.81 | 0.146 | 0.89 | 0.261 | **0.80** | **0.036** | 1.03 | 0.455 | 0.87 | 0.258 | 0.75 | 0.116 | 0.89 | 0.282 |
| P62979 | Ubiquitin-40S ribosomal protein S27a | 5 | 0.75 | 0.198 | 0.86 | 0.286 | 0.70 | 0.102 | 0.77 | 0.165 | 0.69 | 0.099 | **0.61** | **0.045** | 0.84 | 0.263 |
| Q06830 | Peroxiredoxin-1 | 5 | 0.86 | 0.201 | 0.93 | 0.346 | 0.89 | 0.317 | 0.88 | 0.251 | 0.84 | 0.157 | **0.77** | **0.030** | 0.96 | 0.419 |
| Q13283 | Ras GTPase-activating protein-binding protein 1 | 5 | **0.72** | **0.037** | 0.85 | 0.318 | 0.74 | 0.246 | 1.00 | 0.482 | 0.75 | 0.190 | 0.65 | 0.168 | 0.84 | 0.214 |
| Q15084 | Protein disulfide-isomerase A6 | 5 | 0.87 | 0.353 | 0.90 | 0.367 | 0.90 | 0.373 | 0.90 | 0.373 | 0.94 | 0.409 | 0.83 | 0.296 | 0.94 | 0.422 |
| Q7KZF4 | Staphylococcal nuclease domain-containing protein 1 | 5 | 0.97 | 0.483 | 1.00 | 0.498 | 0.91 | 0.454 | 0.91 | 0.453 | 0.99 | 0.494 | 1.15 | 0.417 | 2.01 | 0.086 |
| O00148 | ATP-dependent RNA helicase DDX39A | 4 | 1.36 | 0.055 | **1.53** | **0.034** | **1.47** | **0.046** | 1.32 | 0.220 | **1.88** | **0.000** | **1.55** | **0.010** | **1.66** | **0.001** |
| O75874 | Isocitrate dehydrogenase [NADP] cytoplasmic | 4 | 1.28 | 0.228 | 1.08 | 0.397 | 1.28 | 0.281 | 1.01 | 0.494 | 1.17 | 0.381 | 1.02 | 0.483 | 1.17 | 0.332 |
| O76003 | Glutaredoxin-3 | 4 | 0.89 | 0.439 | 1.12 | 0.344 | 1.18 | 0.331 | 1.25 | 0.306 | 1.29 | 0.240 | 1.14 | 0.401 | 1.25 | 0.158 |
| O95573 | Long-chain-fatty-acid--CoA ligase 3 | 4 | **2.15** | **0.010** | 1.27 | 0.197 | 1.68 | 0.065 | 1.11 | 0.353 | **2.11** | **0.000** | 1.12 | 0.359 | **2.03** | **0.001** |
| O95831 | Apoptosis-inducing factor 1. mitochondrial | 4 | 1.11 | 0.410 | 1.71 | 0.237 | 1.18 | 0.329 | 1.09 | 0.462 | 0.87 | 0.376 | 1.14 | 0.390 | 1.82 | 0.082 |
| P00491 | Purine nucleoside phosphorylase | 4 | 1.34 | 0.105 | 1.14 | 0.276 | **1.61** | **0.028** | 1.11 | 0.309 | 1.24 | 0.215 | 1.16 | 0.248 | **1.56** | **0.030** |
| P07195 | L-lactate dehydrogenase B chain | 4 | **0.67** | **0.013** | 0.80 | 0.213 | **0.58** | **0.000** | 0.80 | 0.090 | 0.62 | 0.082 | **0.63** | **0.000** | **0.79** | **0.014** |
| P08758 | Annexin A5 | 4 | 1.27 | 0.182 | 1.37 | 0.089 | 1.46 | 0.095 | 1.23 | 0.145 | 1.38 | 0.116 | 1.31 | 0.174 | 1.43 | 0.069 |
| P09525 | Annexin A4 | 4 | 0.67 | 0.057 | 0.80 | 0.224 | 0.81 | 0.317 | 1.07 | 0.423 | 0.68 | 0.055 | 0.81 | 0.325 | 0.89 | 0.334 |
| P10412 | Histone H1.4 | 4 | 0.46 | 0.075 | **0.40** | **0.040** | **0.32** | **0.018** | 0.56 | 0.124 | **0.31** | **0.023** | 0.52 | 0.102 | 0.71 | 0.251 |
| P17987 | T-complex protein 1 subunit alpha | 4 | 0.79 | 0.315 | 1.00 | 0.495 | 0.78 | 0.245 | 0.81 | 0.160 | 0.72 | 0.172 | 0.83 | 0.275 | 0.94 | 0.398 |
| P20742 | Pregnancy zone protein | 4 | 4.45 | 0.093 | 1.26 | 0.346 | **7.11** | **0.026** | 1.22 | 0.307 | 0.74 | 0.113 | **6.60** | **0.039** | 1.45 | 0.242 |
| P21281 | V-type proton ATPase subunit B. brain isoform | 4 | 1.00 | 0.499 | 1.07 | 0.430 | 1.08 | 0.325 | 0.95 | 0.384 | 1.39 | 0.056 | 1.26 | 0.123 | 1.00 | 0.495 |
| P21980 | Protein-glutamine gamma-glutamyltransferase 2 | 4 | 1.13 | 0.334 | 0.85 | 0.297 | 0.99 | 0.485 | 0.94 | 0.427 | 0.99 | 0.484 | 0.82 | 0.321 | 1.07 | 0.409 |
| P22102 | Trifunctional purine biosynthetic protein adenosine-3 | 4 | 0.92 | 0.327 | 0.91 | 0.417 | 0.95 | 0.431 | 1.06 | 0.436 | 0.64 | 0.128 | 0.96 | 0.437 | 0.81 | 0.253 |
| P22392 | Nucleoside diphosphate kinase B | 4 | 1.59 | 0.309 | 1.29 | 0.395 | 0.61 | 0.317 | 1.31 | 0.389 | 0.84 | 0.432 | 2.39 | 0.165 | 0.88 | 0.451 |
| P25786 | Proteasome subunit alpha type-1 | 4 | 0.85 | 0.090 | 0.92 | 0.308 | 0.86 | 0.341 | 0.88 | 0.132 | **0.65** | **0.039** | 0.81 | 0.115 | 0.98 | 0.424 |
| P26006 | Integrin alpha-3 | 4 | 0.86 | 0.284 | 0.91 | 0.235 | 0.86 | 0.221 | 1.08 | 0.305 | 0.82 | 0.069 | 0.89 | 0.316 | 0.83 | 0.163 |
| P26599 | Polypyrimidine tract-binding protein 1 | 4 | 0.73 | 0.129 | 1.04 | 0.459 | 0.84 | 0.321 | 1.06 | 0.430 | 0.86 | 0.203 | 0.81 | 0.295 | 0.94 | 0.428 |
| P31948 | Stress-induced-phosphoprotein 1 | 4 | 1.07 | 0.436 | 1.40 | 0.084 | 1.24 | 0.277 | 1.00 | 0.497 | 1.13 | 0.345 | 1.01 | 0.486 | 1.28 | 0.277 |
| P35241 | Radixin | 4 | 0.84 | 0.323 | 0.87 | 0.355 | 0.67 | 0.155 | 0.79 | 0.273 | **0.47** | **0.043** | 1.04 | 0.463 | 0.73 | 0.213 |
| P39019 | 40S ribosomal protein S19 | 4 | **0.84** | **0.038** | 0.88 | 0.103 | 0.85 | 0.102 | 0.93 | 0.193 | **0.75** | **0.018** | **0.63** | **0.001** | 0.94 | 0.381 |
| P45880 | Voltage-dependent anion-selective channel protein 2 | 4 | 1.06 | 0.415 | 1.15 | 0.289 | 0.98 | 0.446 | 1.07 | 0.333 | 1.15 | 0.264 | 0.87 | 0.250 | 1.17 | 0.278 |
| P46783 | 40S ribosomal protein S10 | 4 | **1.42** | **0.020** | 1.35 | 0.081 | **1.52** | **0.020** | 1.29 | 0.084 | **1.64** | **0.006** | 1.36 | 0.096 | 1.44 | 0.071 |
| P47897 | Glutamine--tRNA ligase | 4 | 1.48 | 0.136 | 1.49 | 0.206 | 1.89 | 0.141 | 1.53 | 0.189 | 1.96 | 0.068 | 1.46 | 0.221 | 1.87 | 0.134 |
| P48444 | Coatomer subunit delta | 4 | 1.53 | 0.264 | 1.47 | 0.148 | 1.54 | 0.192 | 1.21 | 0.067 | 1.24 | 0.355 | 1.41 | 0.212 | 1.31 | 0.289 |
| P50995 | Annexin A11 | 4 | 0.80 | 0.145 | 0.84 | 0.347 | **0.67** | **0.021** | 0.85 | 0.332 | 0.68 | 0.172 | 0.82 | 0.193 | 0.82 | 0.162 |
| P55209 | Nucleosome assembly protein 1-like 1 | 4 | 1.22 | 0.341 | 1.23 | 0.299 | 1.24 | 0.318 | 1.28 | 0.169 | 1.57 | 0.229 | 1.33 | 0.265 | 1.21 | 0.342 |
| P61026 | Ras-related protein Rab-10 | 4 | 1.00 | 0.497 | 1.25 | 0.231 | 1.18 | 0.312 | 1.05 | 0.439 | 1.00 | 0.493 | 1.02 | 0.479 | 1.16 | 0.349 |
| P62826 | GTP-binding nuclear protein Ran | 4 | 1.03 | 0.463 | 1.21 | 0.128 | **1.55** | **0.000** | **1.29** | **0.046** | **1.57** | **0.013** | **1.49** | **0.005** | **1.42** | **0.010** |
| P63244 | Guanine nucleotide-binding protein subunit beta-2-like 1 | 4 | **0.59** | **0.000** | 0.67 | 0.064 | **0.71** | **0.038** | 0.86 | 0.292 | **0.72** | **0.007** | **0.53** | **0.000** | 0.93 | 0.279 |
| Q01082 | Spectrin beta chain. non-erythrocytic 1 | 4 | 0.82 | 0.374 | 0.92 | 0.423 | 0.81 | 0.362 | 0.88 | 0.384 | 0.96 | 0.462 | 0.80 | 0.230 | 0.89 | 0.419 |
| Q08257 | Quinone oxidoreductase | 4 | **0.66** | **0.027** | 0.81 | 0.074 | 0.83 | 0.194 | 1.09 | 0.326 | 0.74 | 0.226 | 0.97 | 0.410 | **0.73** | **0.025** |
| Q12797 | Aspartyl/asparaginyl beta-hydroxylase | 4 | 1.02 | 0.487 | 1.21 | 0.192 | 1.16 | 0.364 | 1.25 | 0.236 | 1.04 | 0.470 | 1.32 | 0.246 | 1.36 | 0.122 |
| Q13838 | Spliceosome RNA helicase DDX39B | 4 | **2.12** | **0.000** | **1.80** | **0.013** | **1.56** | **0.006** | 1.38 | 0.109 | **2.00** | **0.000** | 1.49 | 0.055 | **1.69** | **0.019** |
| Q14152 | Eukaryotic translation initiation factor 3 subunit A | 4 | 0.93 | 0.428 | 1.08 | 0.410 | 1.02 | 0.477 | 1.13 | 0.375 | 0.89 | 0.385 | 1.00 | 0.493 | 0.99 | 0.482 |
| Q15008 | 26S proteasome non-ATPase regulatory subunit 6 | 4 | 0.92 | 0.398 | 1.01 | 0.490 | 1.13 | 0.173 | 0.94 | 0.296 | 1.03 | 0.477 | 1.33 | 0.090 | 0.98 | 0.467 |
| Q16401 | 26S proteasome non-ATPase regulatory subunit 5 | 4 | 0.95 | 0.459 | 1.18 | 0.366 | 1.13 | 0.378 | 1.07 | 0.447 | 1.02 | 0.491 | 1.08 | 0.454 | 0.73 | 0.241 |
| Q99623 | Prohibitin-2 | 4 | 1.26 | 0.126 | 1.22 | 0.212 | 1.20 | 0.215 | 1.03 | 0.416 | 1.31 | 0.124 | 0.97 | 0.419 | 1.17 | 0.218 |
| Q99714 | 3-hydroxyacyl-CoA dehydrogenase type-2 | 4 | 1.50 | 0.184 | 1.17 | 0.317 | 1.26 | 0.283 | 0.95 | 0.430 | 1.38 | 0.282 | 1.20 | 0.314 | 1.26 | 0.162 |
| Q9Y265 | RuvB-like 1 | 4 | 1.00 | 0.491 | 1.03 | 0.477 | 1.15 | 0.305 | 1.04 | 0.457 | 1.07 | 0.392 | 1.09 | 0.427 | 1.05 | 0.441 |
| O00159 | Unconventional myosin-Ic | 3 | 1.00 | 0.492 | 0.86 | 0.328 | 1.09 | 0.272 | 0.99 | 0.433 | 0.95 | 0.405 | 1.03 | 0.451 | 0.92 | 0.388 |
| O00303 | Eukaryotic translation initiation factor 3 subunit F | 3 | 0.70 | 0.217 | 0.83 | 0.139 | 0.89 | 0.302 | 0.81 | 0.283 | 0.78 | 0.327 | 0.87 | 0.232 | 0.64 | 0.079 |
| O14980 | Exportin-1 | 3 | 1.00 | 0.494 | 1.53 | 0.324 | 1.27 | 0.278 | 1.33 | 0.312 | 0.90 | 0.316 | 1.57 | 0.192 | 1.13 | 0.425 |
| O43242 | 26S proteasome non-ATPase regulatory subunit 3 | 3 | 1.02 | 0.490 | 1.04 | 0.459 | 1.07 | 0.459 | 1.12 | 0.383 | 1.07 | 0.466 | 1.09 | 0.441 | 0.93 | 0.450 |
| O43747 | AP-1 complex subunit gamma-1 | 3 | 0.82 | 0.231 | 0.94 | 0.370 | 0.92 | 0.325 | 1.00 | 0.481 | 1.13 | 0.326 | 0.87 | 0.194 | 1.16 | 0.153 |
| O75131 | Copine-3 | 3 | 1.02 | 0.484 | 1.05 | 0.413 | 1.21 | 0.112 | 1.33 | 0.113 | 0.96 | 0.401 | 1.08 | 0.425 | 1.05 | 0.386 |
| O75533 | Splicing factor 3B subunit 1 | 3 | 0.74 | 0.166 | 0.87 | 0.340 | **0.69** | **0.007** | 0.76 | 0.149 | 0.68 | 0.131 | 0.81 | 0.264 | **0.56** | **0.001** |
| O94925 | Glutaminase kidney isoform. mitochondrial | 3 | 1.23 | 0.173 | 0.86 | 0.307 | **1.53** | **0.017** | 1.03 | 0.458 | 1.11 | 0.399 | 0.99 | 0.490 | 1.22 | 0.344 |
| O95433 | Activator of 90 kDa heat shock protein ATPase homolog 1 | 3 | **0.64** | **0.048** | 1.08 | 0.334 | 0.57 | 0.126 | 0.82 | 0.288 | **0.59** | **0.005** | 0.73 | 0.166 | **0.63** | **0.039** |
| O95994 | Anterior gradient protein 2 homolog | 3 | 0.83 | 0.215 | 1.02 | 0.481 | 0.91 | 0.338 | 0.93 | 0.378 | 0.82 | 0.134 | 0.92 | 0.358 | 0.95 | 0.399 |
| P00505 | Aspartate aminotransferase. mitochondrial | 3 | 0.88 | 0.368 | 0.98 | 0.471 | 0.87 | 0.263 | 0.80 | 0.293 | 0.83 | 0.321 | 0.93 | 0.398 | 1.03 | 0.462 |
| P02786 | Transferrin receptor protein 1 | 3 | 1.28 | 0.222 | 1.11 | 0.391 | 1.19 | 0.348 | 1.53 | 0.174 | 2.02 | 0.093 | 1.38 | 0.249 | 1.45 | 0.244 |
| P04181 | Ornithine aminotransferase. mitochondrial | 3 | 1.45 | 0.228 | 1.03 | 0.452 | **2.03** | **0.047** | 0.87 | 0.387 | 1.26 | 0.355 | 0.78 | 0.245 | 1.73 | 0.119 |
| P04844 | Dolichyl-diphosphooligosaccharide--protein glycosyltransferase subunit 2 | 3 | 1.30 | 0.274 | 1.28 | 0.278 | 0.97 | 0.473 | 1.16 | 0.313 | 1.17 | 0.334 | 1.22 | 0.366 | 1.00 | 0.496 |
| P07910 | Heterogeneous nuclear ribonucleoproteins C1/C2 | 3 | 1.19 | 0.219 | 1.21 | 0.261 | 1.14 | 0.324 | 1.09 | 0.378 | 1.31 | 0.132 | 1.06 | 0.422 | 1.28 | 0.173 |
| P08865 | 40S ribosomal protein SA | 3 | **0.59** | **0.018** | 0.73 | 0.231 | 0.92 | 0.454 | 0.81 | 0.346 | 0.71 | 0.291 | 0.79 | 0.393 | **0.69** | **0.013** |
| P09382 | Galectin-1 | 3 | 0.85 | 0.172 | 0.77 | 0.127 | 0.83 | 0.167 | 0.88 | 0.286 | 0.86 | 0.247 | **0.67** | **0.024** | 0.81 | 0.187 |
| P12956 | X-ray repair cross-complementing protein 6 | 3 | 1.31 | 0.363 | 1.36 | 0.298 | 1.67 | 0.101 | 1.36 | 0.269 | 1.66 | 0.147 | 1.36 | 0.259 | 1.52 | 0.265 |
| P13010 | X-ray repair cross-complementing protein 5 | 3 | 0.91 | 0.354 | 1.02 | 0.473 | 0.88 | 0.384 | 1.05 | 0.449 | 1.06 | 0.368 | 1.20 | 0.165 | 1.00 | 0.494 |
| P14550 | Alcohol dehydrogenase [NADP(+)] | 3 | 0.70 | 0.233 | 0.99 | 0.492 | 0.78 | 0.299 | 0.54 | 0.108 | 0.68 | 0.219 | 0.64 | 0.179 | 0.79 | 0.311 |
| P16152 | Carbonyl reductase [NADPH] 1 | 3 | 1.02 | 0.491 | 0.86 | 0.380 | 1.02 | 0.490 | 0.94 | 0.378 | **0.77** | **0.015** | 1.12 | 0.431 | 0.93 | 0.399 |
| P16435 | NADPH--cytochrome P450 reductase | 3 | 1.14 | 0.362 | 0.86 | 0.234 | 1.14 | 0.334 | 0.79 | 0.314 | 1.13 | 0.382 | 0.95 | 0.408 | 1.28 | 0.129 |
| P19338 | Nucleolin | 3 | 0.82 | 0.192 | 0.82 | 0.252 | 0.88 | 0.243 | 0.81 | 0.269 | 0.81 | 0.374 | 0.96 | 0.471 | 0.71 | 0.189 |
| P22695 | Cytochrome b-c1 complex subunit 2. mitochondrial | 3 | 0.87 | 0.294 | 0.83 | 0.188 | 0.87 | 0.378 | 1.18 | 0.236 | 1.39 | 0.092 | 0.94 | 0.449 | 1.24 | 0.245 |
| P25705 | ATP synthase subunit alpha. mitochondrial | 3 | 1.06 | 0.449 | 0.99 | 0.484 | 1.09 | 0.413 | 0.93 | 0.428 | 1.06 | 0.450 | 0.96 | 0.455 | 1.16 | 0.352 |
| P26373 | 60S ribosomal protein L13 | 3 | **0.81** | **0.049** | 0.78 | 0.124 | 0.81 | 0.131 | 0.84 | 0.159 | 0.83 | 0.169 | 0.73 | 0.077 | **0.79** | **0.013** |
| P26447 | Protein S100-A4 | 3 | 0.99 | 0.484 | 1.26 | 0.226 | 1.10 | 0.386 | 1.35 | 0.173 | 0.96 | 0.373 | 1.00 | 0.491 | 1.16 | 0.320 |
| P27824 | Calnexin | 3 | 1.27 | 0.216 | 1.19 | 0.264 | 1.34 | 0.172 | 1.15 | 0.292 | 1.40 | 0.139 | 1.13 | 0.319 | **1.45** | **0.027** |
| P30041 | Peroxiredoxin-6 | 3 | 0.81 | 0.190 | 1.02 | 0.473 | 0.88 | 0.339 | 0.85 | 0.254 | 0.92 | 0.269 | 0.86 | 0.242 | 0.98 | 0.457 |
| P30046 | D-dopachrome decarboxylase | 3 | 1.30 | 0.245 | 1.06 | 0.451 | 1.05 | 0.455 | 1.10 | 0.370 | 0.99 | 0.489 | 0.86 | 0.327 | 1.13 | 0.372 |
| P30086 | Phosphatidylethanolamine-binding protein 1 | 3 | **2.28** | **0.001** | **2.08** | **0.020** | **2.09** | **0.017** | 1.56 | 0.080 | **2.23** | **0.010** | **1.66** | **0.014** | **2.35** | **0.003** |
| P30740 | Leukocyte elastase inhibitor | 3 | 1.28 | 0.379 | 1.13 | 0.421 | 1.36 | 0.310 | 1.22 | 0.361 | 1.50 | 0.311 | 1.33 | 0.289 | 1.44 | 0.315 |
| P31939 | Bifunctional purine biosynthesis protein PURH | 3 | 0.95 | 0.424 | 1.04 | 0.431 | 1.63 | 0.143 | 1.02 | 0.471 | 0.68 | 0.060 | 1.62 | 0.218 | 0.89 | 0.298 |
| P34932 | Heat shock 70 kDa protein 4 | 3 | 0.57 | 0.173 | 0.92 | 0.434 | 0.75 | 0.376 | 0.62 | 0.304 | 0.73 | 0.265 | 0.81 | 0.350 | 0.72 | 0.204 |
| P36578 | 60S ribosomal protein L4 | 3 | 1.40 | 0.326 | 1.12 | 0.367 | 1.78 | 0.238 | 0.98 | 0.477 | 0.98 | 0.476 | 1.57 | 0.288 | 0.94 | 0.417 |
| P39023 | 60S ribosomal protein L3 | 3 | 0.82 | 0.268 | 0.81 | 0.334 | 0.85 | 0.371 | 1.07 | 0.349 | 0.80 | 0.296 | 0.81 | 0.331 | 0.88 | 0.387 |
| P40939 | Trifunctional enzyme subunit alpha. mitochondrial | 3 | 1.31 | 0.260 | 1.20 | 0.272 | 1.40 | 0.133 | 1.10 | 0.401 | 1.45 | 0.176 | 1.20 | 0.279 | 1.37 | 0.103 |
| P41250 | Glycine--tRNA ligase | 3 | 1.38 | 0.200 | **1.57** | **0.000** | 1.50 | 0.186 | **1.35** | **0.027** | 1.64 | 0.076 | 1.45 | 0.202 | **1.53** | **0.021** |
| P41252 | Isoleucine--tRNA ligase. cytoplasmic | 3 | 0.77 | 0.190 | 0.92 | 0.381 | 0.83 | 0.239 | 0.82 | 0.259 | 0.83 | 0.285 | 0.83 | 0.302 | 0.89 | 0.331 |
| P42166 | Lamina-associated polypeptide 2. isoform alpha | 3 | 1.24 | 0.373 | 1.38 | 0.238 | 1.02 | 0.478 | 1.19 | 0.191 | 1.25 | 0.360 | 1.31 | 0.337 | 1.28 | 0.293 |
| P43243 | Matrin-3 | 3 | 1.04 | 0.463 | 0.95 | 0.450 | 0.93 | 0.385 | 1.28 | 0.177 | 1.01 | 0.493 | 1.00 | 0.491 | 1.14 | 0.352 |
| P46379 | Large proline-rich protein BAG6 | 3 | 1.75 | 0.201 | 1.38 | 0.293 | 1.10 | 0.442 | 1.04 | 0.438 | 1.30 | 0.263 | 1.15 | 0.401 | 1.08 | 0.455 |
| P46776 | 60S ribosomal protein L27a | 3 | 0.91 | 0.389 | 0.96 | 0.403 | 0.92 | 0.369 | 1.14 | 0.339 | 0.92 | 0.385 | 0.91 | 0.381 | 0.97 | 0.445 |
| P48643 | T-complex protein 1 subunit epsilon | 3 | 1.58 | 0.181 | 1.66 | 0.063 | 1.54 | 0.182 | 1.18 | 0.318 | 1.63 | 0.135 | 1.36 | 0.124 | 1.56 | 0.156 |
| P49411 | Elongation factor Tu. mitochondrial | 3 | 0.96 | 0.452 | 1.00 | 0.499 | 1.19 | 0.283 | 0.95 | 0.426 | 1.03 | 0.480 | 1.02 | 0.480 | 1.17 | 0.232 |
| P50395 | Rab GDP dissociation inhibitor beta | 3 | 0.86 | 0.250 | 0.97 | 0.436 | 1.14 | 0.362 | 0.95 | 0.429 | 1.11 | 0.354 | 1.08 | 0.374 | 1.07 | 0.429 |
| P50990 | T-complex protein 1 subunit theta | 3 | 0.69 | 0.062 | 0.74 | 0.236 | 0.87 | 0.356 | 0.52 | 0.217 | 0.89 | 0.372 | 0.91 | 0.423 | 0.83 | 0.291 |
| P51648 | Fatty aldehyde dehydrogenase | 3 | **0.69** | **0.033** | 0.93 | 0.344 | 0.72 | 0.093 | 0.65 | 0.084 | 0.85 | 0.199 | 0.94 | 0.354 | 0.74 | 0.063 |
| P51991 | Heterogeneous nuclear ribonucleoprotein A3 | 3 | **0.50** | **0.011** | 0.71 | 0.117 | **0.53** | **0.021** | **0.67** | **0.009** | **0.51** | **0.000** | **0.54** | **0.000** | **0.56** | **0.034** |
| P52272 | Heterogeneous nuclear ribonucleoprotein M | 3 | 1.52 | 0.255 | 1.48 | 0.297 | 1.39 | 0.282 | 1.15 | 0.361 | 1.46 | 0.339 | 1.31 | 0.327 | 1.30 | 0.302 |
| P55265 | Double-stranded RNA-specific adenosine deaminase | 3 | 1.18 | 0.310 | 1.08 | 0.440 | 1.38 | 0.194 | 1.27 | 0.127 | 1.39 | 0.212 | 1.41 | 0.234 | 1.27 | 0.338 |
| P55786 | Puromycin-sensitive aminopeptidase | 3 | 1.04 | 0.422 | 0.92 | 0.201 | 1.08 | 0.393 | 1.04 | 0.440 | **0.74** | **0.004** | 1.09 | 0.408 | 0.79 | 0.194 |
| P55884 | Eukaryotic translation initiation factor 3 subunit B | 3 | 0.73 | 0.214 | 0.71 | 0.204 | **0.40** | **0.015** | 0.87 | 0.354 | 0.79 | 0.277 | 0.68 | 0.170 | 0.81 | 0.299 |
| P56134 | ATP synthase subunit f. mitochondrial | 3 | 1.20 | 0.327 | 0.95 | 0.450 | 1.15 | 0.399 | 1.12 | 0.379 | 1.10 | 0.423 | 1.39 | 0.148 | 1.16 | 0.374 |
| P60842 | Eukaryotic initiation factor 4A-I | 3 | 0.82 | 0.169 | **0.67** | **0.011** | 0.74 | 0.120 | 0.79 | 0.172 | 0.89 | 0.322 | 0.68 | 0.052 | 0.75 | 0.112 |
| P61006 | Ras-related protein Rab-8A | 3 | 1.24 | 0.370 | 1.05 | 0.474 | 1.46 | 0.269 | 1.09 | 0.447 | 1.98 | 0.132 | 1.12 | 0.432 | 0.83 | 0.391 |
| P62269 | 40S ribosomal protein S18 | 3 | 1.14 | 0.287 | 1.26 | 0.251 | 1.12 | 0.310 | 1.16 | 0.307 | 1.24 | 0.152 | 1.14 | 0.279 | 1.17 | 0.310 |
| P62424 | 60S ribosomal protein L7a | 3 | **0.66** | **0.024** | **0.76** | **0.002** | **0.73** | **0.006** | 0.92 | 0.254 | 0.75 | 0.137 | 0.57 | 0.056 | 0.81 | 0.233 |
| P62701 | 40S ribosomal protein S4. X isoform | 3 | 0.81 | 0.094 | 0.92 | 0.384 | 0.89 | 0.265 | 1.03 | 0.449 | 0.84 | 0.219 | 0.78 | 0.096 | 0.93 | 0.383 |
| P83731 | 60S ribosomal protein L24 | 3 | **0.83** | **0.023** | 0.85 | 0.156 | 0.79 | 0.119 | 1.10 | 0.269 | 0.84 | 0.185 | 0.76 | 0.124 | 0.87 | 0.162 |
| P98179 | RNA-binding protein 3 | 3 | 0.66 | 0.134 | 0.52 | 0.173 | **0.45** | **0.019** | 1.30 | 0.236 | 0.77 | 0.239 | 0.62 | 0.106 | 0.69 | 0.166 |
| Q00839 | Heterogeneous nuclear ribonucleoprotein U | 3 | 0.79 | 0.258 | 0.86 | 0.334 | 0.89 | 0.328 | 0.99 | 0.485 | 1.00 | 0.499 | 0.71 | 0.136 | 0.91 | 0.331 |
| Q12905 | Interleukin enhancer-binding factor 2 | 3 | 0.79 | 0.123 | 1.06 | 0.426 | 0.89 | 0.325 | 1.07 | 0.374 | 1.02 | 0.441 | 1.01 | 0.484 | 0.97 | 0.447 |
| Q12931 | Heat shock protein 75 kDa. mitochondrial | 3 | 1.13 | 0.406 | 1.20 | 0.357 | 0.54 | 0.111 | 1.10 | 0.423 | 0.90 | 0.416 | 0.67 | 0.213 | 0.92 | 0.430 |
| Q16181 | Septin-7 | 3 | 1.10 | 0.368 | 1.11 | 0.289 | 1.15 | 0.375 | 0.95 | 0.439 | 0.84 | 0.167 | 0.88 | 0.368 | 0.78 | 0.323 |
| Q6IS14 | Eukaryotic translation initiation factor 5A-1-like | 3 | **1.53** | **0.009** | **1.67** | **0.027** | **1.48** | **0.019** | 1.54 | 0.051 | **1.72** | **0.037** | 1.51 | 0.103 | **1.96** | **0.002** |
| Q6WCQ1 | Myosin phosphatase Rho-interacting protein | 3 | **0.57** | **0.025** | 0.83 | 0.128 | 0.77 | 0.276 | 0.89 | 0.292 | 0.78 | 0.377 | 0.90 | 0.429 | 0.84 | 0.270 |
| Q8IUE6 | Histone H2A type 2-B | 3 | 0.88 | 0.338 | 0.88 | 0.312 | 0.89 | 0.369 | 0.98 | 0.466 | 1.02 | 0.480 | 0.91 | 0.383 | 0.92 | 0.373 |
| Q8N163 | Cell cycle and apoptosis regulator protein 2 | 3 | 0.74 | 0.240 | 0.76 | 0.253 | 1.10 | 0.406 | 1.15 | 0.364 | 2.22 | 0.198 | 1.22 | 0.316 | 1.70 | 0.251 |
| Q8NC51 | Plasminogen activator inhibitor 1 RNA-binding protein | 3 | 0.97 | 0.459 | 1.06 | 0.365 | **0.76** | **0.029** | 1.05 | 0.465 | 1.04 | 0.468 | 0.77 | 0.347 | 0.87 | 0.361 |
| Q92616 | Translational activator GCN1 | 3 | 1.16 | 0.426 | 1.35 | 0.087 | 1.26 | 0.383 | 0.83 | 0.306 | 1.07 | 0.437 | 1.27 | 0.180 | 0.92 | 0.457 |
| Q92890 | Ubiquitin fusion degradation protein 1 homolog | 3 | 1.35 | 0.230 | 0.90 | 0.394 | 0.95 | 0.448 | 1.15 | 0.369 | 1.03 | 0.474 | 0.99 | 0.488 | 1.11 | 0.406 |
| Q96FW1 | Ubiquitin thioesterase OTUB1 | 3 | 0.85 | 0.410 | 0.87 | 0.446 | 0.72 | 0.372 | 0.90 | 0.453 | 0.63 | 0.343 | 0.82 | 0.403 | 0.86 | 0.443 |
| Q96PK6 | RNA-binding protein 14 | 3 | 0.70 | 0.136 | 0.88 | 0.218 | 0.72 | 0.144 | 0.84 | 0.125 | **0.58** | **0.002** | **0.68** | **0.031** | 0.75 | 0.134 |
| Q99832 | T-complex protein 1 subunit eta | 3 | 1.19 | 0.341 | 0.87 | 0.404 | 0.53 | 0.077 | 1.03 | 0.482 | 0.95 | 0.461 | 0.57 | 0.205 | 1.04 | 0.479 |
| Q9BQG0 | Myb-binding protein 1A | 3 | 1.59 | 0.101 | 1.32 | 0.285 | 0.90 | 0.351 | 1.07 | 0.446 | 0.89 | 0.431 | 1.48 | 0.256 | 1.05 | 0.462 |
| Q9NQC3 | Reticulon-4 | 3 | 1.11 | 0.314 | 0.98 | 0.478 | 0.94 | 0.396 | 0.98 | 0.479 | 1.07 | 0.428 | 1.03 | 0.471 | 1.05 | 0.461 |
| Q9NZM1 | Myoferlin | 3 | 1.20 | 0.355 | 1.29 | 0.283 | 1.20 | 0.339 | 0.92 | 0.428 | 1.59 | 0.285 | 1.43 | 0.212 | 1.36 | 0.279 |
| Q9UBT2 | SUMO-activating enzyme subunit 2 | 3 | 2.02 | 0.140 | 1.79 | 0.182 | 1.40 | 0.304 | 2.15 | 0.114 | 1.72 | 0.209 | 2.09 | 0.127 | 2.80 | 0.052 |
| Q9UL46 | Proteasome activator complex subunit 2 | 3 | 1.58 | 0.299 | 1.21 | 0.346 | **2.43** | **0.000** | 1.53 | 0.275 | 1.58 | 0.106 | 1.45 | 0.117 | 1.06 | 0.410 |
| Q9Y277 | Voltage-dependent anion-selective channel protein 3 | 3 | 1.24 | 0.155 | 0.93 | 0.381 | 1.04 | 0.445 | 1.03 | 0.460 | 1.20 | 0.286 | 0.90 | 0.252 | 1.11 | 0.272 |
| Q9Y6N5 | Sulfide:quinone oxidoreductase. mitochondrial | 3 | 1.00 | 0.496 | 1.18 | 0.214 | 1.05 | 0.425 | 1.04 | 0.455 | 1.04 | 0.452 | 1.00 | 0.485 | 1.05 | 0.435 |
| O00154 | Cytosolic acyl coenzyme A thioester hydrolase | 2 | **1.82** | **0.006** | 1.10 | 0.418 | 1.58 | 0.081 | 1.07 | 0.427 | **1.78** | **0.005** | 1.31 | 0.314 | 1.19 | 0.321 |
| O00273 | DNA fragmentation factor subunit alpha | 2 | 1.09 | 0.439 | 1.00 | 0.499 | 1.00 | 0.497 | 0.93 | 0.451 | 0.76 | 0.323 | 0.87 | 0.453 | 0.83 | 0.406 |
| O00469 | Procollagen-lysine.2-oxoglutarate 5-dioxygenase 2 | 2 | 1.75 | 0.105 | 1.21 | 0.154 | 1.25 | 0.310 | **2.06** | **0.045** | **2.44** | **0.003** | **1.96** | **0.024** | 1.82 | 0.072 |
| O00487 | 26S proteasome non-ATPase regulatory subunit 14 | 2 | 0.86 | 0.386 | 1.01 | 0.499 | 0.79 | 0.327 | 1.15 | 0.398 | 0.62 | 0.206 | 1.21 | 0.364 | 1.32 | 0.304 |
| O00764 | Pyridoxal kinase | 2 | 0.56 | 0.071 | 0.77 | 0.066 | **0.56** | **0.005** | 1.01 | 0.496 | **0.51** | **0.000** | 0.77 | 0.082 | 0.97 | 0.474 |
| O14737 | Programmed cell death protein 5 | 2 | 0.60 | 0.060 | 0.82 | 0.204 | 0.77 | 0.182 | 0.84 | 0.309 | 0.68 | 0.109 | **0.58** | **0.044** | 0.90 | 0.317 |
| O14745 | Na(+)/H(+) exchange regulatory cofactor NHE-RF1 | 2 | **2.51** | **0.026** | 1.53 | 0.238 | **2.24** | **0.044** | 0.89 | 0.407 | **4.62** | **0.000** | 1.12 | 0.413 | 1.68 | 0.150 |
| O15067 | Phosphoribosylformylglycinamidine synthase | 2 | 0.73 | 0.205 | 0.73 | 0.200 | 0.74 | 0.227 | 0.76 | 0.230 | 0.47 | 0.157 | 0.61 | 0.102 | 0.84 | 0.323 |
| O15260 | Surfeit locus protein 4 | 2 | 0.51 | 0.283 | 0.74 | 0.387 | 0.85 | 0.414 | 0.84 | 0.411 | 0.52 | 0.090 | 0.74 | 0.323 | 0.78 | 0.303 |
| O43143 | Pre-mRNA-splicing factor ATP-dependent RNA helicase DHX15 | 2 | 1.13 | 0.431 | 1.24 | 0.383 | 1.09 | 0.448 | 0.85 | 0.228 | 1.29 | 0.372 | 1.03 | 0.486 | 1.11 | 0.427 |
| O43169 | Cytochrome b5 type B | 2 | 1.37 | 0.368 | 1.38 | 0.326 | 1.35 | 0.377 | 0.97 | 0.436 | 1.79 | 0.297 | 1.44 | 0.372 | 1.19 | 0.387 |
| O60256 | Phosphoribosyl pyrophosphate synthase-associated protein 2 | 2 | 1.08 | 0.451 | 1.45 | 0.236 | 1.05 | 0.449 | 1.21 | 0.309 | 0.97 | 0.474 | 0.75 | 0.253 | 0.98 | 0.467 |
| O60506 | Heterogeneous nuclear ribonucleoprotein Q | 2 | 0.71 | 0.192 | 0.98 | 0.473 | 0.81 | 0.285 | 1.07 | 0.435 | 0.61 | 0.109 | 0.79 | 0.267 | 0.93 | 0.418 |
| O60664 | Perilipin-3 | 2 | 0.98 | 0.446 | 0.84 | 0.116 | 0.89 | 0.366 | 0.95 | 0.411 | 0.93 | 0.421 | 0.88 | 0.197 | 1.01 | 0.493 |
| O75083 | WD repeat-containing protein 1 | 2 | 1.45 | 0.080 | 1.58 | 0.071 | 1.02 | 0.464 | 1.27 | 0.223 | **1.30** | **0.014** | **1.69** | **0.005** | 1.26 | 0.278 |
| O75390 | Citrate synthase. mitochondrial | 2 | 1.24 | 0.057 | 1.15 | 0.172 | 1.18 | 0.157 | 0.99 | 0.464 | 1.24 | 0.144 | 0.97 | 0.414 | 1.25 | 0.131 |
| O75643 | U5 small nuclear ribonucleoprotein 200 kDa helicase | 2 | 1.08 | 0.462 | 0.79 | 0.372 | 1.40 | 0.289 | 0.81 | 0.378 | 1.56 | 0.214 | 1.44 | 0.259 | 0.98 | 0.487 |
| O95782 | AP-2 complex subunit alpha-1 | 2 | **1.78** | **0.031** | 1.29 | 0.228 | 1.71 | 0.128 | 1.19 | 0.261 | 1.43 | 0.172 | 1.43 | 0.209 | 1.48 | 0.181 |
| P00403 | Cytochrome c oxidase subunit 2 | 2 | 1.09 | 0.458 | 0.90 | 0.335 | 1.64 | 0.085 | 1.01 | 0.496 | **1.75** | **0.034** | 1.40 | 0.235 | 1.24 | 0.205 |
| P01024 | Complement C3 | 2 | 1.93 | 0.050 | 1.26 | 0.290 | 1.39 | 0.207 | 1.33 | 0.243 | 1.79 | 0.076 | 1.55 | 0.141 | 1.19 | 0.345 |
| P05556 | Integrin beta-1 | 2 | 0.81 | 0.212 | 0.99 | 0.460 | 0.84 | 0.158 | 1.01 | 0.492 | 0.83 | 0.223 | **0.77** | **0.013** | 0.99 | 0.476 |
| P06737 | Glycogen phosphorylase. liver form | 2 | 0.84 | 0.382 | 1.07 | 0.456 | 1.28 | 0.263 | 1.23 | 0.157 | 1.02 | 0.488 | 1.28 | 0.191 | 1.00 | 0.498 |
| P07099 | Epoxide hydrolase 1 | 2 | 0.80 | 0.091 | 0.99 | 0.478 | 1.02 | 0.470 | 0.83 | 0.124 | 1.04 | 0.433 | 0.84 | 0.144 | 0.96 | 0.452 |
| P07339 | Cathepsin D | 2 | 1.20 | 0.325 | 0.92 | 0.422 | 1.10 | 0.423 | 0.98 | 0.478 | 1.15 | 0.359 | 0.93 | 0.408 | 1.22 | 0.326 |
| P07741 | Adenine phosphoribosyltransferase | 2 | 0.95 | 0.429 | 0.84 | 0.261 | 0.91 | 0.347 | 1.04 | 0.445 | 0.97 | 0.442 | 1.05 | 0.436 | 0.90 | 0.364 |
| P09012 | U1 small nuclear ribonucleoprotein A | 2 | 1.08 | 0.448 | 1.15 | 0.397 | 1.26 | 0.332 | 1.63 | 0.176 | 0.72 | 0.287 | 1.31 | 0.310 | 1.59 | 0.193 |
| P09622 | Dihydrolipoyl dehydrogenase. mitochondrial | 2 | 1.47 | 0.202 | **1.48** | **0.037** | 1.04 | 0.429 | 1.05 | 0.419 | 1.51 | 0.099 | 0.93 | 0.360 | 1.03 | 0.456 |
| P09960 | Leukotriene A-4 hydrolase | 2 | 1.51 | 0.086 | 1.21 | 0.268 | 1.45 | 0.146 | 1.04 | 0.442 | **1.69** | **0.033** | 1.24 | 0.253 | 1.39 | 0.165 |
| P09972 | Fructose-bisphosphate aldolase C | 2 | **2.00** | **0.038** | 0.79 | 0.319 | **1.99** | **0.000** | 1.23 | 0.373 | **5.19** | **0.000** | 1.35 | 0.311 | **3.04** | **0.000** |
| P0CW22 | 40S ribosomal protein S17-like | 2 | 1.13 | 0.363 | 1.26 | 0.229 | 1.05 | 0.419 | 1.20 | 0.206 | 0.95 | 0.436 | 1.01 | 0.495 | 0.92 | 0.411 |
| P10599 | Thioredoxin | 2 | 1.03 | 0.474 | 1.24 | 0.293 | 1.13 | 0.398 | 1.29 | 0.261 | 1.07 | 0.415 | 0.99 | 0.487 | 1.32 | 0.278 |
| P10620 | Microsomal glutathione S-transferase 1 | 2 | 1.07 | 0.424 | 1.24 | 0.305 | 0.86 | 0.410 | 1.13 | 0.348 | 1.16 | 0.374 | 1.00 | 0.500 | 1.04 | 0.468 |
| P11388 | DNA topoisomerase 2-alpha | 2 | 0.71 | 0.268 | 0.76 | 0.283 | 0.72 | 0.260 | 0.82 | 0.362 | 1.13 | 0.436 | 0.56 | 0.135 | 0.77 | 0.319 |
| P12268 | Inosine-5'-monophosphate dehydrogenase 2 | 2 | 1.07 | 0.417 | 0.81 | 0.288 | 0.99 | 0.482 | 1.14 | 0.231 | 1.28 | 0.161 | 0.95 | 0.388 | 1.13 | 0.289 |
| P13667 | Protein disulfide-isomerase A4 | 2 | 0.89 | 0.257 | **0.70** | **0.022** | 0.86 | 0.276 | **0.66** | **0.042** | 0.77 | 0.093 | 0.92 | 0.303 | 0.77 | 0.228 |
| P14324 | Farnesyl pyrophosphate synthase | 2 | 0.99 | 0.488 | 0.78 | 0.264 | 0.69 | 0.170 | 0.92 | 0.408 | 0.92 | 0.414 | 0.70 | 0.190 | 1.02 | 0.485 |
| P16070 | CD44 antigen | 2 | **0.60** | **0.000** | 0.96 | 0.434 | **0.62** | **0.019** | 1.23 | 0.144 | 0.72 | 0.146 | **0.82** | **0.032** | 0.87 | 0.084 |
| P16949 | Stathmin | 2 | 1.06 | 0.431 | 1.14 | 0.372 | 1.06 | 0.455 | 0.95 | 0.445 | 0.88 | 0.271 | 0.84 | 0.288 | 1.26 | 0.317 |
| P17844 | Probable ATP-dependent RNA helicase DDX5 | 2 | **0.49** | **0.024** | 0.63 | 0.092 | **0.35** | **0.007** | 0.66 | 0.119 | 0.54 | 0.071 | 0.43 | 0.063 | **0.53** | **0.042** |
| P17858 | ATP-dependent 6-phosphofructokinase. liver type | 2 | **1.48** | **0.029** | **1.56** | **0.015** | 1.97 | 0.059 | **1.75** | **0.001** | **2.09** | **0.047** | **2.29** | **0.000** | 1.54 | 0.212 |
| P18621 | 60S ribosomal protein L17 | 2 | 1.27 | 0.246 | 1.49 | 0.221 | 1.34 | 0.210 | **1.51** | **0.001** | 1.16 | 0.396 | 1.24 | 0.313 | **1.41** | **0.004** |
| P21291 | Cysteine and glycine-rich protein 1 | 2 | **1.25** | **0.047** | 1.00 | 0.490 | 0.78 | 0.092 | 1.09 | 0.366 | **0.72** | **0.005** | **0.69** | **0.002** | 0.83 | 0.115 |
| P22087 | rRNA 2'-O-methyltransferase fibrillarin | 2 | 0.93 | 0.259 | 1.04 | 0.455 | 1.05 | 0.448 | 0.98 | 0.454 | 1.33 | 0.281 | 0.92 | 0.422 | 1.25 | 0.070 |
| P23284 | Peptidyl-prolyl cis-trans isomerase B | 2 | 1.29 | 0.275 | 1.01 | 0.493 | 1.20 | 0.363 | 0.89 | 0.274 | 1.21 | 0.386 | 1.10 | 0.432 | 1.22 | 0.247 |
| P23396 | 40S ribosomal protein S3 | 2 | 0.64 | 0.069 | 0.71 | 0.099 | **0.56** | **0.018** | **0.72** | **0.026** | 0.56 | 0.052 | 0.66 | 0.105 | 0.70 | 0.111 |
| P23526 | Adenosylhomocysteinase | 2 | **0.74** | **0.006** | 0.93 | 0.383 | 0.88 | 0.150 | 0.83 | 0.149 | 0.82 | 0.131 | 0.77 | 0.127 | 0.88 | 0.348 |
| P25205 | DNA replication licensing factor MCM3 | 2 | 0.86 | 0.375 | 0.99 | 0.483 | 1.11 | 0.231 | 1.07 | 0.422 | 0.97 | 0.438 | 0.86 | 0.240 | 0.94 | 0.419 |
| P27635 | 60S ribosomal protein L10 | 2 | 0.98 | 0.417 | 0.85 | 0.272 | 0.84 | 0.262 | 0.94 | 0.386 | 1.11 | 0.302 | 0.89 | 0.228 | 0.94 | 0.306 |
| P27695 | DNA-(apurinic or apyrimidinic site) lyase | 2 | 0.83 | 0.276 | 0.94 | 0.454 | 0.89 | 0.451 | 1.21 | 0.377 | 0.90 | 0.364 | 1.23 | 0.282 | 0.85 | 0.340 |
| P27708 | CAD protein | 2 | 0.71 | 0.210 | 0.82 | 0.270 | 0.93 | 0.417 | 1.00 | 0.498 | 0.88 | 0.395 | 0.85 | 0.334 | 0.88 | 0.386 |
| P28340 | DNA polymerase delta catalytic subunit | 2 | 1.11 | 0.414 | 1.11 | 0.415 | 0.74 | 0.373 | 0.94 | 0.437 | 1.01 | 0.496 | 0.98 | 0.483 | 0.79 | 0.362 |
| P30040 | Endoplasmic reticulum resident protein 29 | 2 | 1.04 | 0.390 | 1.12 | 0.338 | 1.40 | 0.171 | 1.07 | 0.408 | 1.17 | 0.251 | 1.02 | 0.483 | 1.25 | 0.204 |
| P30044 | Peroxiredoxin-5. mitochondrial | 2 | 1.13 | 0.356 | 1.03 | 0.446 | 1.14 | 0.381 | 1.03 | 0.478 | 1.25 | 0.292 | 1.43 | 0.171 | 1.31 | 0.278 |
| P30050 | 60S ribosomal protein L12 | 2 | 0.86 | 0.334 | 0.88 | 0.356 | 0.75 | 0.215 | 0.77 | 0.232 | 0.83 | 0.306 | 0.71 | 0.180 | 0.75 | 0.219 |
| P30153 | Serine/threonine-protein phosphatase 2A 65 kDa regulatory subunit A alpha isoform | 2 | 1.21 | 0.313 | 1.29 | 0.189 | 1.20 | 0.273 | 1.17 | 0.315 | 1.07 | 0.353 | 1.34 | 0.124 | 0.98 | 0.456 |
| P31483 | Nucleolysin TIA-1 isoform p40 | 2 | 1.18 | 0.387 | 1.29 | 0.321 | 0.93 | 0.448 | 1.52 | 0.223 | 1.24 | 0.353 | 1.03 | 0.481 | 1.23 | 0.360 |
| P33993 | DNA replication licensing factor MCM7 | 2 | 1.33 | 0.112 | 1.17 | 0.249 | 0.94 | 0.431 | 1.11 | 0.329 | 1.18 | 0.363 | 1.27 | 0.251 | 1.16 | 0.277 |
| P34897 | Serine hydroxymethyltransferase. mitochondrial | 2 | 0.85 | 0.348 | **0.76** | **0.023** | 0.77 | 0.268 | **0.60** | **0.001** | 0.72 | 0.169 | 0.65 | 0.121 | 0.95 | 0.434 |
| P35232 | Prohibitin | 2 | 1.12 | 0.406 | 0.91 | 0.423 | 0.85 | 0.389 | 0.87 | 0.374 | 1.10 | 0.429 | 0.88 | 0.393 | 1.05 | 0.459 |
| P35637 | RNA-binding protein FUS | 2 | 0.67 | 0.291 | 0.87 | 0.221 | 0.80 | 0.227 | 0.80 | 0.161 | 0.92 | 0.346 | 0.83 | 0.357 | **0.56** | **0.011** |
| P35998 | 26S protease regulatory subunit 7 | 2 | 0.90 | 0.380 | 0.77 | 0.235 | 0.72 | 0.188 | 0.79 | 0.262 | 0.63 | 0.159 | **0.51** | **0.046** | 0.64 | 0.130 |
| P37108 | Signal recognition particle 14 kDa protein | 2 | 1.19 | 0.329 | 1.14 | 0.357 | 1.18 | 0.316 | 1.15 | 0.323 | 1.32 | 0.180 | 1.13 | 0.375 | 1.29 | 0.207 |
| P38159 | RNA-binding motif protein. X chromosome | 2 | 0.88 | 0.354 | 0.90 | 0.154 | 0.72 | 0.126 | 0.99 | 0.482 | 0.79 | 0.303 | 0.88 | 0.352 | 0.83 | 0.158 |
| P39656 | Dolichyl-diphosphooligosaccharide--protein glycosyltransferase 48 kDa subunit | 2 | 0.64 | 0.133 | 0.75 | 0.253 | 0.79 | 0.111 | 0.92 | 0.384 | 0.89 | 0.376 | 0.87 | 0.173 | 0.95 | 0.427 |
| P40763 | Signal transducer and activator of transcription 3 | 2 | 1.58 | 0.180 | 0.84 | 0.366 | 1.51 | 0.199 | 0.98 | 0.483 | 1.06 | 0.462 | 1.20 | 0.404 | 1.31 | 0.315 |
| P41223 | Protein BUD31 homolog | 2 | 1.76 | 0.061 | 1.15 | 0.324 | **1.64** | **0.021** | 0.97 | 0.466 | **1.60** | **0.050** | 1.39 | 0.260 | **1.60** | **0.008** |
| P42224 | Signal transducer and activator of transcription 1-alpha/beta | 2 | 1.07 | 0.420 | 0.76 | 0.307 | 0.90 | 0.354 | 1.14 | 0.311 | 1.10 | 0.409 | 0.90 | 0.345 | 0.81 | 0.351 |
| P46777 | 60S ribosomal protein L5 | 2 | **0.67** | **0.000** | 0.83 | 0.162 | **0.61** | **0.021** | 0.88 | 0.125 | **0.61** | **0.000** | **0.67** | **0.000** | **0.60** | **0.000** |
| P46821 | Microtubule-associated protein 1B | 2 | 1.12 | 0.320 | 1.27 | 0.105 | 1.04 | 0.468 | 1.48 | 0.072 | 0.99 | 0.484 | 1.65 | 0.205 | 1.09 | 0.339 |
| P46940 | Ras GTPase-activating-like protein IQGAP1 | 2 | **1.68** | **0.019** | **1.58** | **0.017** | **1.95** | **0.002** | 1.55 | 0.073 | **1.64** | **0.000** | **1.79** | **0.008** | **1.48** | **0.015** |
| P47813 | Eukaryotic translation initiation factor 1A. X-chromosomal | 2 | 0.88 | 0.384 | 0.79 | 0.241 | 1.12 | 0.442 | 0.75 | 0.272 | 0.74 | 0.340 | 0.92 | 0.448 | 0.72 | 0.132 |
| P49585 | Choline-phosphate cytidylyltransferase A | 2 | 2.93 | 0.080 | 2.59 | 0.106 | 2.61 | 0.104 | 2.56 | 0.110 | **4.03** | **0.032** | 1.84 | 0.223 | 2.28 | 0.148 |
| P49748 | Very long-chain specific acyl-CoA dehydrogenase. mitochondrial | 2 | 1.07 | 0.445 | 0.87 | 0.384 | 0.88 | 0.389 | 0.81 | 0.333 | 0.81 | 0.331 | 0.71 | 0.243 | 0.84 | 0.364 |
| P49773 | Histidine triad nucleotide-binding protein 1 | 2 | **0.65** | **0.026** | 0.85 | 0.139 | **0.82** | **0.012** | 0.88 | 0.244 | 0.66 | 0.055 | **0.62** | **0.001** | 0.83 | 0.196 |
| P50238 | Cysteine-rich protein 1 | 2 | 0.86 | 0.326 | 0.79 | 0.219 | 0.89 | 0.347 | 1.14 | 0.340 | 0.94 | 0.415 | 0.67 | 0.100 | 0.75 | 0.175 |
| P50454 | Serpin H1 | 2 | 1.30 | 0.215 | 1.40 | 0.061 | **1.49** | **0.005** | **1.60** | **0.001** | **1.61** | **0.032** | **1.60** | **0.002** | 1.25 | 0.092 |
| P50552 | Vasodilator-stimulated phosphoprotein | 2 | **1.65** | **0.016** | **1.70** | **0.012** | 1.27 | 0.141 | 1.50 | 0.116 | **1.54** | **0.029** | 1.55 | 0.173 | 1.81 | 0.056 |
| P50991 | T-complex protein 1 subunit delta | 2 | 0.95 | 0.406 | 1.39 | 0.053 | 1.45 | 0.338 | 0.82 | 0.349 | 1.36 | 0.248 | 1.95 | 0.144 | 1.04 | 0.448 |
| P51659 | Peroxisomal multifunctional enzyme type 2 | 2 | 1.34 | 0.062 | 1.29 | 0.136 | 1.27 | 0.158 | 1.28 | 0.104 | 1.11 | 0.390 | 1.29 | 0.207 | 1.20 | 0.210 |
| P52292 | Importin subunit alpha-1 | 2 | 0.70 | 0.108 | **0.49** | **0.004** | 0.71 | 0.167 | 0.76 | 0.161 | 0.96 | 0.448 | **0.47** | **0.021** | 0.73 | 0.142 |
| P52701 | DNA mismatch repair protein Msh6 | 2 | 0.77 | 0.263 | 0.63 | 0.129 | 0.66 | 0.200 | 0.77 | 0.257 | 0.96 | 0.461 | 0.79 | 0.283 | 0.66 | 0.168 |
| P53618 | Coatomer subunit beta | 2 | 1.08 | 0.434 | 0.92 | 0.401 | 1.13 | 0.345 | 1.14 | 0.281 | 1.17 | 0.362 | 0.96 | 0.408 | 1.05 | 0.384 |
| P54819 | Adenylate kinase 2. mitochondrial | 2 | 1.31 | 0.311 | 1.26 | 0.332 | 1.36 | 0.279 | 1.06 | 0.457 | 1.27 | 0.335 | **2.47** | **0.037** | 1.51 | 0.223 |
| P54886 | Delta-1-pyrroline-5-carboxylate synthase | 2 | 0.84 | 0.323 | 1.10 | 0.377 | 1.01 | 0.495 | 0.96 | 0.455 | 0.95 | 0.449 | 0.98 | 0.479 | 1.14 | 0.383 |
| P55060 | Exportin-2 | 2 | 1.33 | 0.060 | **1.75** | **0.029** | 1.56 | 0.123 | 1.52 | 0.142 | **1.89** | **0.012** | **2.45** | **0.014** | 1.02 | 0.487 |
| P56192 | Methionine--tRNA ligase. cytoplasmic | 2 | 0.62 | 0.180 | 1.18 | 0.358 | 1.33 | 0.254 | 1.03 | 0.477 | 0.80 | 0.312 | 1.30 | 0.276 | 1.14 | 0.386 |
| P56545 | C-terminal-binding protein 2 | 2 | 0.94 | 0.445 | 1.08 | 0.437 | 1.11 | 0.423 | 1.33 | 0.255 | 0.81 | 0.324 | 0.92 | 0.422 | 0.93 | 0.432 |
| P60660 | Myosin light polypeptide 6 | 2 | 1.20 | 0.101 | 1.03 | 0.461 | **1.42** | **0.020** | 1.22 | 0.099 | 1.15 | 0.199 | 1.11 | 0.251 | **1.38** | **0.017** |
| P61224 | Ras-related protein Rap-1b | 2 | 1.07 | 0.440 | 0.91 | 0.336 | 1.17 | 0.254 | 1.00 | 0.499 | 1.08 | 0.411 | 0.88 | 0.242 | 1.26 | 0.228 |
| P61254 | 60S ribosomal protein L26 | 2 | 0.68 | 0.181 | 0.71 | 0.205 | 0.57 | 0.089 | 0.64 | 0.181 | 0.66 | 0.262 | 0.52 | 0.071 | **0.46** | **0.050** |
| P61604 | 10 kDa heat shock protein. mitochondrial | 2 | 1.03 | 0.449 | 0.99 | 0.487 | 0.91 | 0.353 | 0.87 | 0.213 | 0.73 | 0.069 | 0.96 | 0.429 | 0.90 | 0.345 |
| P62081 | 40S ribosomal protein S7 | 2 | 1.11 | 0.400 | **1.58** | **0.001** | 1.33 | 0.300 | 1.21 | 0.258 | **1.60** | **0.004** | 1.32 | 0.317 | 1.28 | 0.302 |
| P62266 | 40S ribosomal protein S23 | 2 | 0.84 | 0.236 | 0.98 | 0.447 | 0.82 | 0.137 | 1.02 | 0.472 | 0.77 | 0.226 | 0.74 | 0.125 | 0.92 | 0.371 |
| P62318 | Small nuclear ribonucleoprotein Sm D3 | 2 | 0.68 | 0.104 | 0.67 | 0.119 | **0.57** | **0.003** | 0.72 | 0.236 | 0.79 | 0.214 | **0.50** | **0.000** | 0.98 | 0.456 |
| P62333 | 26S protease regulatory subunit 10B | 2 | 0.52 | 0.080 | 0.69 | 0.227 | 0.84 | 0.203 | 0.78 | 0.170 | 0.63 | 0.116 | 0.74 | 0.197 | **0.58** | **0.036** |
| P62495 | Eukaryotic peptide chain release factor subunit 1 | 2 | **0.77** | **0.014** | 0.92 | 0.256 | 0.86 | 0.151 | 0.96 | 0.350 | 0.93 | 0.382 | 0.82 | 0.192 | **0.77** | **0.035** |
| P62750 | 60S ribosomal protein L23a | 2 | 0.67 | 0.126 | 1.00 | 0.497 | 0.75 | 0.210 | **0.53** | **0.037** | 0.76 | 0.217 | 0.61 | 0.076 | 0.78 | 0.237 |
| P62753 | 40S ribosomal protein S6 | 2 | 1.08 | 0.444 | 0.92 | 0.411 | 0.98 | 0.487 | 1.05 | 0.453 | 1.21 | 0.370 | 1.11 | 0.442 | 0.88 | 0.383 |
| P62829 | 60S ribosomal protein L23 | 2 | 0.84 | 0.375 | 0.77 | 0.341 | 0.99 | 0.493 | 0.98 | 0.490 | 0.67 | 0.310 | 0.63 | 0.288 | 1.07 | 0.453 |
| P62847 | 40S ribosomal protein S24 | 2 | 1.26 | 0.202 | 1.26 | 0.225 | 1.26 | 0.198 | 1.14 | 0.331 | 1.41 | 0.137 | 1.17 | 0.235 | **1.37** | **0.035** |
| P62888 | 60S ribosomal protein L30 | 2 | **2.05** | **0.047** | 1.29 | 0.295 | 2.72 | 0.079 | 0.83 | 0.342 | 1.36 | 0.247 | **3.00** | **0.004** | 1.74 | 0.199 |
| P62899 | 60S ribosomal protein L31 | 2 | 0.99 | 0.477 | 0.95 | 0.372 | 0.98 | 0.462 | 1.08 | 0.403 | 1.14 | 0.336 | 0.90 | 0.311 | 1.17 | 0.287 |
| P63010 | AP-2 complex subunit beta | 2 | 1.19 | 0.225 | 1.28 | 0.134 | 1.20 | 0.213 | 1.28 | 0.286 | 1.01 | 0.486 | 1.10 | 0.373 | 1.13 | 0.402 |
| P63220 | 40S ribosomal protein S21 | 2 | **3.29** | **0.000** | 1.29 | 0.102 | **4.06** | **0.000** | 1.22 | 0.239 | 1.01 | 0.480 | **3.28** | **0.000** | 1.38 | 0.108 |
| P67936 | Tropomyosin alpha-4 chain | 2 | **0.69** | **0.016** | 0.70 | 0.224 | **0.52** | **0.007** | **0.71** | **0.008** | **0.53** | **0.001** | 0.70 | 0.133 | **0.57** | **0.001** |
| Q00341 | Vigilin | 2 | 1.02 | 0.484 | 1.08 | 0.361 | 0.84 | 0.303 | 0.86 | 0.335 | 1.02 | 0.480 | 0.91 | 0.365 | 0.76 | 0.227 |
| Q01518 | Adenylyl cyclase-associated protein 1 | 2 | 0.95 | 0.451 | 0.95 | 0.452 | 1.17 | 0.367 | 0.98 | 0.475 | 1.11 | 0.416 | 0.87 | 0.383 | 1.17 | 0.372 |
| Q02218 | 2-oxoglutarate dehydrogenase. mitochondrial | 2 | **1.89** | **0.002** | 1.41 | 0.237 | 1.68 | 0.253 | 1.11 | 0.273 | 1.81 | 0.102 | 1.40 | 0.167 | 1.69 | 0.179 |
| Q02818 | Nucleobindin-1 | 2 | 0.77 | 0.302 | 0.87 | 0.412 | 0.81 | 0.170 | 0.95 | 0.459 | 0.91 | 0.439 | 0.90 | 0.415 | 0.87 | 0.406 |
| Q02878 | 60S ribosomal protein L6 | 2 | 0.73 | 0.053 | 0.81 | 0.064 | **0.82** | **0.009** | 0.89 | 0.066 | **0.78** | **0.004** | **0.72** | **0.019** | **0.78** | **0.027** |
| Q03135 | Caveolin-1 | 2 | 0.62 | 0.176 | 0.89 | 0.364 | 0.69 | 0.158 | 1.30 | 0.218 | 0.84 | 0.314 | 0.59 | 0.194 | 0.73 | 0.234 |
| Q04760 | Lactoylglutathione lyase | 2 | 0.94 | 0.329 | 1.04 | 0.437 | 1.13 | 0.210 | 1.07 | 0.412 | 0.99 | 0.475 | 0.91 | 0.322 | 1.05 | 0.352 |
| Q07020 | 60S ribosomal protein L18 | 2 | 0.87 | 0.133 | 1.04 | 0.442 | 0.84 | 0.159 | 1.15 | 0.173 | **0.63** | **0.025** | **0.80** | **0.044** | 0.97 | 0.401 |
| Q07955 | Serine/arginine-rich splicing factor 1 | 2 | 0.43 | 0.065 | 1.02 | 0.490 | 0.66 | 0.201 | 0.91 | 0.422 | 0.57 | 0.146 | 0.84 | 0.360 | 0.68 | 0.229 |
| Q08J23 | tRNA (cytosine(34)-C(5))-methyltransferase | 2 | 0.71 | 0.229 | 0.72 | 0.167 | 0.73 | 0.242 | 0.89 | 0.344 | 0.72 | 0.127 | 0.88 | 0.289 | 0.84 | 0.253 |
| Q09666 | Neuroblast differentiation-associated protein AHNAK | 2 | 0.99 | 0.493 | 0.97 | 0.481 | 0.58 | 0.243 | 1.47 | 0.290 | 1.21 | 0.398 | 1.51 | 0.280 | 0.74 | 0.352 |
| Q12860 | Contactin-1 | 2 | 0.99 | 0.474 | 1.09 | 0.271 | 1.08 | 0.333 | 1.11 | 0.394 | 1.13 | 0.428 | 1.27 | 0.079 | 1.06 | 0.366 |
| Q12906 | Interleukin enhancer-binding factor 3 | 2 | 0.84 | 0.325 | 0.89 | 0.373 | 0.83 | 0.311 | 0.68 | 0.215 | 0.67 | 0.159 | 0.79 | 0.271 | 0.60 | 0.109 |
| Q12965 | Unconventional myosin-Ie | 2 | 0.98 | 0.474 | 0.72 | 0.213 | 0.99 | 0.489 | 0.99 | 0.490 | 0.85 | 0.350 | 1.01 | 0.493 | 1.44 | 0.184 |
| Q13310 | Polyadenylate-binding protein 4 | 2 | 0.91 | 0.419 | 0.79 | 0.313 | 0.76 | 0.281 | 0.86 | 0.374 | 0.72 | 0.257 | 0.97 | 0.471 | 0.69 | 0.236 |
| Q14157 | Ubiquitin-associated protein 2-like | 2 | 0.69 | 0.250 | 0.51 | 0.100 | 0.40 | 0.113 | 0.51 | 0.117 | 0.39 | 0.095 | 0.62 | 0.181 | 0.40 | 0.062 |
| Q14651 | Plastin-1 | 2 | 1.24 | 0.197 | 1.10 | 0.441 | 1.17 | 0.407 | 1.10 | 0.431 | 1.00 | 0.496 | 0.92 | 0.442 | 1.30 | 0.313 |
| Q14847 | LIM and SH3 domain protein 1 | 2 | **0.53** | **0.002** | **0.60** | **0.000** | **0.47** | **0.001** | 0.69 | 0.091 | **0.48** | **0.000** | **0.55** | **0.000** | **0.50** | **0.000** |
| Q15056 | Eukaryotic translation initiation factor 4H | 2 | **0.58** | **0.034** | **0.63** | **0.025** | **0.50** | **0.027** | **0.64** | **0.016** | 0.46 | 0.101 | 0.58 | 0.090 | 0.64 | 0.187 |
| Q15102 | Platelet-activating factor acetylhydrolase IB subunit gamma | 2 | 1.68 | 0.119 | 1.20 | 0.333 | 1.36 | 0.221 | 1.10 | 0.420 | 1.53 | 0.092 | 1.01 | 0.489 | 1.65 | 0.072 |
| Q15165 | Serum paraoxonase/arylesterase 2 | 2 | 1.23 | 0.088 | **1.59** | **0.000** | 1.15 | 0.184 | 1.19 | 0.125 | **1.41** | **0.012** | 1.35 | 0.094 | 1.12 | 0.246 |
| Q15181 | Inorganic pyrophosphatase | 2 | 1.07 | 0.427 | 1.02 | 0.486 | 1.13 | 0.390 | 1.08 | 0.406 | 1.20 | 0.356 | 1.30 | 0.255 | 1.16 | 0.387 |
| Q15363 | Transmembrane emp24 domain-containing protein 2 | 2 | 1.33 | 0.255 | **1.48** | **0.049** | 1.25 | 0.303 | 1.01 | 0.499 | 1.05 | 0.466 | 1.12 | 0.409 | 1.42 | 0.106 |
| Q15365 | Poly(rC)-binding protein 1 | 2 | 0.85 | 0.269 | 1.13 | 0.260 | 0.92 | 0.310 | 1.18 | 0.231 | 0.97 | 0.459 | 0.95 | 0.415 | 0.93 | 0.394 |
| Q15366 | Poly(rC)-binding protein 2 | 2 | 1.09 | 0.411 | 1.08 | 0.419 | 1.24 | 0.245 | 1.16 | 0.128 | 1.04 | 0.448 | 1.28 | 0.109 | 1.04 | 0.457 |
| Q16531 | DNA damage-binding protein 1 | 2 | 1.03 | 0.443 | 1.20 | 0.260 | 0.94 | 0.383 | 1.38 | 0.071 | 1.36 | 0.181 | **1.33** | **0.013** | 1.14 | 0.236 |
| Q16658 | Fascin | 2 | 1.31 | 0.290 | 1.07 | 0.384 | 1.01 | 0.485 | 1.09 | 0.411 | 1.07 | 0.432 | 0.77 | 0.105 | 1.15 | 0.236 |
| Q16795 | NADH dehydrogenase [ubiquinone] 1 alpha subcomplex subunit 9. mitochondrial | 2 | 1.06 | 0.354 | **1.50** | **0.022** | 1.24 | 0.151 | 1.24 | 0.304 | **1.38** | **0.015** | 1.33 | 0.211 | 1.27 | 0.181 |
| Q63HN8 | E3 ubiquitin-protein ligase RNF213 | 2 | 0.81 | 0.212 | 0.91 | 0.258 | 0.82 | 0.215 | 0.98 | 0.455 | **0.66** | **0.010** | 1.08 | 0.386 | **0.77** | **0.045** |
| Q6DD88 | Atlastin-3 | 2 | 1.58 | 0.071 | **2.12** | **0.014** | **1.54** | **0.018** | **1.76** | **0.016** | **2.27** | **0.000** | **2.55** | **0.049** | 1.38 | 0.267 |
| Q86VP6 | Cullin-associated NEDD8-dissociated protein 1 | 2 | 1.16 | 0.365 | 1.14 | 0.383 | 1.15 | 0.393 | 0.94 | 0.455 | 1.08 | 0.429 | 1.28 | 0.370 | 1.03 | 0.477 |
| Q8NBS9 | Thioredoxin domain-containing protein 5 | 2 | 0.99 | 0.442 | 1.00 | 0.489 | **1.28** | **0.010** | 0.97 | 0.430 | 1.00 | 0.492 | 1.20 | 0.163 | 1.12 | 0.346 |
| Q8NE71 | ATP-binding cassette sub-family F member 1 | 2 | 1.20 | 0.403 | 1.10 | 0.450 | 1.24 | 0.379 | 1.10 | 0.395 | 1.13 | 0.417 | 1.17 | 0.415 | 1.01 | 0.499 |
| Q8NHW5 | 60S acidic ribosomal protein P0-like | 2 | 1.17 | 0.171 | 1.07 | 0.384 | 1.10 | 0.307 | 1.10 | 0.335 | 1.20 | 0.151 | 1.04 | 0.416 | 1.12 | 0.296 |
| Q8WUM4 | Programmed cell death 6-interacting protein | 2 | 0.72 | 0.241 | 0.75 | 0.269 | 1.00 | 0.499 | 0.88 | 0.381 | 1.43 | 0.210 | 0.71 | 0.230 | 0.77 | 0.308 |
| Q92538 | Golgi-specific brefeldin A-resistance guanine nucleotide exchange factor 1 | 2 | **2.11** | **0.000** | 1.44 | 0.113 | **2.70** | **0.000** | 1.02 | 0.489 | **1.85** | **0.008** | 1.60 | 0.184 | 1.71 | 0.152 |
| Q92841 | Probable ATP-dependent RNA helicase DDX17 | 2 | 1.04 | 0.478 | 1.08 | 0.446 | 0.79 | 0.339 | 0.97 | 0.479 | 0.58 | 0.198 | 1.01 | 0.495 | 0.45 | 0.128 |
| Q92945 | Far upstream element-binding protein 2 | 2 | **0.74** | **0.007** | **0.70** | **0.007** | 0.76 | 0.073 | 1.08 | 0.398 | 0.90 | 0.358 | 0.89 | 0.376 | 0.66 | 0.109 |
| Q93009 | Ubiquitin carboxyl-terminal hydrolase 7 | 2 | 0.65 | 0.176 | 1.05 | 0.461 | 0.73 | 0.236 | 0.90 | 0.403 | 0.84 | 0.347 | 1.34 | 0.244 | 0.88 | 0.400 |
| Q96AG4 | Leucine-rich repeat-containing protein 59 | 2 | **1.48** | **0.026** | 1.22 | 0.199 | 1.23 | 0.106 | 1.26 | 0.144 | **1.46** | **0.029** | 1.00 | 0.494 | **1.37** | **0.021** |
| Q99436 | Proteasome subunit beta type-7 | 2 | 0.80 | 0.141 | 1.05 | 0.457 | 0.94 | 0.342 | 1.04 | 0.359 | 1.06 | 0.427 | 0.85 | 0.282 | 1.18 | 0.354 |
| Q99729 | Heterogeneous nuclear ribonucleoprotein A/B | 2 | 1.11 | 0.434 | 1.22 | 0.364 | 1.08 | 0.460 | 1.08 | 0.427 | 1.19 | 0.416 | 1.17 | 0.406 | 1.07 | 0.461 |
| Q99829 | Copine-1 | 2 | 1.05 | 0.460 | 0.93 | 0.358 | 0.88 | 0.386 | 0.94 | 0.463 | 1.13 | 0.373 | 0.72 | 0.066 | 0.92 | 0.434 |
| Q9BXJ9 | N-alpha-acetyltransferase 15. NatA auxiliary subunit | 2 | 1.96 | 0.052 | **1.64** | **0.000** | **2.01** | **0.022** | 1.36 | 0.269 | 1.51 | 0.117 | **2.07** | **0.042** | 1.50 | 0.128 |
| Q9H583 | HEAT repeat-containing protein 1 | 2 | 0.69 | 0.068 | 0.87 | 0.380 | 0.77 | 0.091 | 0.98 | 0.460 | 0.89 | 0.351 | 0.84 | 0.371 | 1.16 | 0.253 |
| Q9H773 | dCTP pyrophosphatase 1 | 2 | 0.96 | 0.461 | 1.00 | 0.492 | 0.95 | 0.445 | 0.89 | 0.383 | 0.91 | 0.408 | 1.10 | 0.409 | 0.86 | 0.359 |
| Q9NR28 | Diablo homolog. mitochondrial | 2 | 1.12 | 0.359 | 1.04 | 0.413 | 0.92 | 0.416 | **0.67** | **0.011** | 1.20 | 0.268 | 0.84 | 0.167 | 0.84 | 0.296 |
| Q9NR30 | Nucleolar RNA helicase 2 | 2 | 0.83 | 0.125 | 0.93 | 0.354 | **0.63** | **0.002** | 0.96 | 0.402 | 0.87 | 0.210 | 0.86 | 0.345 | 0.71 | 0.083 |
| Q9NS69 | Mitochondrial import receptor subunit TOM22 homolog | 2 | 0.86 | 0.193 | 0.79 | 0.152 | 0.77 | 0.068 | **0.52** | **0.000** | 0.83 | 0.298 | **0.67** | **0.022** | 0.73 | 0.113 |
| Q9NZN3 | EH domain-containing protein 3 | 2 | 1.93 | 0.206 | 0.99 | 0.486 | 1.24 | 0.331 | 1.21 | 0.351 | 1.90 | 0.164 | 1.60 | 0.167 | 1.69 | 0.140 |
| Q9NZN4 | EH domain-containing protein 2 | 2 | 0.79 | 0.364 | 0.67 | 0.167 | 0.66 | 0.245 | 1.55 | 0.055 | 0.98 | 0.475 | 0.83 | 0.243 | 0.73 | 0.140 |
| Q9P2J5 | Leucine--tRNA ligase. cytoplasmic | 2 | 1.39 | 0.207 | 1.12 | 0.394 | 1.13 | 0.386 | 1.12 | 0.389 | 1.27 | 0.278 | **2.01** | **0.036** | 1.16 | 0.361 |
| Q9UHD8 | Septin-9 | 2 | 0.84 | 0.300 | 0.98 | 0.472 | 0.97 | 0.442 | 1.11 | 0.215 | 1.06 | 0.391 | 1.03 | 0.451 | 1.04 | 0.407 |
| Q9UHG3 | Prenylcysteine oxidase 1 | 2 | 1.33 | 0.187 | 0.88 | 0.320 | 1.01 | 0.489 | 0.92 | 0.415 | 1.18 | 0.283 | 1.24 | 0.226 | 1.17 | 0.157 |
| Q9UKK9 | ADP-sugar pyrophosphatase | 2 | 0.74 | 0.264 | 1.05 | 0.458 | 1.13 | 0.398 | 0.76 | 0.285 | 0.87 | 0.382 | 1.36 | 0.260 | 1.10 | 0.423 |
| Q9ULV4 | Coronin-1C | 2 | 1.50 | 0.093 | 1.23 | 0.246 | 1.48 | 0.107 | 0.84 | 0.141 | **1.80** | **0.036** | 1.23 | 0.290 | 1.24 | 0.250 |
| Q9UQ80 | Proliferation-associated protein 2G4 | 2 | 1.22 | 0.357 | 1.22 | 0.351 | 1.18 | 0.378 | 1.31 | 0.304 | 1.46 | 0.234 | 1.54 | 0.201 | 1.24 | 0.342 |
| Q9Y490 | Talin-1 | 2 | **1.75** | **0.006** | 1.22 | 0.398 | 1.30 | 0.135 | 1.17 | 0.274 | 1.11 | 0.437 | 1.35 | 0.186 | 1.21 | 0.188 |
| Q9Y4L1 | Hypoxia up-regulated protein 1 | 2 | 1.06 | 0.437 | 0.78 | 0.184 | 1.36 | 0.306 | 0.72 | 0.315 | 0.83 | 0.396 | 1.01 | 0.493 | 0.86 | 0.389 |
| A0AV96 | RNA-binding protein 47 | 1 | 0.77 | 0.270 | 0.84 | 0.419 | 0.78 | 0.279 | 1.15 | 0.416 | 0.66 | 0.229 | 0.81 | 0.366 | 0.68 | 0.191 |
| A0AVT1 | Ubiquitin-like modifier-activating enzyme 6 | 1 | 1.17 | 0.340 | 1.25 | 0.273 | 1.22 | 0.299 | 1.28 | 0.253 | 1.35 | 0.215 | 1.54 | 0.120 | 1.24 | 0.284 |
| A8MXV4 | Nucleoside diphosphate-linked moiety X motif 19. mitochondrial | 1 | 1.06 | 0.459 | 1.04 | 0.471 | 1.39 | 0.250 | 1.33 | 0.284 | 1.43 | 0.236 | 1.55 | 0.188 | 0.99 | 0.487 |
| E9PAV3 | Nascent polypeptide-associated complex subunit alpha. muscle-specific form | 1 | **1.43** | **0.014** | **1.51** | **0.008** | 1.28 | 0.208 | **1.53** | **0.029** | 1.60 | 0.059 | 1.28 | 0.298 | 1.45 | 0.084 |
| O00203 | AP-3 complex subunit beta-1 | 1 | 1.78 | 0.227 | 1.21 | 0.387 | 0.64 | 0.246 | 0.88 | 0.416 | 0.85 | 0.423 | 1.14 | 0.418 | 1.18 | 0.422 |
| O00231 | 26S proteasome non-ATPase regulatory subunit 11 | 1 | 0.87 | 0.249 | 0.80 | 0.076 | **0.76** | **0.038** | 0.84 | 0.248 | **0.55** | **0.000** | **0.71** | **0.018** | 0.73 | 0.250 |
| O00232 | 26S proteasome non-ATPase regulatory subunit 12 | 1 | 1.14 | 0.396 | 0.88 | 0.395 | 0.90 | 0.412 | 1.01 | 0.499 | 0.60 | 0.163 | 1.28 | 0.307 | 0.91 | 0.419 |
| O00264 | Membrane-associated progesterone receptor component 1 | 1 | 1.18 | 0.095 | 0.88 | 0.238 | 1.37 | 0.193 | 1.00 | 0.490 | 1.17 | 0.294 | 0.99 | 0.470 | 1.30 | 0.158 |
| O00299 | Chloride intracellular channel protein 1 | 1 | 0.62 | 0.133 | 0.69 | 0.078 | **0.58** | **0.001** | 0.82 | 0.357 | 0.63 | 0.192 | 0.71 | 0.199 | **0.58** | **0.035** |
| O00410 | Importin-5 | 1 | 0.53 | 0.291 | 0.75 | 0.280 | 0.66 | 0.223 | 0.50 | 0.086 | 0.66 | 0.234 | 0.74 | 0.264 | 0.63 | 0.268 |
| O00411 | DNA-directed RNA polymerase. mitochondrial | 1 | 0.71 | 0.098 | 0.60 | 0.063 | **0.65** | **0.026** | **0.72** | **0.003** | 0.80 | 0.061 | **0.61** | **0.009** | **0.70** | **0.003** |
| O00442 | RNA 3'-terminal phosphate cyclase | 1 | 1.01 | 0.499 | 0.57 | 0.238 | 1.26 | 0.368 | 0.76 | 0.354 | 1.08 | 0.462 | 0.64 | 0.286 | 2.07 | 0.143 |
| O00625 | Pirin | 1 | 0.64 | 0.159 | 0.67 | 0.176 | 0.58 | 0.107 | 0.85 | 0.346 | 1.05 | 0.461 | **0.44** | **0.036** | 0.59 | 0.124 |
| O00629 | Importin subunit alpha-3 | 1 | 0.56 | 0.159 | 1.11 | 0.422 | 0.61 | 0.180 | 0.54 | 0.136 | 0.45 | 0.098 | 0.59 | 0.180 | 0.89 | 0.410 |
| O14617 | AP-3 complex subunit delta-1 | 1 | **3.59** | **0.047** | 2.46 | 0.122 | **4.81** | **0.018** | 2.40 | 0.129 | **4.75** | **0.020** | 2.47 | 0.124 | **4.21** | **0.030** |
| O14828 | Secretory carrier-associated membrane protein 3 | 1 | 0.65 | 0.201 | 0.66 | 0.197 | 1.12 | 0.410 | 0.85 | 0.370 | 0.85 | 0.368 | 0.75 | 0.284 | 0.84 | 0.364 |
| O14979 | Heterogeneous nuclear ribonucleoprotein D-like | 1 | 0.70 | 0.143 | 0.86 | 0.202 | 0.79 | 0.170 | 1.02 | 0.467 | 0.86 | 0.121 | **0.75** | **0.012** | 0.95 | 0.358 |
| O15145 | Actin-related protein 2/3 complex subunit 3 | 1 | 0.86 | 0.359 | 0.69 | 0.183 | 0.88 | 0.373 | 0.72 | 0.216 | 0.88 | 0.374 | 1.03 | 0.477 | 0.98 | 0.483 |
| O15173 | Membrane-associated progesterone receptor component 2 | 1 | 1.04 | 0.464 | 1.05 | 0.451 | 1.26 | 0.246 | 1.08 | 0.425 | 1.06 | 0.434 | 1.20 | 0.300 | 1.13 | 0.361 |
| O15294 | UDP-N-acetylglucosamine--peptide N-acetylglucosaminyltransferase 110 kDa subunit | 1 | 1.51 | 0.166 | 1.12 | 0.401 | 0.97 | 0.472 | **1.98** | **0.050** | 1.30 | 0.287 | 0.86 | 0.362 | 0.98 | 0.478 |
| O43175 | D-3-phosphoglycerate dehydrogenase | 1 | 1.09 | 0.410 | 1.38 | 0.052 | 1.22 | 0.305 | 1.29 | 0.111 | 1.20 | 0.354 | 1.18 | 0.201 | **1.47** | **0.025** |
| O43324 | Eukaryotic translation elongation factor 1 epsilon-1 | 1 | 0.91 | 0.414 | 1.01 | 0.481 | 1.00 | 0.499 | 1.20 | 0.175 | 0.96 | 0.459 | 1.32 | 0.136 | 0.97 | 0.469 |
| O43488 | Aflatoxin B1 aldehyde reductase member 2 | 1 | **1.39** | **0.035** | 1.30 | 0.230 | 1.60 | 0.168 | 1.14 | 0.317 | 1.39 | 0.161 | 1.11 | 0.421 | 1.49 | 0.084 |
| O43837 | Isocitrate dehydrogenase [NAD] subunit beta. mitochondrial | 1 | 1.78 | 0.141 | 1.88 | 0.115 | 1.37 | 0.279 | 1.69 | 0.161 | 1.96 | 0.104 | 1.37 | 0.287 | 1.84 | 0.129 |
| O43852 | Calumenin | 1 | 1.07 | 0.440 | 1.28 | 0.265 | 1.36 | 0.226 | 0.78 | 0.302 | 1.33 | 0.235 | 1.16 | 0.361 | 0.92 | 0.413 |
| O60216 | Double-strand-break repair protein rad21 homolog | 1 | 0.54 | 0.130 | 1.30 | 0.297 | 0.86 | 0.380 | 0.94 | 0.444 | 0.53 | 0.126 | 0.67 | 0.227 | 0.56 | 0.149 |
| O60271 | C-Jun-amino-terminal kinase-interacting protein 4 | 1 | 1.03 | 0.477 | 0.66 | 0.191 | 0.68 | 0.206 | 0.91 | 0.414 | 0.59 | 0.141 | 0.67 | 0.203 | 0.70 | 0.232 |
| O60443 | Non-syndromic hearing impairment protein 5 | 1 | 0.84 | 0.362 | 0.58 | 0.139 | 0.85 | 0.362 | 1.09 | 0.433 | 1.13 | 0.405 | 0.91 | 0.421 | 0.77 | 0.300 |
| O60493 | Sorting nexin-3 | 1 | 0.47 | 0.088 | 0.68 | 0.223 | 0.47 | 0.077 | 0.93 | 0.438 | 0.38 | 0.052 | 0.86 | 0.379 | 0.61 | 0.183 |
| O60716 | Catenin delta-1 | 1 | 1.19 | 0.319 | 1.28 | 0.249 | 1.04 | 0.466 | **1.56** | **0.034** | 1.19 | 0.301 | 1.23 | 0.085 | 1.24 | 0.272 |
| O60888 | Protein CutA | 1 | 1.36 | 0.355 | 1.15 | 0.435 | 1.45 | 0.320 | 1.47 | 0.314 | 1.35 | 0.361 | 0.84 | 0.420 | 1.45 | 0.326 |
| O60936 | Nucleolar protein 3 | 1 | 1.26 | 0.333 | 1.87 | 0.110 | 1.41 | 0.253 | **2.34** | **0.046** | 1.82 | 0.124 | 1.35 | 0.284 | 1.30 | 0.316 |
| O75116 | Rho-associated protein kinase 2 | 1 | 1.07 | 0.466 | 1.41 | 0.240 | 0.72 | 0.386 | 0.97 | 0.475 | 1.09 | 0.437 | 1.05 | 0.467 | 1.25 | 0.384 |
| O75165 | DnaJ homolog subfamily C member 13 | 1 | 1.26 | 0.359 | 1.50 | 0.156 | 1.01 | 0.497 | 1.44 | 0.216 | **1.67** | **0.024** | 1.68 | 0.186 | 1.50 | 0.239 |
| O75208 | Ubiquinone biosynthesis protein COQ9. mitochondrial | 1 | 1.27 | 0.146 | 0.97 | 0.438 | 1.36 | 0.077 | 1.16 | 0.262 | **1.48** | **0.038** | 1.31 | 0.115 | **1.52** | **0.029** |
| O75340 | Programmed cell death protein 6 | 1 | 0.98 | 0.481 | 0.90 | 0.404 | 1.29 | 0.385 | 1.05 | 0.468 | 1.25 | 0.361 | 1.09 | 0.423 | 0.71 | 0.332 |
| O75347 | Tubulin-specific chaperone A | 1 | 0.93 | 0.453 | 1.11 | 0.433 | 0.68 | 0.261 | 0.91 | 0.434 | 0.70 | 0.282 | 1.51 | 0.237 | 1.03 | 0.482 |
| O75367 | Core histone macro-H2A.1 | 1 | 0.60 | 0.171 | 1.08 | 0.440 | 0.74 | 0.277 | 0.60 | 0.165 | 0.61 | 0.183 | 0.76 | 0.297 | 0.99 | 0.490 |
| O75569 | Interferon-inducible double-stranded RNA-dependent protein kinase activator A | 1 | 0.72 | 0.061 | **0.46** | **0.036** | 0.91 | 0.307 | 0.89 | 0.278 | 0.69 | 0.157 | 0.90 | 0.305 | 1.09 | 0.341 |
| O75818 | Ribonuclease P protein subunit p40 | 1 | **2.66** | **0.043** | 1.05 | 0.469 | 2.27 | 0.074 | 1.31 | 0.325 | 1.52 | 0.242 | **2.63** | **0.045** | 1.20 | 0.388 |
| O75964 | ATP synthase subunit g. mitochondrial | 1 | 1.65 | 0.112 | 1.42 | 0.108 | **1.69** | **0.004** | 1.37 | 0.075 | **1.66** | **0.030** | **1.56** | **0.030** | **1.64** | **0.027** |
| O76021 | Ribosomal L1 domain-containing protein 1 | 1 | 0.90 | 0.421 | 0.87 | 0.393 | 0.67 | 0.236 | 0.56 | 0.160 | 0.71 | 0.274 | 0.79 | 0.334 | 0.87 | 0.403 |
| O95340 | Bifunctional 3'-phosphoadenosine 5'-phosphosulfate synthase 2 | 1 | 1.34 | 0.322 | 1.09 | 0.449 | 1.35 | 0.311 | 1.55 | 0.236 | 1.05 | 0.474 | 0.72 | 0.313 | 0.97 | 0.481 |
| O95373 | Importin-7 | 1 | 1.37 | 0.310 | 1.61 | 0.255 | 1.22 | 0.358 | 1.30 | 0.339 | **1.64** | **0.027** | 1.32 | 0.362 | 1.17 | 0.423 |
| O95456 | Proteasome assembly chaperone 1 | 1 | 1.60 | 0.230 | 1.95 | 0.141 | 2.15 | 0.105 | **3.13** | **0.029** | 2.26 | 0.095 | **4.50** | **0.006** | 2.43 | 0.077 |
| O95563 | Mitochondrial pyruvate carrier 2 | 1 | 2.10 | 0.110 | 0.76 | 0.334 | 1.99 | 0.125 | 0.78 | 0.352 | 2.10 | 0.111 | 1.02 | 0.491 | 1.71 | 0.195 |
| O95864 | Fatty acid desaturase 2 | 1 | 1.22 | 0.331 | 0.98 | 0.484 | 0.56 | 0.289 | 1.45 | 0.317 | 0.80 | 0.334 | 0.78 | 0.300 | 0.67 | 0.303 |
| O95881 | Thioredoxin domain-containing protein 12 | 1 | 1.33 | 0.364 | 1.88 | 0.203 | 2.27 | 0.137 | 1.29 | 0.375 | 2.32 | 0.136 | 1.05 | 0.478 | 2.51 | 0.114 |
| O96013 | Serine/threonine-protein kinase PAK 4 | 1 | 0.62 | 0.200 | 0.86 | 0.388 | 0.68 | 0.236 | 1.06 | 0.462 | **0.26** | **0.033** | 0.38 | 0.065 | 0.48 | 0.119 |
| P00367 | Glutamate dehydrogenase 1. mitochondrial | 1 | 1.22 | 0.341 | 1.14 | 0.391 | 0.91 | 0.418 | 1.39 | 0.243 | 1.37 | 0.258 | 0.99 | 0.486 | 1.26 | 0.315 |
| P00390 | Glutathione reductase. mitochondrial | 1 | 0.74 | 0.175 | 0.82 | 0.265 | 0.80 | 0.241 | 0.76 | 0.199 | 0.82 | 0.267 | 0.76 | 0.198 | 0.88 | 0.339 |
| P00492 | Hypoxanthine-guanine phosphoribosyltransferase | 1 | 0.89 | 0.414 | 1.09 | 0.357 | 1.26 | 0.162 | 1.26 | 0.142 | 1.22 | 0.412 | 1.28 | 0.129 | 1.10 | 0.422 |
| P00533 | Epidermal growth factor receptor | 1 | 1.18 | 0.364 | 1.54 | 0.071 | 1.27 | 0.102 | 1.54 | 0.152 | 0.84 | 0.381 | 1.15 | 0.419 | 0.95 | 0.399 |
| P00568 | Adenylate kinase isoenzyme 1 | 1 | 1.21 | 0.357 | 0.93 | 0.396 | 1.80 | 0.113 | 1.22 | 0.222 | 1.99 | 0.101 | 1.25 | 0.306 | **1.92** | **0.003** |
| P02753 | Retinol-binding protein 4 | 1 | 1.22 | 0.354 | 1.86 | 0.104 | 1.48 | 0.216 | 1.74 | 0.131 | 2.22 | 0.053 | 2.02 | 0.077 | 1.19 | 0.369 |
| P02792 | Ferritin light chain | 1 | 1.67 | 0.129 | 1.11 | 0.415 | 1.02 | 0.490 | 1.02 | 0.489 | 0.39 | 0.095 | 1.16 | 0.381 | 0.56 | 0.141 |
| P04004 | Vitronectin | 1 | 1.23 | 0.401 | 1.29 | 0.339 | 1.16 | 0.447 | 1.51 | 0.220 | 1.46 | 0.325 | 1.51 | 0.337 | 1.14 | 0.408 |
| P04080 | Cystatin-B | 1 | 0.92 | 0.423 | 1.15 | 0.386 | 1.07 | 0.440 | 0.97 | 0.470 | 1.10 | 0.424 | 1.07 | 0.449 | 1.20 | 0.348 |
| P04179 | Superoxide dismutase [Mn]. mitochondrial | 1 | 4.57 | 0.055 | 2.75 | 0.148 | 4.09 | 0.068 | 2.87 | 0.137 | 4.71 | 0.052 | 3.74 | 0.085 | 4.30 | 0.064 |
| P05026 | Sodium/potassium-transporting ATPase subunit beta-1 | 1 | 1.97 | 0.135 | 1.26 | 0.357 | 1.24 | 0.367 | 1.41 | 0.292 | 1.34 | 0.325 | 2.06 | 0.119 | 1.74 | 0.190 |
| P05091 | Aldehyde dehydrogenase. mitochondrial | 1 | 1.08 | 0.414 | 0.91 | 0.410 | 0.89 | 0.371 | 0.95 | 0.369 | 1.09 | 0.457 | 1.11 | 0.274 | 1.10 | 0.425 |
| P05386 | 60S acidic ribosomal protein P1 | 1 | 1.32 | 0.201 | 0.99 | 0.487 | **1.45** | **0.002** | 0.89 | 0.300 | 0.93 | 0.436 | 1.56 | 0.173 | 0.94 | 0.329 |
| P05387 | 60S acidic ribosomal protein P2 | 1 | **0.35** | **0.039** | 0.62 | 0.175 | 0.44 | 0.063 | 0.80 | 0.327 | 0.90 | 0.417 | 0.80 | 0.328 | 0.71 | 0.259 |
| P06493 | Cyclin-dependent kinase 1 | 1 | 1.38 | 0.293 | 1.79 | 0.151 | 1.20 | 0.375 | 0.77 | 0.335 | 2.22 | 0.078 | 2.30 | 0.068 | 1.05 | 0.471 |
| P06703 | Protein S100-A6 | 1 | 0.73 | 0.162 | 0.81 | 0.247 | 0.96 | 0.444 | 1.29 | 0.216 | 1.01 | 0.494 | **0.41** | **0.022** | 0.82 | 0.263 |
| P07384 | Calpain-1 catalytic subunit | 1 | 0.34 | 0.117 | 2.18 | 0.106 | 1.81 | 0.174 | 1.71 | 0.201 | 2.57 | 0.067 | 1.12 | 0.435 | 1.46 | 0.287 |
| P07814 | Bifunctional glutamate/proline--tRNA ligase | 1 | 1.07 | 0.463 | 1.26 | 0.356 | 0.86 | 0.406 | 1.11 | 0.438 | 1.13 | 0.427 | 1.18 | 0.400 | 1.84 | 0.165 |
| P07858 | Cathepsin B | 1 | 1.45 | 0.253 | 1.84 | 0.130 | 1.92 | 0.113 | 1.89 | 0.121 | 1.94 | 0.114 | 1.49 | 0.236 | 1.48 | 0.243 |
| P07954 | Fumarate hydratase. mitochondrial | 1 | 1.16 | 0.325 | 1.13 | 0.337 | 1.23 | 0.136 | 0.79 | 0.350 | 1.17 | 0.359 | 1.04 | 0.420 | 1.25 | 0.187 |
| P08195 | 4F2 cell-surface antigen heavy chain | 1 | 0.88 | 0.445 | 0.97 | 0.464 | 0.72 | 0.220 | 1.09 | 0.418 | 0.72 | 0.230 | 0.75 | 0.249 | 1.01 | 0.494 |
| P08243 | Asparagine synthetase [glutamine-hydrolyzing] | 1 | 0.96 | 0.412 | 0.97 | 0.444 | 1.03 | 0.424 | 0.85 | 0.340 | 0.95 | 0.438 | 0.97 | 0.462 | 0.92 | 0.389 |
| P08397 | Porphobilinogen deaminase | 1 | 0.82 | 0.342 | 0.67 | 0.211 | 0.54 | 0.114 | 0.91 | 0.419 | 0.95 | 0.457 | 1.03 | 0.480 | 0.58 | 0.153 |
| P08697 | Alpha-2-antiplasmin | 1 | 1.45 | 0.275 | 0.52 | 0.170 | 2.32 | 0.074 | 0.32 | 0.079 | 0.55 | 0.205 | 1.91 | 0.141 | 0.57 | 0.221 |
| P09661 | U2 small nuclear ribonucleoprotein A' | 1 | 1.28 | 0.294 | 1.20 | 0.348 | 1.20 | 0.343 | 1.47 | 0.199 | 1.13 | 0.396 | 1.19 | 0.354 | 1.10 | 0.420 |
| P09669 | Cytochrome c oxidase subunit 6C | 1 | 0.86 | 0.374 | 0.93 | 0.435 | 1.09 | 0.428 | 0.80 | 0.320 | 1.01 | 0.495 | 0.83 | 0.348 | 1.03 | 0.478 |
| P09874 | Poly [ADP-ribose] polymerase 1 | 1 | 1.00 | 0.497 | 0.90 | 0.320 | 0.95 | 0.359 | 0.95 | 0.363 | **0.72** | **0.028** | 0.89 | 0.201 | 1.14 | 0.230 |
| P09936 | Ubiquitin carboxyl-terminal hydrolase isozyme L1 | 1 | **4.13** | **0.000** | **1.62** | **0.011** | **5.36** | **0.000** | 0.86 | 0.257 | 1.46 | 0.139 | **4.76** | **0.000** | 1.57 | 0.114 |
| P0C0L5 | Complement C4-B | 1 | 2.02 | 0.074 | 1.62 | 0.160 | 1.29 | 0.301 | 1.74 | 0.128 | 1.53 | 0.197 | 1.61 | 0.167 | 2.14 | 0.059 |
| P10155 | 60 kDa SS-A/Ro ribonucleoprotein | 1 | 1.13 | 0.398 | 1.07 | 0.420 | 1.20 | 0.326 | 0.89 | 0.384 | 1.03 | 0.474 | 1.02 | 0.484 | 1.19 | 0.374 |
| P10253 | Lysosomal alpha-glucosidase | 1 | 0.98 | 0.481 | 1.36 | 0.255 | 1.10 | 0.425 | 0.81 | 0.329 | 1.41 | 0.236 | 1.07 | 0.447 | 0.91 | 0.419 |
| P11177 | Pyruvate dehydrogenase E1 component subunit beta. mitochondrial | 1 | 1.17 | 0.299 | 1.26 | 0.097 | 0.91 | 0.355 | 1.29 | 0.162 | 1.43 | 0.244 | 1.03 | 0.481 | **1.47** | **0.030** |
| P11234 | Ras-related protein Ral-B | 1 | **2.61** | **0.050** | 2.25 | 0.081 | 1.92 | 0.132 | 1.56 | 0.230 | 2.40 | 0.068 | 1.38 | 0.303 | 2.02 | 0.120 |
| P11717 | Cation-independent mannose-6-phosphate receptor | 1 | 0.69 | 0.255 | 0.66 | 0.225 | 0.69 | 0.249 | 1.14 | 0.406 | 0.56 | 0.162 | 0.76 | 0.311 | 0.90 | 0.423 |
| P12074 | Cytochrome c oxidase subunit 6A1. mitochondrial | 1 | 2.49 | 0.119 | 3.13 | 0.065 | 1.94 | 0.195 | 3.35 | 0.054 | **3.53** | **0.048** | 2.46 | 0.121 | **4.13** | **0.030** |
| P12429 | Annexin A3 | 1 | 0.97 | 0.455 | 0.99 | 0.488 | 1.10 | 0.326 | 1.13 | 0.331 | 0.98 | 0.479 | 0.97 | 0.404 | 0.95 | 0.437 |
| P13073 | Cytochrome c oxidase subunit 4 isoform 1. mitochondrial | 1 | 1.11 | 0.313 | **0.79** | **0.049** | 1.03 | 0.446 | 1.07 | 0.326 | 0.81 | 0.178 | 0.71 | 0.161 | 1.04 | 0.410 |
| P13804 | Electron transfer flavoprotein subunit alpha. mitochondrial | 1 | **2.05** | **0.000** | 1.59 | 0.136 | **1.95** | **0.000** | 1.32 | 0.248 | 1.16 | 0.388 | 1.46 | 0.151 | **1.42** | **0.043** |
| P13987 | CD59 glycoprotein | 1 | 1.22 | 0.337 | 0.83 | 0.339 | 1.22 | 0.333 | 0.76 | 0.272 | 0.77 | 0.287 | 0.86 | 0.374 | 1.44 | 0.213 |
| P14735 | Insulin-degrading enzyme | 1 | 1.06 | 0.454 | 1.10 | 0.420 | 0.79 | 0.302 | 0.80 | 0.313 | 0.71 | 0.241 | 0.96 | 0.462 | 0.79 | 0.310 |
| P14868 | Aspartate--tRNA ligase. cytoplasmic | 1 | 0.59 | 0.173 | 0.77 | 0.305 | 0.38 | 0.050 | 0.56 | 0.151 | 0.56 | 0.160 | 0.97 | 0.474 | 0.55 | 0.158 |
| P14923 | Junction plakoglobin | 1 | 1.36 | 0.270 | 1.20 | 0.356 | 0.80 | 0.325 | 1.06 | 0.454 | 0.63 | 0.194 | 0.91 | 0.423 | 0.63 | 0.198 |
| P15880 | 40S ribosomal protein S2 | 1 | 1.50 | 0.257 | 1.22 | 0.376 | 1.71 | 0.185 | 1.51 | 0.249 | 0.90 | 0.437 | 0.69 | 0.291 | 1.55 | 0.243 |
| P16615 | Sarcoplasmic/endoplasmic reticulum calcium ATPase 2 | 1 | 0.82 | 0.205 | 1.09 | 0.295 | 0.78 | 0.217 | 0.75 | 0.155 | 0.80 | 0.271 | 0.88 | 0.331 | 0.86 | 0.331 |
| P16989 | Y-box-binding protein 3 | 1 | 0.51 | 0.102 | 0.58 | 0.142 | **0.37** | **0.030** | 0.60 | 0.153 | **0.38** | **0.044** | 0.55 | 0.127 | 0.47 | 0.084 |
| P17655 | Calpain-2 catalytic subunit | 1 | 1.51 | 0.231 | 1.80 | 0.096 | **2.13** | **0.032** | **2.32** | **0.002** | 2.38 | 0.070 | **1.88** | **0.019** | **2.09** | **0.030** |
| P17931 | Galectin-3 | 1 | 0.62 | 0.211 | 0.57 | 0.173 | 1.02 | 0.487 | 0.66 | 0.241 | 1.14 | 0.410 | 0.66 | 0.239 | **0.27** | **0.049** |
| P18858 | DNA ligase 1 | 1 | 1.13 | 0.413 | 0.72 | 0.275 | 1.08 | 0.444 | 1.38 | 0.270 | 0.97 | 0.475 | 0.96 | 0.464 | 0.69 | 0.260 |
| P19404 | NADH dehydrogenase [ubiquinone] flavoprotein 2. mitochondrial | 1 | 1.12 | 0.400 | 0.86 | 0.365 | 0.93 | 0.427 | 0.74 | 0.351 | 1.14 | 0.390 | 1.00 | 0.494 | 0.97 | 0.483 |
| P19623 | Spermidine synthase | 1 | **0.31** | **0.034** | 0.60 | 0.171 | **0.29** | **0.020** | 0.62 | 0.189 | **0.11** | **0.012** | 0.59 | 0.172 | 0.59 | 0.174 |
| P19823 | Inter-alpha-trypsin inhibitor heavy chain H2 | 1 | 1.61 | 0.055 | 1.30 | 0.168 | **1.68** | **0.007** | 1.27 | 0.181 | **1.58** | **0.023** | 1.54 | 0.119 | 1.44 | 0.088 |
| P20810 | Calpastatin | 1 | 0.89 | 0.260 | 0.92 | 0.425 | 0.80 | 0.222 | 1.10 | 0.433 | 1.03 | 0.481 | 1.12 | 0.431 | 0.99 | 0.480 |
| P21399 | Cytoplasmic aconitate hydratase | 1 | 1.00 | 0.498 | 0.99 | 0.484 | 1.44 | 0.140 | 1.08 | 0.346 | 1.41 | 0.105 | 0.78 | 0.122 | 1.32 | 0.089 |
| P22059 | Oxysterol-binding protein 1 | 1 | 1.16 | 0.408 | 1.06 | 0.464 | 0.96 | 0.472 | 1.07 | 0.460 | 1.06 | 0.465 | 0.90 | 0.433 | 0.90 | 0.435 |
| P22307 | Non-specific lipid-transfer protein | 1 | 1.05 | 0.432 | 1.37 | 0.115 | 1.20 | 0.155 | **1.44** | **0.019** | **1.31** | **0.033** | 1.27 | 0.116 | 1.30 | 0.180 |
| P23142 | Fibulin-1 | 1 | 1.35 | 0.261 | 1.05 | 0.472 | 1.21 | 0.319 | 1.16 | 0.362 | 1.13 | 0.386 | 1.25 | 0.377 | 0.91 | 0.414 |
| P23381 | Tryptophan--tRNA ligase. cytoplasmic | 1 | **2.59** | **0.000** | 1.86 | 0.165 | **2.77** | **0.000** | **1.67** | **0.049** | **2.86** | **0.000** | **2.54** | **0.023** | 1.97 | 0.130 |
| P23919 | Thymidylate kinase | 1 | **3.02** | **0.001** | 1.78 | 0.104 | **6.58** | **0.000** | 1.31 | 0.175 | 0.90 | 0.395 | **6.67** | **0.000** | 1.76 | 0.248 |
| P23921 | Ribonucleoside-diphosphate reductase large subunit | 1 | 0.99 | 0.495 | 0.79 | 0.370 | 0.90 | 0.436 | 1.34 | 0.334 | 0.54 | 0.230 | 1.35 | 0.333 | 1.19 | 0.403 |
| P24534 | Elongation factor 1-beta | 1 | 0.71 | 0.136 | **0.69** | **0.007** | **0.60** | **0.001** | 0.76 | 0.241 | 0.67 | 0.056 | 0.72 | 0.093 | **0.70** | **0.012** |
| P25325 | 3-mercaptopyruvate sulfurtransferase | 1 | 1.13 | 0.394 | 0.90 | 0.358 | 1.02 | 0.474 | 1.13 | 0.354 | 1.14 | 0.356 | 1.10 | 0.403 | 1.03 | 0.466 |
| P25398 | 40S ribosomal protein S12 | 1 | 2.69 | 0.064 | 1.06 | 0.470 | **3.89** | **0.016** | 0.83 | 0.394 | 1.26 | 0.371 | **3.51** | **0.025** | 0.35 | 0.146 |
| P25788 | Proteasome subunit alpha type-3 | 1 | 1.62 | 0.175 | 1.76 | 0.129 | 1.39 | 0.258 | 2.12 | 0.065 | 1.52 | 0.210 | 1.87 | 0.108 | 1.85 | 0.115 |
| P26196 | Probable ATP-dependent RNA helicase DDX6 | 1 | 0.51 | 0.145 | 0.50 | 0.072 | 0.62 | 0.145 | 0.62 | 0.170 | 0.58 | 0.134 | 0.86 | 0.386 | 0.92 | 0.424 |
| P26358 | DNA (cytosine-5)-methyltransferase 1 | 1 | 0.82 | 0.238 | 1.01 | 0.485 | **0.73** | **0.033** | 1.18 | 0.181 | 0.86 | 0.378 | 1.27 | 0.132 | 0.82 | 0.253 |
| P26368 | Splicing factor U2AF 65 kDa subunit | 1 | 0.89 | 0.170 | 0.98 | 0.430 | 0.89 | 0.367 | 1.23 | 0.171 | 0.98 | 0.455 | 0.94 | 0.417 | 0.88 | 0.245 |
| P26639 | Threonine--tRNA ligase. cytoplasmic | 1 | 0.89 | 0.435 | 1.24 | 0.378 | 0.69 | 0.302 | 1.00 | 0.496 | 0.77 | 0.365 | 0.90 | 0.441 | 0.55 | 0.238 |
| P26640 | Valine--tRNA ligase | 1 | 0.66 | 0.203 | 0.70 | 0.232 | 0.95 | 0.451 | 0.87 | 0.386 | 0.56 | 0.133 | 0.64 | 0.190 | 0.90 | 0.414 |
| P27449 | V-type proton ATPase 16 kDa proteolipid subunit | 1 | 1.23 | 0.292 | 1.15 | 0.353 | 1.32 | 0.226 | 1.05 | 0.454 | 1.43 | 0.173 | 0.96 | 0.451 | 1.08 | 0.427 |
| P28070 | Proteasome subunit beta type-4 | 1 | 0.87 | 0.456 | 1.32 | 0.370 | 0.79 | 0.333 | 0.75 | 0.405 | 0.62 | 0.384 | 0.91 | 0.442 | 1.17 | 0.402 |
| P28074 | Proteasome subunit beta type-5 | 1 | 0.68 | 0.082 | 0.97 | 0.408 | **0.71** | **0.021** | 0.95 | 0.433 | **0.69** | **0.019** | **0.66** | **0.009** | 0.82 | 0.278 |
| P28838 | Cytosol aminopeptidase | 1 | **1.79** | **0.001** | 1.53 | 0.076 | **2.19** | **0.000** | 1.15 | 0.243 | **1.94** | **0.000** | **1.82** | **0.001** | 1.37 | 0.071 |
| P28907 | ADP-ribosyl cyclase/cyclic ADP-ribose hydrolase 1 | 1 | 1.37 | 0.177 | **1.38** | **0.016** | **1.82** | **0.017** | 1.29 | 0.115 | **1.77** | **0.026** | **1.76** | **0.015** | **1.80** | **0.009** |
| P29692 | Elongation factor 1-delta | 1 | 0.73 | 0.053 | **0.70** | **0.003** | **0.77** | **0.020** | **0.77** | **0.019** | **0.66** | **0.001** | **0.50** | **0.000** | **0.70** | **0.013** |
| P30043 | Flavin reductase (NADPH) | 1 | 1.64 | 0.119 | 1.22 | 0.356 | 1.23 | 0.282 | 1.16 | 0.338 | 1.60 | 0.160 | 1.26 | 0.286 | 1.43 | 0.228 |
| P30084 | Enoyl-CoA hydratase. mitochondrial | 1 | 1.12 | 0.376 | 0.73 | 0.245 | 0.90 | 0.357 | 0.68 | 0.228 | 1.11 | 0.388 | 0.75 | 0.276 | 0.93 | 0.432 |
| P30085 | UMP-CMP kinase | 1 | 0.52 | 0.156 | 0.81 | 0.353 | 0.77 | 0.328 | 1.17 | 0.391 | 0.75 | 0.317 | 0.57 | 0.186 | 1.01 | 0.494 |
| P30566 | Adenylosuccinate lyase | 1 | 0.50 | 0.123 | 0.60 | 0.177 | 0.57 | 0.157 | 1.25 | 0.338 | 0.46 | 0.102 | 0.60 | 0.184 | 0.82 | 0.358 |
| P30837 | Aldehyde dehydrogenase X. mitochondrial | 1 | 0.81 | 0.275 | 0.74 | 0.196 | 0.69 | 0.150 | 0.82 | 0.285 | 0.60 | 0.089 | 0.78 | 0.247 | 0.74 | 0.207 |
| P30876 | DNA-directed RNA polymerase II subunit RPB2 | 1 | 0.89 | 0.357 | 0.97 | 0.441 | 1.35 | 0.146 | 1.21 | 0.169 | 0.97 | 0.430 | 1.26 | 0.127 | 0.68 | 0.129 |
| P31040 | Succinate dehydrogenase [ubiquinone] flavoprotein subunit. mitochondrial | 1 | 1.47 | 0.222 | 1.12 | 0.318 | 1.49 | 0.194 | 0.98 | 0.483 | **1.86** | **0.003** | 1.51 | 0.083 | 1.43 | 0.089 |
| P31323 | cAMP-dependent protein kinase type II-beta regulatory subunit | 1 | **1.47** | **0.010** | 1.39 | 0.092 | 1.34 | 0.199 | 1.10 | 0.294 | 1.69 | 0.099 | 1.11 | 0.384 | **1.93** | **0.002** |
| P31949 | Protein S100-A11 | 1 | **1.65** | **0.010** | **1.66** | **0.006** | **1.71** | **0.006** | 0.93 | 0.383 | 1.47 | 0.151 | **1.59** | **0.028** | 0.99 | 0.488 |
| P32322 | Pyrroline-5-carboxylate reductase 1. mitochondrial | 1 | **3.26** | **0.016** | 1.31 | 0.320 | **3.20** | **0.017** | 1.77 | 0.158 | 1.87 | 0.138 | **2.53** | **0.049** | 1.19 | 0.391 |
| P33176 | Kinesin-1 heavy chain | 1 | 0.72 | 0.260 | 0.82 | 0.346 | 0.64 | 0.191 | 0.86 | 0.379 | 0.84 | 0.360 | 1.14 | 0.398 | 0.70 | 0.246 |
| P33316 | Deoxyuridine 5'-triphosphate nucleotidohydrolase. mitochondrial | 1 | 1.12 | 0.320 | 1.38 | 0.160 | 1.07 | 0.440 | 1.52 | 0.109 | **1.81** | **0.009** | 1.00 | 0.492 | 1.06 | 0.406 |
| P33992 | DNA replication licensing factor MCM5 | 1 | 1.16 | 0.382 | 1.31 | 0.287 | 0.99 | 0.486 | 1.33 | 0.275 | 0.85 | 0.370 | 1.15 | 0.389 | 1.16 | 0.386 |
| P35080 | Profilin-2 | 1 | **2.34** | **0.000** | 1.76 | 0.148 | 1.54 | 0.238 | 1.46 | 0.202 | 1.64 | 0.112 | **1.35** | **0.045** | 1.51 | 0.215 |
| P35237 | Serpin B6 | 1 | 1.50 | 0.160 | **2.25** | **0.019** | 1.28 | 0.273 | 1.62 | 0.115 | **2.17** | **0.036** | 1.71 | 0.092 | 1.75 | 0.085 |
| P35244 | Replication protein A 14 kDa subunit | 1 | 3.08 | 0.057 | 1.11 | 0.448 | 1.64 | 0.250 | 1.05 | 0.478 | 1.54 | 0.287 | 1.65 | 0.252 | 1.64 | 0.259 |
| P35606 | Coatomer subunit beta' | 1 | **0.42** | **0.037** | 0.66 | 0.176 | 0.71 | 0.224 | 0.53 | 0.087 | 0.59 | 0.127 | **0.34** | **0.014** | 0.81 | 0.317 |
| P36404 | ADP-ribosylation factor-like protein 2 | 1 | 2.03 | 0.089 | 1.82 | 0.161 | **2.17** | **0.028** | 1.28 | 0.380 | 2.20 | 0.106 | 1.93 | 0.074 | **2.35** | **0.000** |
| P36776 | Lon protease homolog. mitochondrial | 1 | 1.48 | 0.105 | 1.17 | 0.365 | 1.21 | 0.337 | 1.31 | 0.237 | 1.25 | 0.311 | 1.71 | 0.105 | 1.26 | 0.238 |
| P36871 | Phosphoglucomutase-1 | 1 | 0.66 | 0.164 | 1.08 | 0.427 | 0.74 | 0.237 | 1.05 | 0.459 | 0.66 | 0.164 | 0.78 | 0.280 | 0.90 | 0.399 |
| P36955 | Pigment epithelium-derived factor | 1 | 1.54 | 0.185 | 1.22 | 0.177 | 1.37 | 0.084 | 0.86 | 0.179 | 1.20 | 0.151 | 1.38 | 0.147 | 1.22 | 0.345 |
| P36957 | Dihydrolipoyllysine-residue succinyltransferase component of 2-oxoglutarate dehydrogenase complex. mitochondrial | 1 | **1.77** | **0.000** | 1.11 | 0.347 | **1.24** | **0.038** | 1.12 | 0.343 | **1.55** | **0.015** | 1.17 | 0.259 | 1.35 | 0.095 |
| P37268 | Squalene synthase | 1 | **3.10** | **0.008** | 1.61 | 0.167 | 1.76 | 0.186 | 0.61 | 0.191 | **2.94** | **0.026** | 1.59 | 0.175 | 1.54 | 0.198 |
| P38117 | Electron transfer flavoprotein subunit beta | 1 | 1.08 | 0.311 | 1.23 | 0.077 | **1.27** | **0.047** | 1.13 | 0.210 | 1.09 | 0.405 | 0.97 | 0.462 | 1.21 | 0.267 |
| P38606 | V-type proton ATPase catalytic subunit A | 1 | 1.21 | 0.436 | 1.53 | 0.184 | 1.96 | 0.071 | 1.01 | 0.493 | 1.95 | 0.176 | 1.14 | 0.398 | 2.09 | 0.120 |
| P39687 | Acidic leucine-rich nuclear phosphoprotein 32 family member A | 1 | 1.26 | 0.337 | 1.48 | 0.229 | 0.89 | 0.416 | 1.70 | 0.157 | 1.25 | 0.343 | 1.22 | 0.360 | 1.13 | 0.418 |
| P39748 | Flap endonuclease 1 | 1 | 1.28 | 0.335 | 2.04 | 0.096 | 1.51 | 0.230 | 1.95 | 0.113 | 1.72 | 0.169 | 1.69 | 0.176 | 1.93 | 0.121 |
| P40227 | T-complex protein 1 subunit zeta | 1 | 1.50 | 0.069 | 1.58 | 0.152 | 1.03 | 0.481 | 0.81 | 0.329 | 1.35 | 0.364 | 1.64 | 0.130 | 1.32 | 0.267 |
| P40261 | Nicotinamide N-methyltransferase | 1 | 0.89 | 0.427 | 1.58 | 0.228 | 1.41 | 0.286 | 1.21 | 0.384 | 0.87 | 0.418 | 1.87 | 0.152 | 0.69 | 0.301 |
| P40616 | ADP-ribosylation factor-like protein 1 | 1 | 1.72 | 0.074 | 1.68 | 0.059 | **1.49** | **0.014** | 1.09 | 0.337 | 1.47 | 0.182 | 1.27 | 0.297 | 1.62 | 0.110 |
| P40925 | Malate dehydrogenase. cytoplasmic | 1 | 0.94 | 0.410 | 0.88 | 0.356 | 0.84 | 0.335 | **0.63** | **0.006** | **0.68** | **0.012** | 0.92 | 0.401 | 1.20 | 0.170 |
| P41227 | N-alpha-acetyltransferase 10 | 1 | 0.52 | 0.137 | 0.76 | 0.310 | 0.88 | 0.407 | 0.66 | 0.226 | 0.91 | 0.427 | 0.63 | 0.210 | 0.82 | 0.364 |
| P42285 | Superkiller viralicidic activity 2-like 2 | 1 | 0.93 | 0.435 | 0.88 | 0.382 | 0.76 | 0.265 | 0.80 | 0.311 | 0.90 | 0.404 | 0.72 | 0.236 | 0.80 | 0.311 |
| P42766 | 60S ribosomal protein L35 | 1 | 0.83 | 0.121 | **0.68** | **0.001** | 0.76 | 0.191 | 0.69 | 0.065 | 0.80 | 0.148 | 0.60 | 0.152 | 0.79 | 0.251 |
| P46060 | Ran GTPase-activating protein 1 | 1 | **3.47** | **0.000** | 0.97 | 0.459 | **4.67** | **0.000** | 1.22 | 0.295 | 0.62 | 0.132 | **3.50** | **0.000** | 0.95 | 0.417 |
| P46459 | Vesicle-fusing ATPase | 1 | 1.22 | 0.381 | 1.47 | 0.262 | 1.38 | 0.297 | 0.73 | 0.317 | 0.70 | 0.305 | 1.25 | 0.364 | 0.97 | 0.477 |
| P46778 | 60S ribosomal protein L21 | 1 | 0.99 | 0.486 | 1.14 | 0.373 | 1.04 | 0.461 | 0.97 | 0.471 | 1.35 | 0.180 | 1.00 | 0.493 | 1.09 | 0.429 |
| P47914 | 60S ribosomal protein L29 | 1 | **0.61** | **0.001** | **0.71** | **0.001** | **0.57** | **0.000** | **0.69** | **0.002** | **0.64** | **0.001** | **0.64** | **0.000** | 0.67 | 0.123 |
| P48047 | ATP synthase subunit O. mitochondrial | 1 | 1.09 | 0.403 | 1.02 | 0.476 | 0.91 | 0.383 | 0.77 | 0.225 | 1.15 | 0.343 | 0.97 | 0.466 | 0.94 | 0.428 |
| P48163 | NADP-dependent malic enzyme | 1 | 0.74 | 0.290 | 0.78 | 0.407 | 0.79 | 0.320 | 1.20 | 0.358 | 1.03 | 0.489 | 1.34 | 0.279 | 1.32 | 0.337 |
| P48506 | Glutamate--cysteine ligase catalytic subunit | 1 | **2.01** | **0.032** | 1.24 | 0.380 | 1.27 | 0.342 | 1.25 | 0.406 | 1.06 | 0.461 | **1.60** | **0.027** | 1.08 | 0.462 |
| P48556 | 26S proteasome non-ATPase regulatory subunit 8 | 1 | 0.82 | 0.298 | 0.90 | 0.228 | **0.70** | **0.022** | 0.90 | 0.238 | 0.92 | 0.380 | **0.63** | **0.043** | 0.93 | 0.369 |
| P49406 | 39S ribosomal protein L19. mitochondrial | 1 | 1.45 | 0.155 | 1.39 | 0.237 | 1.32 | 0.279 | 1.26 | 0.289 | 1.03 | 0.471 | 1.39 | 0.281 | 1.20 | 0.202 |
| P49427 | Ubiquitin-conjugating enzyme E2 R1 | 1 | 0.95 | 0.456 | 0.78 | 0.249 | 1.08 | 0.421 | 1.28 | 0.249 | 1.06 | 0.442 | 0.64 | 0.124 | 1.28 | 0.257 |
| P49588 | Alanine--tRNA ligase. cytoplasmic | 1 | 0.86 | 0.403 | 0.30 | 0.056 | 0.93 | 0.447 | 0.66 | 0.255 | 0.24 | 0.059 | 1.27 | 0.346 | 0.43 | 0.131 |
| P49591 | Serine--tRNA ligase. cytoplasmic | 1 | 1.38 | 0.221 | 1.43 | 0.190 | 1.08 | 0.432 | 1.44 | 0.185 | 1.11 | 0.403 | 1.33 | 0.288 | 1.44 | 0.194 |
| P49720 | Proteasome subunit beta type-3 | 1 | 1.27 | 0.149 | **1.84** | **0.002** | 1.16 | 0.272 | 1.17 | 0.295 | 1.28 | 0.249 | 1.37 | 0.147 | 1.06 | 0.426 |
| P49753 | Acyl-coenzyme A thioesterase 2. mitochondrial | 1 | 0.67 | 0.239 | 0.78 | 0.291 | 0.68 | 0.195 | 0.86 | 0.369 | 0.74 | 0.354 | 1.26 | 0.302 | 0.35 | 0.073 |
| P49821 | NADH dehydrogenase [ubiquinone] flavoprotein 1. mitochondrial | 1 | 0.85 | 0.358 | 0.90 | 0.404 | 0.98 | 0.476 | 0.82 | 0.328 | 0.94 | 0.441 | 1.08 | 0.430 | 0.92 | 0.422 |
| P49915 | GMP synthase [glutamine-hydrolyzing] | 1 | 0.79 | 0.281 | 0.75 | 0.237 | 0.67 | 0.342 | 1.06 | 0.446 | 0.62 | 0.129 | 0.82 | 0.306 | 0.87 | 0.369 |
| P49916 | DNA ligase 3 | 1 | 1.67 | 0.183 | 1.45 | 0.256 | 1.08 | 0.451 | 0.66 | 0.248 | 0.87 | 0.410 | 1.22 | 0.368 | 1.25 | 0.356 |
| P50416 | Carnitine O-palmitoyltransferase 1. liver isoform | 1 | 1.09 | 0.459 | 0.80 | 0.339 | 1.27 | 0.322 | 0.65 | 0.230 | 0.86 | 0.394 | 0.67 | 0.249 | 0.80 | 0.383 |
| P51149 | Ras-related protein Rab-7a | 1 | 1.01 | 0.487 | 1.12 | 0.273 | 1.05 | 0.376 | 1.03 | 0.387 | 1.24 | 0.089 | 0.95 | 0.347 | 1.11 | 0.316 |
| P51572 | B-cell receptor-associated protein 31 | 1 | 0.78 | 0.283 | 0.82 | 0.314 | 0.70 | 0.195 | 0.97 | 0.469 | 0.92 | 0.420 | 0.79 | 0.289 | 1.07 | 0.448 |
| P52907 | F-actin-capping protein subunit alpha-1 | 1 | 0.97 | 0.476 | 0.94 | 0.423 | 0.85 | 0.318 | 1.32 | 0.156 | 1.21 | 0.227 | 1.29 | 0.237 | 0.57 | 0.068 |
| P53004 | Biliverdin reductase A | 1 | 0.90 | 0.413 | 1.18 | 0.360 | 1.00 | 0.496 | 1.02 | 0.489 | 1.32 | 0.275 | 1.06 | 0.453 | 1.37 | 0.252 |
| P53621 | Coatomer subunit alpha | 1 | 1.38 | 0.180 | 0.97 | 0.455 | 1.24 | 0.297 | 1.05 | 0.434 | 1.39 | 0.193 | 1.31 | 0.293 | 1.09 | 0.358 |
| P53999 | Activated RNA polymerase II transcriptional coactivator p15 | 1 | **0.53** | **0.004** | **0.62** | **0.006** | 0.50 | 0.065 | **0.54** | **0.025** | **0.51** | **0.000** | 0.63 | 0.062 | **0.60** | **0.005** |
| P54577 | Tyrosine--tRNA ligase. cytoplasmic | 1 | 0.84 | 0.344 | 0.70 | 0.205 | 0.52 | 0.073 | 0.66 | 0.171 | 0.49 | 0.067 | **0.41** | **0.034** | 0.67 | 0.184 |
| P54578 | Ubiquitin carboxyl-terminal hydrolase 14 | 1 | 1.07 | 0.403 | 1.06 | 0.403 | 1.24 | 0.252 | 1.09 | 0.296 | 1.03 | 0.452 | 1.02 | 0.447 | 1.23 | 0.199 |
| P55010 | Eukaryotic translation initiation factor 5 | 1 | 1.02 | 0.471 | 0.79 | 0.283 | 0.78 | 0.066 | 0.99 | 0.466 | 0.85 | 0.167 | **0.67** | **0.011** | 0.77 | 0.200 |
| P55735 | Protein SEC13 homolog | 1 | **0.38** | **0.000** | 0.82 | 0.161 | **0.41** | **0.001** | **0.60** | **0.023** | 0.69 | 0.179 | **0.50** | **0.005** | **0.57** | **0.019** |
| P60866 | 40S ribosomal protein S20 | 1 | 0.77 | 0.050 | 0.86 | 0.280 | 0.82 | 0.178 | 0.92 | 0.261 | **0.57** | **0.000** | 0.66 | 0.071 | 0.81 | 0.146 |
| P60900 | Proteasome subunit alpha type-6 | 1 | 0.86 | 0.364 | 1.40 | 0.213 | 0.74 | 0.244 | 1.12 | 0.396 | 0.59 | 0.122 | 0.74 | 0.249 | 0.57 | 0.110 |
| P61019 | Ras-related protein Rab-2A | 1 | 1.02 | 0.488 | 1.40 | 0.272 | 1.05 | 0.466 | 1.00 | 0.495 | 1.69 | 0.171 | 0.54 | 0.166 | 1.62 | 0.193 |
| P61163 | Alpha-centractin | 1 | 1.69 | 0.280 | 2.30 | 0.166 | 3.03 | 0.095 | 2.11 | 0.195 | 2.83 | 0.114 | 3.13 | 0.091 | 2.38 | 0.163 |
| P61353 | 60S ribosomal protein L27 | 1 | 0.51 | 0.084 | 0.59 | 0.069 | **0.38** | **0.005** | 0.67 | 0.127 | **0.47** | **0.023** | **0.52** | **0.036** | 0.67 | 0.181 |
| P61457 | Pterin-4-alpha-carbinolamine dehydratase | 1 | 0.68 | 0.227 | 0.73 | 0.350 | 0.96 | 0.458 | 0.88 | 0.392 | 0.63 | 0.186 | 1.08 | 0.468 | 0.95 | 0.452 |
| P62136 | Serine/threonine-protein phosphatase PP1-alpha catalytic subunit | 1 | **1.20** | **0.033** | 1.13 | 0.213 | 1.03 | 0.441 | **1.34** | **0.021** | **1.56** | **0.000** | 1.00 | 0.499 | **1.24** | **0.002** |
| P62304 | Small nuclear ribonucleoprotein E | 1 | 1.13 | 0.364 | 0.99 | 0.474 | 1.01 | 0.490 | 1.03 | 0.472 | 1.25 | 0.241 | 0.96 | 0.438 | 0.98 | 0.462 |
| P62308 | Small nuclear ribonucleoprotein G | 1 | 0.50 | 0.143 | 0.91 | 0.430 | 0.37 | 0.065 | 0.54 | 0.159 | 0.48 | 0.138 | 0.70 | 0.276 | 0.73 | 0.307 |
| P62857 | 40S ribosomal protein S28 | 1 | 0.92 | 0.439 | 1.17 | 0.419 | 0.79 | 0.298 | 0.83 | 0.336 | 0.63 | 0.165 | 1.12 | 0.407 | 0.72 | 0.248 |
| P62861 | 40S ribosomal protein S30 | 1 | 0.78 | 0.168 | 0.88 | 0.146 | 0.88 | 0.275 | 1.03 | 0.441 | 0.96 | 0.359 | **0.71** | **0.001** | 0.90 | 0.316 |
| P62879 | Guanine nucleotide-binding protein G(I)/G(S)/G(T) subunit beta-2 | 1 | 1.19 | 0.381 | 1.41 | 0.267 | 1.04 | 0.471 | 1.47 | 0.240 | 0.88 | 0.412 | 1.20 | 0.375 | 0.94 | 0.457 |
| P63000 | Ras-related C3 botulinum toxin substrate 1 | 1 | 0.94 | 0.454 | 0.97 | 0.472 | 1.16 | 0.389 | 1.53 | 0.207 | 1.44 | 0.247 | 0.77 | 0.322 | 1.11 | 0.429 |
| P63173 | 60S ribosomal protein L38 | 1 | **0.65** | **0.016** | **0.65** | **0.013** | 0.81 | 0.174 | 0.94 | 0.298 | **0.69** | **0.033** | **0.37** | **0.000** | 0.98 | 0.424 |
| P63208 | S-phase kinase-associated protein 1 | 1 | **0.59** | **0.012** | 0.77 | 0.059 | 0.72 | 0.189 | 0.79 | 0.267 | 1.01 | 0.490 | 0.79 | 0.263 | 0.82 | 0.325 |
| P67775 | Serine/threonine-protein phosphatase 2A catalytic subunit alpha isoform | 1 | **0.31** | **0.026** | 0.82 | 0.346 | 0.52 | 0.102 | 0.61 | 0.172 | 0.63 | 0.187 | 0.92 | 0.433 | 0.92 | 0.432 |
| P67870 | Casein kinase II subunit beta | 1 | 1.60 | 0.288 | 1.98 | 0.064 | 1.15 | 0.388 | 2.02 | 0.059 | 1.66 | 0.224 | 1.83 | 0.093 | 1.20 | 0.390 |
| P68402 | Platelet-activating factor acetylhydrolase IB subunit beta | 1 | 1.23 | 0.310 | 1.22 | 0.314 | 1.31 | 0.255 | 0.94 | 0.437 | 1.25 | 0.295 | 1.27 | 0.285 | 1.23 | 0.313 |
| P78386 | Keratin. type II cuticular Hb5 | 1 | **0.37** | **0.025** | 0.72 | 0.239 | **0.26** | **0.004** | **0.42** | **0.036** | **0.26** | **0.007** | **0.38** | **0.027** | **0.32** | **0.017** |
| P82650 | 28S ribosomal protein S22. mitochondrial | 1 | 1.39 | 0.265 | 1.00 | 0.500 | 1.31 | 0.257 | 1.23 | 0.310 | 1.00 | 0.493 | 1.54 | 0.149 | 1.05 | 0.461 |
| P84085 | ADP-ribosylation factor 5 | 1 | 0.68 | 0.235 | 0.87 | 0.391 | 0.91 | 0.422 | 0.95 | 0.454 | 0.61 | 0.188 | 0.83 | 0.361 | 0.60 | 0.183 |
| P84103 | Serine/arginine-rich splicing factor 3 | 1 | **0.79** | **0.005** | 0.87 | 0.163 | 0.85 | 0.218 | 0.99 | 0.471 | **0.78** | **0.016** | 0.83 | 0.237 | 0.90 | 0.249 |
| P99999 | Cytochrome c | 1 | 1.49 | 0.149 | 0.93 | 0.423 | 1.28 | 0.258 | 1.05 | 0.453 | 1.68 | 0.088 | 1.16 | 0.355 | 1.28 | 0.264 |
| Q01469 | Fatty acid-binding protein. epidermal | 1 | 0.48 | 0.133 | 1.16 | 0.399 | 0.41 | 0.081 | 1.15 | 0.406 | 0.78 | 0.338 | 0.78 | 0.338 | 0.57 | 0.193 |
| Q02790 | Peptidyl-prolyl cis-trans isomerase FKBP4 | 1 | 1.52 | 0.285 | 1.86 | 0.189 | 1.03 | 0.488 | 1.47 | 0.296 | 1.79 | 0.210 | 1.62 | 0.252 | 1.49 | 0.298 |
| Q02978 | Mitochondrial 2-oxoglutarate/malate carrier protein | 1 | 1.61 | 0.075 | **1.47** | **0.010** | 1.26 | 0.187 | 1.20 | 0.252 | **1.77** | **0.006** | 1.41 | 0.151 | 1.44 | 0.061 |
| Q04837 | Single-stranded DNA-binding protein. mitochondrial | 1 | 1.32 | 0.238 | 1.44 | 0.105 | **1.57** | **0.040** | 1.21 | 0.199 | **1.67** | **0.001** | **1.71** | **0.004** | 1.52 | 0.085 |
| Q05519 | Serine/arginine-rich splicing factor 11 | 1 | 0.97 | 0.472 | 0.77 | 0.273 | 0.95 | 0.447 | 0.69 | 0.200 | 1.02 | 0.490 | 0.79 | 0.297 | 0.76 | 0.271 |
| Q06210 | Glutamine--fructose-6-phosphate aminotransferase [isomerizing] 1 | 1 | 0.68 | 0.242 | 0.66 | 0.224 | 0.68 | 0.233 | 0.97 | 0.477 | 0.68 | 0.251 | 0.46 | 0.098 | 0.63 | 0.210 |
| Q06323 | Proteasome activator complex subunit 1 | 1 | 1.90 | 0.086 | **2.59** | **0.018** | **2.28** | **0.034** | **2.36** | **0.029** | 1.77 | 0.112 | **2.39** | **0.028** | **2.26** | **0.040** |
| Q07065 | Cytoskeleton-associated protein 4 | 1 | 0.99 | 0.481 | 1.28 | 0.134 | 1.12 | 0.336 | 0.89 | 0.362 | 1.09 | 0.347 | 1.21 | 0.138 | 1.16 | 0.281 |
| Q07812 | Apoptosis regulator BAX | 1 | **0.46** | **0.022** | **0.41** | **0.009** | **0.47** | **0.022** | **0.36** | **0.004** | **0.50** | **0.037** | 0.70 | 0.160 | **0.26** | **0.001** |
| Q08380 | Galectin-3-binding protein | 1 | 0.99 | 0.490 | 0.55 | 0.175 | 0.93 | 0.444 | 1.01 | 0.495 | 0.39 | 0.095 | 0.61 | 0.275 | 0.94 | 0.452 |
| Q08945 | FACT complex subunit SSRP1 | 1 | 0.91 | 0.425 | 0.80 | 0.329 | 0.80 | 0.326 | 0.88 | 0.395 | 1.17 | 0.377 | 0.51 | 0.105 | 0.66 | 0.215 |
| Q10471 | Polypeptide N-acetylgalactosaminyltransferase 2 | 1 | 0.66 | 0.186 | 0.71 | 0.226 | 0.52 | 0.078 | 0.66 | 0.181 | 0.60 | 0.139 | 0.54 | 0.095 | 0.60 | 0.141 |
| Q10567 | AP-1 complex subunit beta-1 | 1 | 0.58 | 0.144 | 0.64 | 0.185 | 0.73 | 0.259 | 0.58 | 0.139 | 0.71 | 0.251 | 0.52 | 0.106 | **0.39** | **0.048** |
| Q12874 | Splicing factor 3A subunit 3 | 1 | 0.99 | 0.471 | 1.08 | 0.316 | 0.79 | 0.061 | 1.13 | 0.287 | **0.67** | **0.004** | 0.82 | 0.209 | 0.73 | 0.098 |
| Q12907 | Vesicular integral-membrane protein VIP36 | 1 | 1.55 | 0.163 | 1.69 | 0.113 | 1.79 | 0.087 | 1.07 | 0.441 | 1.74 | 0.105 | 1.59 | 0.147 | 1.50 | 0.185 |
| Q13011 | Delta(3.5)-Delta(2.4)-dienoyl-CoA isomerase. mitochondrial | 1 | 0.80 | 0.369 | 0.81 | 0.228 | 0.73 | 0.228 | 0.83 | 0.260 | 0.82 | 0.315 | **0.63** | **0.000** | **0.55** | **0.046** |
| Q13043 | Serine/threonine-protein kinase 4 | 1 | 1.36 | 0.324 | 1.13 | 0.429 | 1.54 | 0.253 | 1.66 | 0.218 | 1.15 | 0.423 | 0.53 | 0.209 | 1.65 | 0.226 |
| Q13045 | Protein flightless-1 homolog | 1 | 0.97 | 0.465 | 0.88 | 0.277 | 0.76 | 0.183 | 1.04 | 0.438 | 0.81 | 0.253 | 0.83 | 0.297 | 0.53 | 0.105 |
| Q13098 | COP9 signalosome complex subunit 1 | 1 | 1.21 | 0.329 | **1.64** | **0.015** | 1.37 | 0.176 | 0.95 | 0.412 | 1.14 | 0.309 | 1.52 | 0.102 | 0.91 | 0.358 |
| Q13162 | Peroxiredoxin-4 | 1 | 0.95 | 0.467 | 1.72 | 0.200 | 2.30 | 0.092 | 2.72 | 0.055 | 2.76 | 0.054 | **2.95** | **0.042** | 1.41 | 0.308 |
| Q13200 | 26S proteasome non-ATPase regulatory subunit 2 | 1 | 1.41 | 0.273 | 1.28 | 0.331 | 1.99 | 0.099 | 1.01 | 0.497 | 1.67 | 0.180 | 1.44 | 0.261 | 1.31 | 0.356 |
| Q13242 | Serine/arginine-rich splicing factor 9 | 1 | 1.01 | 0.498 | 1.17 | 0.377 | 1.02 | 0.483 | 1.28 | 0.234 | 1.12 | 0.373 | 1.03 | 0.474 | 1.11 | 0.390 |
| Q13263 | Transcription intermediary factor 1-beta | 1 | 0.72 | 0.275 | 0.82 | 0.355 | 1.34 | 0.286 | 0.79 | 0.333 | 0.39 | 0.073 | 0.62 | 0.201 | 1.19 | 0.374 |
| Q13404 | Ubiquitin-conjugating enzyme E2 variant 1 | 1 | 0.90 | 0.244 | 1.03 | 0.463 | 0.97 | 0.438 | 0.94 | 0.330 | 1.02 | 0.465 | 0.94 | 0.364 | 0.91 | 0.268 |
| Q13418 | Integrin-linked protein kinase | 1 | 1.26 | 0.228 | 1.25 | 0.097 | 1.51 | 0.063 | 1.08 | 0.372 | 1.50 | 0.080 | **1.65** | **0.002** | 1.37 | 0.093 |
| Q13425 | Beta-2-syntrophin | 1 | 0.92 | 0.425 | 1.00 | 0.498 | 1.01 | 0.495 | 1.24 | 0.305 | 1.21 | 0.325 | 1.51 | 0.230 | 1.11 | 0.410 |
| Q13501 | Sequestosome-1 | 1 | 0.93 | 0.444 | 1.07 | 0.452 | 0.37 | 0.053 | 1.51 | 0.216 | 0.89 | 0.418 | 1.04 | 0.472 | 1.10 | 0.433 |
| Q13586 | Stromal interaction molecule 1 | 1 | 0.86 | 0.339 | **0.72** | **0.039** | 0.71 | 0.225 | 0.78 | 0.222 | 0.84 | 0.234 | 0.73 | 0.219 | 0.61 | 0.139 |
| Q13595 | Transformer-2 protein homolog alpha | 1 | 1.43 | 0.257 | 1.31 | 0.309 | 1.79 | 0.136 | 0.73 | 0.291 | 1.01 | 0.493 | 0.76 | 0.316 | 0.95 | 0.461 |
| Q13596 | Sorting nexin-1 | 1 | 2.27 | 0.078 | 1.89 | 0.134 | 1.79 | 0.256 | **2.54** | **0.050** | 1.78 | 0.183 | 2.33 | 0.070 | 1.68 | 0.194 |
| Q13617 | Cullin-2 | 1 | 0.93 | 0.290 | 1.05 | 0.425 | 1.02 | 0.471 | 1.14 | 0.196 | 1.19 | 0.297 | 1.09 | 0.362 | 1.12 | 0.375 |
| Q13620 | Cullin-4B | 1 | 1.88 | 0.212 | 0.82 | 0.405 | 1.16 | 0.429 | 0.89 | 0.444 | 1.43 | 0.333 | 1.10 | 0.454 | 0.63 | 0.319 |
| Q13867 | Bleomycin hydrolase | 1 | 1.40 | 0.284 | 1.29 | 0.333 | 1.66 | 0.185 | 0.95 | 0.464 | 1.40 | 0.285 | 1.56 | 0.222 | 1.43 | 0.275 |
| Q14103 | Heterogeneous nuclear ribonucleoprotein D0 | 1 | 0.82 | 0.367 | 1.13 | 0.415 | 0.72 | 0.287 | 1.01 | 0.497 | 0.89 | 0.424 | 0.83 | 0.379 | 1.30 | 0.326 |
| Q14116 | Interleukin-18 | 1 | 0.89 | 0.421 | 0.62 | 0.210 | 0.77 | 0.323 | 1.26 | 0.340 | 0.96 | 0.469 | 0.48 | 0.126 | 0.48 | 0.137 |
| Q14247 | Src substrate cortactin | 1 | 1.05 | 0.412 | 0.82 | 0.317 | 1.01 | 0.490 | 0.93 | 0.397 | 0.94 | 0.434 | 0.98 | 0.465 | 0.86 | 0.326 |
| Q14254 | Flotillin-2 | 1 | 0.74 | 0.212 | 1.37 | 0.214 | 0.69 | 0.305 | 1.59 | 0.116 | 1.45 | 0.203 | **1.64** | **0.017** | 1.02 | 0.489 |
| Q14258 | E3 ubiquitin/ISG15 ligase TRIM25 | 1 | 1.25 | 0.359 | 1.01 | 0.495 | 1.23 | 0.364 | 1.04 | 0.479 | 0.74 | 0.324 | 1.27 | 0.349 | 1.35 | 0.314 |
| Q14344 | Guanine nucleotide-binding protein subunit alpha-13 | 1 | 1.51 | 0.263 | 0.66 | 0.275 | 1.30 | 0.342 | 1.60 | 0.230 | 1.45 | 0.284 | 1.61 | 0.227 | 0.65 | 0.284 |
| Q14558 | Phosphoribosyl pyrophosphate synthase-associated protein 1 | 1 | 0.37 | 0.075 | 0.46 | 0.108 | 0.63 | 0.214 | 0.61 | 0.201 | **0.24** | **0.040** | 0.59 | 0.195 | 0.37 | 0.085 |
| Q14657 | EKC/KEOPS complex subunit LAGE3 | 1 | 0.94 | 0.461 | 1.30 | 0.369 | 0.94 | 0.439 | 0.86 | 0.426 | 0.99 | 0.492 | 0.84 | 0.373 | 1.13 | 0.452 |
| Q14692 | Ribosome biogenesis protein BMS1 homolog | 1 | 1.65 | 0.155 | 2.11 | 0.057 | 1.44 | 0.227 | 1.33 | 0.283 | 2.18 | 0.053 | 1.57 | 0.180 | 1.52 | 0.204 |
| Q14694 | Ubiquitin carboxyl-terminal hydrolase 10 | 1 | 0.60 | 0.157 | 0.87 | 0.399 | 0.69 | 0.214 | 1.04 | 0.468 | 1.09 | 0.427 | 0.75 | 0.291 | 0.62 | 0.181 |
| Q14764 | Major vault protein | 1 | 0.88 | 0.436 | 0.63 | 0.289 | 1.29 | 0.365 | 1.60 | 0.263 | 0.94 | 0.465 | 2.18 | 0.140 | 1.02 | 0.493 |
| Q14914 | Prostaglandin reductase 1 | 1 | 0.82 | 0.319 | 0.98 | 0.474 | 1.00 | 0.499 | 0.98 | 0.474 | 0.95 | 0.448 | 0.71 | 0.218 | 0.83 | 0.335 |
| Q15019 | Septin-2 | 1 | 0.84 | 0.212 | 0.64 | 0.057 | 0.59 | 0.171 | 0.89 | 0.378 | 0.65 | 0.106 | 0.89 | 0.431 | 1.05 | 0.464 |
| Q15021 | Condensin complex subunit 1 | 1 | 1.07 | 0.468 | 1.58 | 0.271 | 1.66 | 0.246 | 2.45 | 0.108 | 1.24 | 0.396 | 2.58 | 0.096 | 1.26 | 0.387 |
| Q15067 | Peroxisomal acyl-coenzyme A oxidase 1 | 1 | 1.00 | 0.497 | 1.02 | 0.491 | 0.57 | 0.166 | 0.80 | 0.348 | 0.68 | 0.263 | 0.86 | 0.391 | 0.64 | 0.234 |
| Q15126 | Phosphomevalonate kinase | 1 | **1.64** | **0.027** | **1.62** | **0.006** | **1.66** | **0.014** | 1.33 | 0.109 | **1.86** | **0.000** | 1.29 | 0.114 | **1.81** | **0.000** |
| Q15293 | Reticulocalbin-1 | 1 | 0.55 | 0.094 | 0.80 | 0.322 | 0.83 | 0.322 | 0.91 | 0.450 | 0.56 | 0.103 | 0.83 | 0.329 | 1.00 | 0.496 |
| Q15436 | Protein transport protein Sec23A | 1 | 1.29 | 0.289 | 1.28 | 0.196 | 1.10 | 0.418 | 1.39 | 0.203 | **1.40** | **0.025** | 1.13 | 0.287 | 1.36 | 0.295 |
| Q15459 | Splicing factor 3A subunit 1 | 1 | 0.97 | 0.433 | 1.09 | 0.427 | 0.80 | 0.114 | 1.00 | 0.498 | 1.14 | 0.290 | 0.83 | 0.343 | 0.83 | 0.271 |
| Q15645 | Pachytene checkpoint protein 2 homolog | 1 | 0.64 | 0.188 | 0.71 | 0.238 | 0.59 | 0.138 | 0.61 | 0.161 | 0.67 | 0.211 | 0.66 | 0.205 | 0.51 | 0.101 |
| Q15717 | ELAV-like protein 1 | 1 | 0.79 | 0.124 | 0.96 | 0.456 | 0.81 | 0.181 | 0.90 | 0.335 | 0.69 | 0.222 | **0.59** | **0.049** | 0.72 | 0.166 |
| Q15758 | Neutral amino acid transporter B(0) | 1 | 0.99 | 0.470 | 1.09 | 0.394 | 1.09 | 0.372 | 0.77 | 0.191 | 1.19 | 0.328 | 1.06 | 0.459 | 0.92 | 0.424 |
| Q15833 | Syntaxin-binding protein 2 | 1 | 1.74 | 0.182 | 1.96 | 0.128 | 2.04 | 0.113 | 0.87 | 0.415 | 1.32 | 0.332 | 1.64 | 0.210 | 1.12 | 0.433 |
| Q16539 | Mitogen-activated protein kinase 14 | 1 | 1.14 | 0.385 | 1.24 | 0.301 | 1.04 | 0.462 | 0.93 | 0.426 | 0.91 | 0.412 | 1.08 | 0.447 | 0.87 | 0.378 |
| Q16563 | Synaptophysin-like protein 1 | 1 | **0.58** | **0.000** | 1.01 | 0.490 | **0.70** | **0.009** | 1.07 | 0.440 | 0.94 | 0.347 | 0.92 | 0.413 | 0.92 | 0.403 |
| Q16576 | Histone-binding protein RBBP7 | 1 | **0.50** | **0.045** | 0.56 | 0.070 | 0.62 | 0.103 | 0.64 | 0.122 | 0.56 | 0.079 | 0.52 | 0.052 | 0.59 | 0.094 |
| Q16629 | Serine/arginine-rich splicing factor 7 | 1 | **0.71** | **0.000** | **0.72** | **0.001** | **0.71** | **0.004** | 0.86 | 0.068 | **0.68** | **0.001** | **0.67** | **0.000** | 0.71 | 0.051 |
| Q16836 | Hydroxyacyl-coenzyme A dehydrogenase. mitochondrial | 1 | 1.73 | 0.249 | 2.53 | 0.114 | 1.85 | 0.217 | 2.04 | 0.181 | 3.01 | 0.077 | 2.20 | 0.159 | 1.64 | 0.275 |
| Q16891 | MICOS complex subunit MIC60 | 1 | **0.72** | **0.015** | 0.85 | 0.138 | **0.72** | **0.037** | 0.90 | 0.249 | **0.74** | **0.037** | 0.81 | 0.071 | **0.75** | **0.037** |
| Q2NL82 | Pre-rRNA-processing protein TSR1 homolog | 1 | 0.49 | 0.082 | 0.49 | 0.078 | 0.59 | 0.134 | 0.86 | 0.373 | 0.80 | 0.319 | 0.59 | 0.147 | 0.73 | 0.257 |
| Q3ZCQ8 | Mitochondrial import inner membrane translocase subunit TIM50 | 1 | 0.97 | 0.466 | 0.83 | 0.294 | 0.98 | 0.468 | 0.92 | 0.399 | 0.81 | 0.336 | 0.75 | 0.205 | 0.87 | 0.350 |
| Q53GQ0 | Very-long-chain 3-oxoacyl-CoA reductase | 1 | 1.33 | 0.270 | 1.11 | 0.452 | 1.58 | 0.179 | 0.93 | 0.464 | 1.68 | 0.235 | 2.03 | 0.151 | 1.55 | 0.068 |
| Q53H82 | Beta-lactamase-like protein 2 | 1 | 0.40 | 0.055 | 0.52 | 0.109 | 0.62 | 0.175 | 1.00 | 0.495 | 0.58 | 0.158 | 0.75 | 0.290 | 0.50 | 0.106 |
| Q56VL3 | OCIA domain-containing protein 2 | 1 | 1.25 | 0.331 | 1.09 | 0.444 | 1.82 | 0.103 | 1.47 | 0.217 | 1.45 | 0.336 | 1.25 | 0.331 | 1.03 | 0.482 |
| Q5JPE7 | Nodal modulator 2 | 1 | 0.77 | 0.310 | 1.08 | 0.444 | 1.14 | 0.403 | 0.81 | 0.345 | 0.78 | 0.320 | 0.81 | 0.342 | 0.82 | 0.355 |
| Q5JTH9 | RRP12-like protein | 1 | 1.44 | 0.078 | 0.98 | 0.457 | 1.25 | 0.081 | 0.98 | 0.474 | 1.15 | 0.269 | **1.67** | **0.002** | 0.96 | 0.435 |
| Q5JTV8 | Torsin-1A-interacting protein 1 | 1 | 0.51 | 0.077 | 0.77 | 0.273 | 0.49 | 0.061 | 0.85 | 0.355 | 0.81 | 0.317 | 0.64 | 0.162 | 0.70 | 0.218 |
| Q5JWF2 | Guanine nucleotide-binding protein G(s) subunit alpha isoforms XLas | 1 | 0.87 | 0.409 | 0.96 | 0.459 | 0.99 | 0.483 | 1.05 | 0.467 | 1.14 | 0.376 | 1.03 | 0.483 | 0.86 | 0.365 |
| Q5T6V5 | UPF0553 protein C9orf64 | 1 | 1.92 | 0.132 | **2.68** | **0.001** | 1.97 | 0.132 | **2.18** | **0.018** | 2.19 | 0.067 | 2.22 | 0.060 | 1.80 | 0.165 |
| Q5VYK3 | Proteasome-associated protein ECM29 homolog | 1 | 0.80 | 0.084 | 1.21 | 0.251 | 0.99 | 0.483 | **1.44** | **0.010** | 0.97 | 0.419 | 1.26 | 0.083 | 1.09 | 0.401 |
| Q6NZI2 | Polymerase I and transcript release factor | 1 | 0.61 | 0.198 | 0.67 | 0.231 | 0.89 | 0.409 | 1.07 | 0.449 | 0.51 | 0.136 | 0.36 | 0.061 | 0.87 | 0.401 |
| Q6P1L8 | 39S ribosomal protein L14. mitochondrial | 1 | 0.72 | 0.285 | 0.99 | 0.487 | 0.97 | 0.476 | 0.54 | 0.147 | 0.80 | 0.347 | 1.26 | 0.336 | 1.21 | 0.366 |
| Q6P2E9 | Enhancer of mRNA-decapping protein 4 | 1 | 0.40 | 0.134 | 0.76 | 0.337 | 0.88 | 0.421 | 0.98 | 0.485 | 0.92 | 0.450 | 0.66 | 0.280 | 0.68 | 0.296 |
| Q6UW68 | Transmembrane protein 205 | 1 | 0.83 | 0.417 | 0.97 | 0.437 | 0.80 | 0.326 | **0.73** | **0.043** | 0.69 | 0.215 | 0.79 | 0.102 | 0.85 | 0.335 |
| Q6ZRP7 | Sulfhydryl oxidase 2 | 1 | 1.62 | 0.309 | 1.26 | 0.406 | 1.42 | 0.357 | 2.81 | 0.128 | 1.05 | 0.482 | 0.92 | 0.467 | 1.99 | 0.235 |
| Q709C8 | Vacuolar protein sorting-associated protein 13C | 1 | 0.80 | 0.312 | 1.00 | 0.498 | 0.92 | 0.418 | 1.11 | 0.406 | 1.03 | 0.480 | 1.04 | 0.468 | 1.12 | 0.397 |
| Q71DI3 | Histone H3.2 | 1 | 0.56 | 0.096 | **0.48** | **0.050** | 0.61 | 0.130 | 0.60 | 0.123 | 0.59 | 0.118 | 0.52 | 0.074 | 0.68 | 0.194 |
| Q7Z6Z7 | E3 ubiquitin-protein ligase HUWE1 | 1 | 0.48 | 0.151 | 0.35 | 0.054 | 0.43 | 0.080 | 0.72 | 0.275 | 0.76 | 0.313 | 0.50 | 0.217 | 0.36 | 0.078 |
| Q86UY8 | 5'-nucleotidase domain-containing protein 3 | 1 | 1.61 | 0.236 | 1.16 | 0.381 | 1.23 | 0.329 | 1.11 | 0.414 | 1.41 | 0.263 | 1.27 | 0.313 | 0.88 | 0.397 |
| Q86Y56 | Dynein assembly factor 5. axonemal | 1 | 1.01 | 0.494 | 0.62 | 0.250 | 1.89 | 0.151 | 0.20 | 0.059 | 0.53 | 0.208 | 1.58 | 0.236 | 0.31 | 0.112 |
| Q8IVT2 | Mitotic interactor and substrate of PLK1 | 1 | 0.53 | 0.095 | 0.80 | 0.312 | **0.43** | **0.038** | 0.98 | 0.476 | 0.56 | 0.116 | 0.75 | 0.261 | 0.65 | 0.180 |
| Q8IWX8 | Calcium homeostasis endoplasmic reticulum protein | 1 | 1.26 | 0.417 | 0.90 | 0.444 | 1.15 | 0.394 | 1.09 | 0.437 | 0.74 | 0.366 | 1.45 | 0.229 | 1.07 | 0.465 |
| Q8IXB1 | DnaJ homolog subfamily C member 10 | 1 | 1.60 | 0.135 | 1.22 | 0.344 | **1.77** | **0.007** | 1.30 | 0.224 | 1.52 | 0.185 | 1.61 | 0.243 | **1.46** | **0.047** |
| Q8N2K0 | Monoacylglycerol lipase ABHD12 | 1 | 1.23 | 0.313 | 1.19 | 0.340 | 1.00 | 0.494 | 1.22 | 0.320 | 1.33 | 0.250 | 1.08 | 0.434 | 1.03 | 0.472 |
| Q8N4P2 | Tetratricopeptide repeat protein 30B | 1 | 2.17 | 0.125 | 1.15 | 0.423 | 1.35 | 0.334 | 1.55 | 0.263 | 1.40 | 0.317 | 1.48 | 0.289 | 1.09 | 0.455 |
| Q8NEV1 | Casein kinase II subunit alpha 3 | 1 | 0.90 | 0.407 | 1.03 | 0.475 | 0.64 | 0.159 | 1.09 | 0.420 | 0.66 | 0.185 | 0.72 | 0.236 | 0.81 | 0.320 |
| Q8TC12 | Retinol dehydrogenase 11 | 1 | 0.88 | 0.268 | 0.92 | 0.415 | 0.90 | 0.402 | 0.87 | 0.365 | 0.78 | 0.194 | 0.92 | 0.351 | 0.94 | 0.424 |
| Q8TCJ2 | Dolichyl-diphosphooligosaccharide--protein glycosyltransferase subunit STT3B | 1 | **0.39** | **0.037** | 0.66 | 0.194 | 0.44 | 0.050 | 0.44 | 0.054 | 0.77 | 0.292 | 0.52 | 0.098 | 0.50 | 0.091 |
| Q8TDB8 | Solute carrier family 2. facilitated glucose transporter member 14 | 1 | 2.55 | 0.072 | 1.42 | 0.298 | 1.37 | 0.315 | **4.85** | **0.005** | **4.28** | **0.010** | 1.95 | 0.153 | **4.14** | **0.012** |
| Q8TEX9 | Importin-4 | 1 | **1.74** | **0.012** | **2.16** | **0.047** | 1.63 | 0.058 | 1.29 | 0.344 | **2.02** | **0.019** | **2.30** | **0.047** | 1.16 | 0.353 |
| Q8WUY1 | Protein THEM6 | 1 | 1.07 | 0.460 | 1.19 | 0.390 | 0.75 | 0.320 | 0.76 | 0.327 | 1.26 | 0.353 | 1.05 | 0.471 | 0.93 | 0.453 |
| Q8WYA6 | Beta-catenin-like protein 1 | 1 | 1.78 | 0.160 | 1.65 | 0.191 | 0.95 | 0.463 | 1.22 | 0.371 | 1.08 | 0.451 | 1.65 | 0.195 | 1.43 | 0.277 |
| Q92499 | ATP-dependent RNA helicase DDX1 | 1 | 1.14 | 0.405 | 0.76 | 0.312 | 1.00 | 0.496 | 1.54 | 0.198 | 1.10 | 0.431 | 1.09 | 0.442 | 1.14 | 0.405 |
| Q92598 | Heat shock protein 105 kDa | 1 | 1.26 | 0.357 | 1.37 | 0.299 | **2.59** | **0.048** | 1.28 | 0.345 | 1.06 | 0.465 | 2.12 | 0.101 | 1.31 | 0.336 |
| Q92609 | TBC1 domain family member 5 | 1 | 0.66 | 0.213 | 0.93 | 0.425 | 0.69 | 0.206 | 0.59 | 0.222 | 0.54 | 0.118 | 0.68 | 0.218 | 0.86 | 0.391 |
| Q92643 | GPI-anchor transamidase | 1 | 1.05 | 0.424 | 1.07 | 0.412 | 0.93 | 0.433 | 1.08 | 0.421 | 1.04 | 0.471 | 1.18 | 0.280 | 0.88 | 0.279 |
| Q92743 | Serine protease HTRA1 | 1 | 2.23 | 0.082 | 1.10 | 0.438 | 1.42 | 0.276 | 1.28 | 0.339 | 1.67 | 0.192 | 1.90 | 0.135 | 1.15 | 0.415 |
| Q92896 | Golgi apparatus protein 1 | 1 | 1.29 | 0.206 | 0.92 | 0.291 | 1.14 | 0.211 | 0.88 | 0.355 | 1.07 | 0.357 | 1.59 | 0.073 | 0.89 | 0.338 |
| Q92973 | Transportin-1 | 1 | 1.23 | 0.407 | 1.45 | 0.207 | 1.26 | 0.288 | 1.07 | 0.454 | 1.31 | 0.334 | 1.47 | 0.128 | 1.11 | 0.428 |
| Q93050 | V-type proton ATPase 116 kDa subunit a isoform 1 | 1 | **3.96** | **0.001** | **2.50** | **0.015** | **4.51** | **0.000** | **2.29** | **0.026** | **4.55** | **0.000** | **3.01** | **0.005** | **4.10** | **0.000** |
| Q969H8 | Myeloid-derived growth factor | 1 | 0.94 | 0.432 | 0.98 | 0.474 | 1.00 | 0.495 | 0.96 | 0.462 | 0.83 | 0.323 | 0.73 | 0.291 | 0.89 | 0.391 |
| Q969X6 | Cirhin | 1 | 0.67 | 0.193 | 0.78 | 0.287 | 0.67 | 0.186 | 0.86 | 0.367 | 1.04 | 0.468 | 0.85 | 0.363 | 0.67 | 0.197 |
| Q96F07 | Cytoplasmic FMR1-interacting protein 2 | 1 | 0.64 | 0.095 | 0.95 | 0.415 | 0.87 | 0.387 | 1.21 | 0.173 | 0.69 | 0.187 | 0.95 | 0.451 | 0.95 | 0.396 |
| Q96FQ6 | Protein S100-A16 | 1 | 2.51 | 0.130 | 2.51 | 0.127 | 1.75 | 0.248 | 2.37 | 0.143 | 3.07 | 0.082 | 1.19 | 0.422 | 3.14 | 0.079 |
| Q96HE7 | ERO1-like protein alpha | 1 | 1.13 | 0.293 | 1.02 | 0.486 | 1.23 | 0.285 | **1.57** | **0.015** | 1.39 | 0.190 | **2.02** | **0.003** | 1.09 | 0.341 |
| Q96IU4 | Alpha/beta hydrolase domain-containing protein 14B | 1 | 0.88 | 0.396 | 1.00 | 0.497 | 0.79 | 0.317 | 1.50 | 0.201 | 0.76 | 0.299 | 0.90 | 0.418 | 0.75 | 0.289 |
| Q96J01 | THO complex subunit 3 | 1 | **0.62** | **0.020** | **0.71** | **0.035** | 0.88 | 0.369 | 0.77 | 0.075 | 0.98 | 0.451 | 0.82 | 0.147 | **0.60** | **0.018** |
| Q96JQ2 | Calmin | 1 | 0.89 | 0.392 | 0.69 | 0.198 | 0.78 | 0.281 | 1.04 | 0.471 | 0.80 | 0.341 | 0.88 | 0.380 | 0.78 | 0.292 |
| Q96KA5 | Cleft lip and palate transmembrane protein 1-like protein | 1 | 1.09 | 0.449 | 1.18 | 0.371 | 1.18 | 0.295 | 1.00 | 0.495 | 1.37 | 0.277 | 1.37 | 0.221 | 1.07 | 0.466 |
| Q96KC8 | DnaJ homolog subfamily C member 1 | 1 | 1.00 | 0.499 | 0.76 | 0.225 | 0.61 | 0.098 | 0.72 | 0.199 | 0.70 | 0.165 | 0.46 | 0.081 | 0.61 | 0.081 |
| Q96P70 | Importin-9 | 1 | 1.37 | 0.270 | 1.12 | 0.289 | 1.05 | 0.429 | 1.42 | 0.141 | 1.18 | 0.199 | 1.18 | 0.205 | 1.50 | 0.107 |
| Q96PZ0 | Pseudouridylate synthase 7 homolog | 1 | 0.88 | 0.406 | 0.70 | 0.252 | 0.89 | 0.409 | 1.20 | 0.363 | 1.00 | 0.494 | 0.74 | 0.286 | 0.99 | 0.488 |
| Q96RP9 | Elongation factor G. mitochondrial | 1 | 1.06 | 0.463 | 0.36 | 0.059 | 0.84 | 0.377 | 1.04 | 0.475 | 0.74 | 0.302 | 0.52 | 0.150 | 0.28 | 0.053 |
| Q96SQ9 | Cytochrome P450 2S1 | 1 | 0.50 | 0.075 | 0.86 | 0.370 | 0.84 | 0.341 | 0.78 | 0.283 | **0.34** | **0.035** | 0.62 | 0.147 | 0.66 | 0.182 |
| Q96SU4 | Oxysterol-binding protein-related protein 9 | 1 | 1.26 | 0.376 | 1.01 | 0.496 | 1.52 | 0.270 | 1.18 | 0.412 | 1.76 | 0.209 | 0.87 | 0.423 | 1.25 | 0.383 |
| Q99460 | 26S proteasome non-ATPase regulatory subunit 1 | 1 | 1.94 | 0.052 | **1.93** | **0.039** | 1.69 | 0.110 | 1.10 | 0.403 | 1.89 | 0.095 | 1.66 | 0.055 | 1.64 | 0.154 |
| Q99497 | Protein deglycase DJ-1 | 1 | 0.84 | 0.382 | 0.41 | 0.085 | 0.58 | 0.181 | 0.66 | 0.244 | 0.57 | 0.187 | 0.73 | 0.297 | 0.85 | 0.394 |
| Q99536 | Synaptic vesicle membrane protein VAT-1 homolog | 1 | 0.91 | 0.313 | **0.62** | **0.024** | 1.02 | 0.472 | 0.79 | 0.267 | 0.95 | 0.369 | 0.73 | 0.251 | 1.04 | 0.443 |
| Q9BQA1 | Methylosome protein 50 | 1 | 0.93 | 0.439 | 1.21 | 0.349 | 1.07 | 0.444 | 1.67 | 0.138 | 1.09 | 0.432 | 1.73 | 0.126 | 0.90 | 0.418 |
| Q9BRA2 | Thioredoxin domain-containing protein 17 | 1 | **1.95** | **0.022** | 1.36 | 0.164 | **2.01** | **0.000** | **1.35** | **0.043** | **1.88** | **0.025** | **1.74** | **0.001** | **2.00** | **0.006** |
| Q9BRJ6 | Uncharacterized protein C7orf50 | 1 | 0.81 | 0.339 | 0.52 | 0.111 | 0.65 | 0.197 | 0.85 | 0.369 | 0.53 | 0.125 | 0.80 | 0.333 | 1.15 | 0.392 |
| Q9BRT6 | Protein LLP homolog | 1 | 1.05 | 0.463 | 0.89 | 0.408 | 1.17 | 0.377 | 1.16 | 0.382 | 1.01 | 0.497 | 0.62 | 0.183 | 1.22 | 0.350 |
| Q9BSC4 | Nucleolar protein 10 | 1 | 1.21 | 0.375 | 1.06 | 0.465 | 0.91 | 0.433 | 1.07 | 0.454 | 1.74 | 0.169 | 0.89 | 0.424 | 0.61 | 0.229 |
| Q9BSJ8 | Extended synaptotagmin-1 | 1 | 1.63 | 0.168 | 1.28 | 0.317 | 1.43 | 0.237 | 1.15 | 0.397 | 1.62 | 0.173 | 1.47 | 0.226 | 1.59 | 0.184 |
| Q9BT22 | Chitobiosyldiphosphodolichol beta-mannosyltransferase | 1 | 1.05 | 0.471 | 1.10 | 0.445 | 0.82 | 0.382 | 0.72 | 0.312 | 1.37 | 0.315 | 1.25 | 0.366 | 1.18 | 0.406 |
| Q9BTU6 | Phosphatidylinositol 4-kinase type 2-alpha | 1 | **1.99** | **0.045** | 1.54 | 0.152 | 1.91 | 0.092 | 1.42 | 0.100 | 2.55 | 0.052 | **2.21** | **0.001** | 1.89 | 0.085 |
| Q9BUN8 | Derlin-1 | 1 | 0.84 | 0.317 | 1.06 | 0.440 | 1.05 | 0.450 | 1.04 | 0.457 | 0.70 | 0.207 | 0.91 | 0.469 | 0.91 | 0.451 |
| Q9BVG4 | Protein PBDC1 | 1 | 1.05 | 0.463 | 1.66 | 0.131 | 0.95 | 0.449 | 1.09 | 0.433 | 1.41 | 0.232 | 1.47 | 0.205 | 1.45 | 0.227 |
| Q9BVG8 | Kinesin-like protein KIFC3 | 1 | 0.86 | 0.370 | 1.29 | 0.265 | 0.62 | 0.166 | 1.33 | 0.239 | 0.70 | 0.208 | 1.06 | 0.448 | 0.83 | 0.328 |
| Q9BWJ5 | Splicing factor 3B subunit 5 | 1 | **1.69** | **0.046** | **1.59** | **0.030** | 0.81 | 0.356 | **1.95** | **0.049** | 1.61 | 0.118 | **2.14** | **0.001** | 0.84 | 0.385 |
| Q9BZE4 | Nucleolar GTP-binding protein 1 | 1 | 1.37 | 0.248 | 0.54 | 0.101 | 0.97 | 0.474 | 1.00 | 0.493 | 0.89 | 0.402 | 0.89 | 0.399 | 0.65 | 0.186 |
| Q9GZP4 | PITH domain-containing protein 1 | 1 | 1.21 | 0.343 | 1.07 | 0.446 | 1.42 | 0.222 | 0.80 | 0.318 | 0.86 | 0.371 | 0.87 | 0.379 | 1.16 | 0.376 |
| Q9GZS3 | WD repeat-containing protein 61 | 1 | 0.77 | 0.337 | 1.25 | 0.352 | 0.85 | 0.388 | 1.35 | 0.303 | 1.44 | 0.271 | 1.44 | 0.267 | 0.95 | 0.465 |
| Q9GZT3 | SRA stem-loop-interacting RNA-binding protein. mitochondrial | 1 | **4.31** | **0.015** | 2.75 | 0.070 | 3.04 | 0.051 | 1.78 | 0.209 | **3.65** | **0.029** | **4.21** | **0.017** | **3.46** | **0.036** |
| Q9H0A0 | N-acetyltransferase 10 | 1 | 0.91 | 0.394 | 0.75 | 0.200 | 0.88 | 0.358 | 0.82 | 0.301 | 0.79 | 0.244 | 0.74 | 0.193 | 0.91 | 0.415 |
| Q9H0C8 | Integrin-linked kinase-associated serine/threonine phosphatase 2C | 1 | 2.09 | 0.151 | 1.67 | 0.231 | 2.08 | 0.062 | 1.90 | 0.052 | 1.94 | 0.159 | **2.23** | **0.021** | 1.79 | 0.093 |
| Q9H0D6 | 5'-3' exoribonuclease 2 | 1 | 0.91 | 0.418 | 0.97 | 0.483 | 0.90 | 0.410 | 1.17 | 0.385 | 1.41 | 0.349 | 1.25 | 0.317 | 0.96 | 0.470 |
| Q9H0V9 | VIP36-like protein | 1 | 1.47 | 0.274 | 1.56 | 0.239 | 2.33 | 0.081 | 2.38 | 0.078 | 2.72 | 0.051 | 1.71 | 0.199 | 2.18 | 0.107 |
| Q9H2M9 | Rab3 GTPase-activating protein non-catalytic subunit | 1 | 1.93 | 0.120 | 2.19 | 0.076 | 1.80 | 0.144 | 1.75 | 0.157 | 1.57 | 0.215 | 1.56 | 0.217 | 1.47 | 0.253 |
| Q9H2V7 | Protein spinster homolog 1 | 1 | 0.66 | 0.275 | 0.90 | 0.430 | 1.32 | 0.326 | 0.73 | 0.317 | 1.10 | 0.441 | 1.29 | 0.347 | 1.51 | 0.259 |
| Q9H3U1 | Protein unc-45 homolog A | 1 | 0.87 | 0.407 | 0.60 | 0.202 | 1.14 | 0.410 | 0.88 | 0.414 | 1.04 | 0.474 | 0.54 | 0.170 | 1.09 | 0.442 |
| Q9H6V9 | UPF0554 protein C2orf43 | 1 | 1.30 | 0.342 | **2.96** | **0.034** | 2.11 | 0.108 | 1.66 | 0.205 | 1.95 | 0.141 | 2.12 | 0.109 | 1.67 | 0.209 |
| Q9H7N4 | Splicing factor. arginine/serine-rich 19 | 1 | 1.08 | 0.331 | 0.98 | 0.441 | 1.06 | 0.406 | 0.95 | 0.375 | 0.88 | 0.219 | 0.86 | 0.326 | 0.89 | 0.239 |
| Q9H845 | Acyl-CoA dehydrogenase family member 9. mitochondrial | 1 | 0.93 | 0.444 | 0.58 | 0.172 | 0.68 | 0.242 | 0.36 | 0.056 | 1.35 | 0.290 | 0.52 | 0.142 | 0.53 | 0.153 |
| Q9H9T3 | Elongator complex protein 3 | 1 | 0.75 | 0.130 | 0.95 | 0.445 | 0.76 | 0.262 | 0.87 | 0.363 | 1.04 | 0.440 | 0.94 | 0.418 | 1.02 | 0.477 |
| Q9HB07 | UPF0160 protein MYG1. mitochondrial | 1 | 0.97 | 0.480 | 1.07 | 0.433 | 0.83 | 0.380 | 1.19 | 0.358 | 1.21 | 0.349 | 1.13 | 0.353 | 1.19 | 0.359 |
| Q9HB71 | Calcyclin-binding protein | 1 | 2.34 | 0.213 | 2.28 | 0.218 | 2.58 | 0.182 | 3.65 | 0.105 | 0.90 | 0.466 | 3.24 | 0.130 | 2.58 | 0.188 |
| Q9HC38 | Glyoxalase domain-containing protein 4 | 1 | 0.68 | 0.288 | 0.66 | 0.068 | 0.65 | 0.072 | 0.73 | 0.152 | **0.52** | **0.043** | **0.75** | **0.043** | 0.63 | 0.116 |
| Q9HD15 | Steroid receptor RNA activator 1 | 1 | **1.42** | **0.027** | **1.41** | **0.008** | **1.38** | **0.038** | **1.55** | **0.041** | 1.06 | 0.357 | **1.51** | **0.002** | 1.18 | 0.154 |
| Q9HD45 | Transmembrane 9 superfamily member 3 | 1 | 3.02 | 0.056 | 1.63 | 0.248 | 2.48 | 0.096 | 1.64 | 0.246 | 0.43 | 0.197 | **3.20** | **0.047** | 2.08 | 0.156 |
| Q9NPD3 | Exosome complex component RRP41 | 1 | 1.25 | 0.178 | 1.33 | 0.099 | 1.10 | 0.401 | 1.26 | 0.071 | 1.15 | 0.192 | **1.44** | **0.041** | 1.06 | 0.385 |
| Q9NPD8 | Ubiquitin-conjugating enzyme E2 T | 1 | 0.42 | 0.066 | 0.49 | 0.092 | **0.11** | **0.003** | **0.13** | **0.005** | 0.50 | 0.109 | 0.62 | 0.185 | **0.32** | **0.037** |
| Q9NPL8 | Complex I assembly factor TIMMDC1. mitochondrial | 1 | 0.73 | 0.299 | 0.85 | 0.358 | 0.69 | 0.243 | 0.89 | 0.399 | 1.10 | 0.426 | 0.62 | 0.206 | 0.73 | 0.268 |
| Q9NQR4 | Omega-amidase NIT2 | 1 | 0.90 | 0.329 | 0.98 | 0.457 | 0.82 | 0.318 | 0.92 | 0.422 | 0.84 | 0.256 | 0.83 | 0.189 | 0.98 | 0.431 |
| Q9NR46 | Endophilin-B2 | 1 | 1.67 | 0.185 | 1.14 | 0.415 | 1.62 | 0.195 | 1.10 | 0.435 | 1.11 | 0.435 | 1.56 | 0.218 | 1.30 | 0.332 |
| Q9NRX2 | 39S ribosomal protein L17. mitochondrial | 1 | 0.86 | 0.391 | 0.82 | 0.359 | 1.03 | 0.477 | **0.33** | **0.043** | 0.69 | 0.260 | 1.12 | 0.418 | 0.73 | 0.290 |
| Q9NTK5 | Obg-like ATPase 1 | 1 | 1.27 | 0.322 | 1.18 | 0.371 | 0.91 | 0.424 | 0.88 | 0.403 | 0.93 | 0.445 | 0.91 | 0.425 | 1.27 | 0.321 |
| Q9NUJ1 |  | 1 | 1.85 | 0.141 | 1.66 | 0.187 | 2.30 | 0.065 | 1.55 | 0.223 | 2.06 | 0.102 | 1.66 | 0.190 | 1.43 | 0.280 |
| Q9NUQ9 | Protein FAM49B | 1 | 1.64 | 0.075 | **1.71** | **0.009** | **1.43** | **0.018** | **1.42** | **0.005** | 1.47 | 0.065 | 1.37 | 0.076 | **1.41** | **0.010** |
| Q9NV31 | U3 small nucleolar ribonucleoprotein protein IMP3 | 1 | 1.37 | 0.245 | 1.65 | 0.291 | **1.66** | **0.035** | **1.75** | **0.024** | 1.73 | 0.166 | 1.51 | 0.307 | 1.66 | 0.058 |
| Q9NVP1 | ATP-dependent RNA helicase DDX18 | 1 | 1.60 | 0.303 | 1.53 | 0.293 | 1.40 | 0.352 | 1.09 | 0.450 | 2.14 | 0.059 | 1.45 | 0.254 | 1.44 | 0.313 |
| Q9NVX2 | Notchless protein homolog 1 | 1 | 0.85 | 0.383 | 0.88 | 0.414 | 0.75 | 0.254 | 1.07 | 0.443 | 1.07 | 0.443 | 0.95 | 0.451 | 0.83 | 0.336 |
| Q9NX40 | OCIA domain-containing protein 1 | 1 | 1.45 | 0.097 | 1.04 | 0.474 | 1.23 | 0.360 | 1.40 | 0.214 | 1.33 | 0.242 | 1.86 | 0.119 | 1.34 | 0.288 |
| Q9NY33 | Dipeptidyl peptidase 3 | 1 | 1.01 | 0.497 | 0.63 | 0.221 | 1.01 | 0.499 | 0.83 | 0.372 | 0.90 | 0.428 | 0.58 | 0.196 | 1.47 | 0.250 |
| Q9NZ01 | Very-long-chain enoyl-CoA reductase | 1 | **1.77** | **0.001** | **1.83** | **0.001** | 1.21 | 0.289 | 1.13 | 0.421 | **2.34** | **0.000** | **1.52** | **0.046** | 1.21 | 0.355 |
| Q9NZB2 | Constitutive coactivator of PPAR-gamma-like protein 1 | 1 | 0.59 | 0.134 | 0.86 | 0.369 | 0.59 | 0.124 | 0.97 | 0.472 | 1.63 | 0.137 | 0.95 | 0.453 | 1.27 | 0.300 |
| Q9NZI8 | Insulin-like growth factor 2 mRNA-binding protein 1 | 1 | 0.87 | 0.328 | 0.98 | 0.458 | 1.06 | 0.381 | 1.05 | 0.413 | 0.79 | 0.127 | 0.89 | 0.285 | **0.65** | **0.042** |
| Q9NZJ7 | Mitochondrial carrier homolog 1 | 1 | 0.73 | 0.333 | 0.66 | 0.281 | 0.51 | 0.180 | 0.51 | 0.189 | 0.65 | 0.286 | 1.23 | 0.380 | 1.11 | 0.442 |
| Q9NZL4 | Hsp70-binding protein 1 | 1 | 0.59 | 0.274 | 2.04 | 0.158 | 1.51 | 0.284 | 1.98 | 0.170 | 1.83 | 0.204 | 1.97 | 0.173 | 1.43 | 0.321 |
| Q9NZR1 | Tropomodulin-2 | 1 | **0.64** | **0.047** | 0.77 | 0.066 | **0.67** | **0.001** | 0.83 | 0.164 | **0.48** | **0.000** | **0.70** | **0.003** | **0.65** | **0.000** |
| Q9P287 | BRCA2 and CDKN1A-interacting protein | 1 | 1.71 | 0.190 | 1.34 | 0.319 | 1.72 | 0.185 | 0.81 | 0.371 | 1.33 | 0.326 | 2.13 | 0.104 | 1.36 | 0.316 |
| Q9P2R3 | Rabankyrin-5 | 1 | 2.02 | 0.153 | 1.75 | 0.185 | 1.38 | 0.283 | 1.18 | 0.389 | 1.38 | 0.291 | 1.77 | 0.154 | 1.93 | 0.150 |
| Q9UBP6 | tRNA (guanine-N(7)-)-methyltransferase | 1 | 1.16 | 0.397 | 1.97 | 0.100 | 0.95 | 0.463 | 1.77 | 0.142 | 1.37 | 0.342 | 1.26 | 0.343 | 1.28 | 0.334 |
| Q9UFN0 | Protein NipSnap homolog 3A | 1 | **2.21** | **0.042** | **2.27** | **0.035** | 1.96 | 0.071 | 1.57 | 0.169 | 1.87 | 0.091 | **2.15** | **0.048** | **2.39** | **0.030** |
| Q9UHB6 | LIM domain and actin-binding protein 1 | 1 | **4.13** | **0.001** | 1.18 | 0.380 | 1.99 | 0.060 | 1.42 | 0.282 | **3.15** | **0.005** | 1.53 | 0.187 | **3.13** | **0.005** |
| Q9UIA9 | Exportin-7 | 1 | 1.43 | 0.331 | 1.59 | 0.279 | 2.82 | 0.085 | 1.75 | 0.240 | 2.02 | 0.188 | 1.32 | 0.368 | 1.10 | 0.457 |
| Q9UIL1 | Short coiled-coil protein | 1 | 0.65 | 0.332 | 0.73 | 0.364 | 2.05 | 0.184 | 1.12 | 0.450 | 0.66 | 0.338 | 1.18 | 0.426 | 1.19 | 0.424 |
| Q9UJS0 | Calcium-binding mitochondrial carrier protein Aralar2 | 1 | 1.35 | 0.316 | 1.67 | 0.195 | 2.29 | 0.078 | 0.65 | 0.258 | 1.46 | 0.273 | 2.39 | 0.070 | 1.94 | 0.137 |
| Q9UJU6 | Drebrin-like protein | 1 | 0.71 | 0.240 | 0.50 | 0.079 | 0.97 | 0.473 | 0.93 | 0.439 | 0.73 | 0.256 | 1.13 | 0.396 | 0.57 | 0.134 |
| Q9UJZ1 | Stomatin-like protein 2. mitochondrial | 1 | 1.38 | 0.328 | 0.29 | 0.106 | 2.56 | 0.080 | 1.16 | 0.420 | 0.77 | 0.370 | 2.94 | 0.054 | 0.78 | 0.381 |
| Q9UK76 | Hematological and neurological expressed 1 protein | 1 | 2.50 | 0.101 | 1.16 | 0.424 | 0.95 | 0.472 | 0.83 | 0.403 | 0.91 | 0.450 | 0.62 | 0.290 | 1.85 | 0.204 |
| Q9UL25 | Ras-related protein Rab-21 | 1 | 0.94 | 0.441 | 1.35 | 0.227 | 0.93 | 0.423 | 1.01 | 0.493 | 1.04 | 0.476 | 0.96 | 0.455 | 1.27 | 0.286 |
| Q9UNN8 | Endothelial protein C receptor | 1 | 0.64 | 0.108 | 0.74 | 0.193 | 0.57 | 0.060 | 0.89 | 0.360 | 0.66 | 0.229 | 0.70 | 0.163 | 0.62 | 0.097 |
| Q9UPT5 | Exocyst complex component 7 | 1 | 1.82 | 0.131 | 1.55 | 0.207 | 1.50 | 0.223 | 1.19 | 0.379 | 1.13 | 0.415 | 1.35 | 0.291 | 1.31 | 0.314 |
| Q9UPY5 | Cystine/glutamate transporter | 1 | 0.55 | 0.200 | 1.25 | 0.361 | 0.57 | 0.203 | 1.28 | 0.344 | 0.95 | 0.469 | 1.38 | 0.301 | 1.21 | 0.383 |
| Q9Y230 | RuvB-like 2 | 1 | 0.80 | 0.134 | 1.13 | 0.363 | 1.39 | 0.051 | 0.75 | 0.057 | 1.36 | 0.074 | 0.96 | 0.406 | 1.26 | 0.180 |
| Q9Y237 | Peptidyl-prolyl cis-trans isomerase NIMA-interacting 4 | 1 | 2.18 | 0.152 | 1.76 | 0.228 | 2.38 | 0.120 | 1.13 | 0.443 | 2.52 | 0.109 | 1.61 | 0.270 | 2.22 | 0.148 |
| Q9Y262 | Eukaryotic translation initiation factor 3 subunit L | 1 | 2.18 | 0.154 | **3.67** | **0.038** | 2.20 | 0.147 | 2.07 | 0.170 | 2.40 | 0.126 | 2.83 | 0.083 | 1.93 | 0.201 |
| Q9Y285 | Phenylalanine--tRNA ligase alpha subunit | 1 | 0.95 | 0.402 | 1.35 | 0.199 | 1.16 | 0.278 | 1.21 | 0.204 | 0.87 | 0.284 | 1.14 | 0.355 | 1.12 | 0.408 |
| Q9Y2C4 | Nuclease EXOG. mitochondrial | 1 | 1.30 | 0.320 | 1.07 | 0.456 | 1.06 | 0.462 | 1.44 | 0.249 | 0.80 | 0.351 | 1.35 | 0.292 | 0.99 | 0.491 |
| Q9Y2L1 | Exosome complex exonuclease RRP44 | 1 | 1.30 | 0.198 | 0.92 | 0.331 | 0.90 | 0.434 | **0.67** | **0.036** | 1.14 | 0.268 | 0.83 | 0.344 | 1.11 | 0.322 |
| Q9Y2R9 | 28S ribosomal protein S7. mitochondrial | 1 | 0.81 | 0.181 | 0.81 | 0.334 | 0.79 | 0.213 | 0.79 | 0.203 | **0.68** | **0.028** | **0.62** | **0.016** | 0.62 | 0.073 |
| Q9Y2V2 | Calcium-regulated heat stable protein 1 | 1 | **2.92** | **0.000** | 2.03 | 0.071 | **2.85** | **0.001** | **1.80** | **0.002** | **2.66** | **0.000** | **1.80** | **0.028** | **2.47** | **0.013** |
| Q9Y2V7 | Conserved oligomeric Golgi complex subunit 6 | 1 | **2.50** | **0.013** | **2.67** | **0.008** | **3.18** | **0.002** | 1.29 | 0.271 | **2.93** | **0.004** | **2.41** | **0.016** | **2.73** | **0.007** |
| Q9Y333 | U6 snRNA-associated Sm-like protein LSm2 | 1 | 1.22 | 0.314 | 1.20 | 0.395 | 1.17 | 0.366 | 1.31 | 0.301 | 1.53 | 0.149 | 1.56 | 0.274 | 1.29 | 0.304 |
| Q9Y383 | Putative RNA-binding protein Luc7-like 2 | 1 | 1.39 | 0.263 | **2.61** | **0.024** | 0.95 | 0.471 | 2.02 | 0.078 | **2.69** | **0.021** | 2.20 | 0.056 | 1.74 | 0.141 |
| Q9Y3A4 | Ribosomal RNA-processing protein 7 homolog A | 1 | 1.40 | 0.185 | 1.25 | 0.136 | 1.44 | 0.092 | 1.33 | 0.189 | 1.53 | 0.074 | **1.64** | **0.029** | 0.97 | 0.469 |
| Q9Y3E5 | Peptidyl-tRNA hydrolase 2. mitochondrial | 1 | 1.27 | 0.151 | 1.18 | 0.305 | 1.16 | 0.207 | 1.21 | 0.118 | 1.34 | 0.069 | 1.41 | 0.067 | 1.18 | 0.390 |
| Q9Y3F4 | Serine-threonine kinase receptor-associated protein | 1 | 0.83 | 0.300 | 0.72 | 0.178 | **0.54** | **0.048** | 0.87 | 0.345 | 0.82 | 0.294 | 0.65 | 0.173 | 0.87 | 0.348 |
| Q9Y3T9 | Nucleolar complex protein 2 homolog | 1 | **1.59** | **0.042** | **1.54** | **0.018** | **1.65** | **0.028** | 1.41 | 0.050 | **2.21** | **0.000** | **1.96** | **0.001** | **1.59** | **0.000** |
| Q9Y4R8 | Telomere length regulation protein TEL2 homolog | 1 | 1.18 | 0.374 | 0.75 | 0.329 | 1.19 | 0.359 | 0.93 | 0.458 | 1.24 | 0.334 | 1.58 | 0.291 | 0.76 | 0.303 |
| Q9Y570 | Protein phosphatase methylesterase 1 | 1 | 1.63 | 0.101 | 1.11 | 0.367 | 1.25 | 0.241 | 1.28 | 0.218 | 1.53 | 0.160 | 1.25 | 0.232 | 1.14 | 0.363 |
| Q9Y5K8 | V-type proton ATPase subunit D | 1 | 1.00 | 0.496 | 0.53 | 0.172 | 0.75 | 0.320 | 0.48 | 0.143 | 0.61 | 0.234 | 1.09 | 0.447 | 0.54 | 0.197 |
| Q9Y5L4 | Mitochondrial import inner membrane translocase subunit Tim13 | 1 | 0.93 | 0.444 | 0.65 | 0.218 | 0.70 | 0.253 | 0.88 | 0.402 | 0.92 | 0.437 | 0.67 | 0.240 | 0.96 | 0.468 |
| Q9Y617 | Phosphoserine aminotransferase | 1 | 0.86 | 0.158 | **1.42** | **0.014** | 0.98 | 0.427 | 1.00 | 0.487 | 1.15 | 0.182 | 1.12 | 0.301 | 1.24 | 0.233 |
| Q9Y639 | Neuroplastin | 1 | 2.15 | 0.072 | 1.45 | 0.295 | 1.45 | 0.245 | 1.77 | 0.163 | 1.04 | 0.474 | 1.43 | 0.258 | 0.86 | 0.396 |
| Q9Y678 | Coatomer subunit gamma-1 | 1 | 1.35 | 0.127 | **1.45** | **0.014** | 1.41 | 0.072 | **1.25** | **0.046** | **1.42** | **0.046** | 1.31 | 0.129 | 1.36 | 0.124 |
| Q9Y679 | Ancient ubiquitous protein 1 | 1 | 1.12 | 0.287 | 0.90 | 0.307 | 1.02 | 0.475 | 1.06 | 0.386 | 1.12 | 0.256 | 0.74 | 0.152 | 1.15 | 0.249 |
| Q9Y6Y8 | SEC23-interacting protein | 1 | 1.32 | 0.091 | **1.36** | **0.034** | **1.57** | **0.003** | **1.51** | **0.006** | **1.50** | **0.004** | **1.91** | **0.000** | **1.43** | **0.024** |

**Table S2**

| **Gene ontology analysis** | **TSA**  **Normoxia** | | **NAM Normoxia** | | **TSA + NAM Normoxia** | | **Hypoxia** | | **TSA**  **Hypoxia** | | **NAM**  **Hypoxia** | | **TSA + NAM Hypoxia** | |
| --- | --- | --- | --- | --- | --- | --- | --- | --- | --- | --- | --- | --- | --- | --- |
| **Biological process term** | **Percentage of proteins** | **p-value** | **Percentage of proteins** | **p-value** | **Percentage of proteins** | **p-value** | **Percentage of proteins** | **p-value** | **Percentage of proteins** | **p-value** | **Percentage of proteins** | **p-value** | **Percentage of proteins** | **p-value** |
| translation | **26** | **<0.001** | **30** | **<0.001** | **26** | **<0.001** | **26** | **<0.001** | **23** | **<0.001** | **33** | **<0.001** | **25** | **<0.001** |
| translational elongation | **21** | **<0.001** | **22** | **<0.001** | **20** | **<0.001** | **17** | **<0.001** | **21** | **<0.001** | **28** | **<0.001** | **22** | **<0.001** |
| mRNA metabolic process | **15** | **0.001** | **15** | **0.033** | **16** | **<0.001** |  |  | **14** | **0.004** | **14** | **0.002** | **22** | **<0.001** |
| mRNA processing | **15** | **<0.001** | **15** | **0.023** | **14** | **0.001** |  |  | **14** | **0.002** | **12** | **0.005** | **19** | **<0.001** |
| RNA processing | **21** | **<0.001** | **19** | **0.019** | **20** | **<0.001** |  |  | **18** | **0.001** | **20** | **<0.001** | **28** | **<0.001** |
| RNA splicing | **17** | **<0.001** | **15** | **0.017** | **14** | **<0.001** |  |  | **14** | **0.001** | **12** | **0.003** | **19** | **<0.001** |
| nuclear mRNA splicing. via spliceosome | **9** | **0.012** | 6 | 0.072 | **10** | **0.002** |  |  | **11** | **0.001** | **8** | **0.015** | **11** | **0.006** |
| RNA splicing. via transesterification reactions | **9** | **0.012** | 6 | 0.072 | **10** | **0.002** |  |  | **11** | **0.001** | **8** | **0.015** | **11** | **0.006** |
| RNA splicing. via transesterification reactions with bulged adenosine as nucleophile | **9** | **0.012** | 6 | 0.072 | **10** | **0.002** |  |  | **11** | **0.001** | **8** | **0.015** | **11** | **0.006** |
| ribonucleoprotein complex biogenesis | **9** | **0.018** |  |  | **8** | **0.022** |  |  | 7 | 0.091 | **8** | **0.023** | 8 | 0.071 |
| ribosomal large subunit biogenesis | **4** | **0.031** |  |  |  |  |  |  |  |  | **4** | 0.033 | **8** | **<0.001** |
| ribosome biogenesis | **9** | **0.006** |  |  | 6 | 0.062 |  |  |  |  | 6 | 0.065 | **8** | **0.036** |
| generation of precursor metabolites and energy | **14** | **<0.001** | **13** | **0.002** | **11** | **0.003** | **15** | **0.005** | **17** | **<0.001** |  |  | **17** | **<0.001** |
| intracellular transport | **14** | **0.012** | **19** | **<0.001** | **20** | **0.000** | 17 | 0.079 | **18** | **<0.001** | **11** | **0.046** | **15** | **0.015** |
| nuclear transport | **7** | **0.023** | **11** | **0.001** | **8** | **0.005** |  |  | **10** | **<0.001** | **7** | **0.025** | **9** | **0.013** |
| nucleocytoplasmic transport | **7** | **0.022** | **11** | **0.001** | **8** | **0.005** |  |  | **10** | **<0.001** | **7** | **0.024** | **9** | **0.012** |
| nitrogen compound biosynthetic process | **9** | **0.036** |  |  | **11** | **0.003** |  |  |  |  | **10** | **0.009** | **11** | **0.018** |
| glutamine family amino acid metabolic process | **5** | **0.017** |  |  | **5** | **0.022** |  |  |  |  | **5** | **0.018** |  |  |
| proline biosynthetic process | **3** | **0.027** |  |  | **3** | **0.031** |  |  |  |  | **3** | **0.028** |  |  |
| proline metabolic process | **3** | **0.038** |  |  | **3** | **0.043** |  |  |  |  | **3** | **0.039** |  |  |
| response to oxidative stress | **7** | **0.025** |  |  | **6** | **0.036** |  |  |  |  | **8** | **0.004** |  |  |
| glucose metabolic process | **7** | **0.021** |  |  |  |  | **9** | **0.045** | **6** | **0.036** |  |  | 6 | 0.082 |
| protein complex assembly | **10** | **0.044** | 11 | 0.056 | 9 | 0.073 |  |  | **13** | **0.003** | **11** | **0.015** |  |  |
| protein complex biogenesis | **10** | **0.044** | 11 | 0.056 | 9 | 0.073 |  |  | **13** | **0.003** | **11** | **0.015** |  |  |
| glucose catabolic process | **5** | **0.021** |  |  |  |  |  |  | **4** | **0.031** |  |  |  |  |
| glycolysis | **5** | **0.014** |  |  |  |  | 6 | 0.099 | **4** | **0.021** |  |  |  |  |
| hexose catabolic process | **5** | **0.029** |  |  |  |  |  |  | **4** | **0.042** |  |  |  |  |
| lipid biosynthetic process | **9** | **0.035** |  |  |  |  |  |  | **10** | **0.004** |  |  |  |  |
| monosaccharide catabolic process | **5** | **0.030** |  |  |  |  |  |  | **4** | **0.044** |  |  |  |  |
| steroid biosynthetic process | **5** | **0.042** |  |  |  |  |  |  | **6** | **0.008** |  |  |  |  |
| pyrimidine nucleoside triphosphate biosynthetic process | **3** | **0.045** |  |  | 3 | 0.052 |  |  |  |  | **3** | 0.047 |  |  |
| alcohol catabolic process | **5** | **0.039** |  |  |  |  |  |  | 4 | 0.056 |  |  |  |  |
| cellular carbohydrate catabolic process | **5** | **0.042** |  |  |  |  |  |  | 4 | 0.061 |  |  |  |  |
| hexose metabolic process | **7** | **0.037** |  |  |  |  | 9 | 0.067 | 6 | 0.063 |  |  |  |  |
| oxidation reduction | **12** | **0.035** |  |  |  |  |  |  |  |  |  |  |  |  |
| anaphase-promoting complex-dependent proteasomal ubiquitin-dependent protein catabolic process |  |  | **6** | **0.015** | **6** | **0.020** |  |  | **7** | **0.014** | **16** | **<0.001** |  |  |
| negative regulation of cellular protein metabolic process |  |  | **9** | **0.015** | **12** | **<0.001** |  |  | **9** | **0.014** | **18** | **<0.001** | 8 | 0.071 |
| negative regulation of ligase activity |  |  | **6** | **0.016** | **6** | **0.021** |  |  | **7** | **0.015** | **16** | **<0.001** |  |  |
| negative regulation of protein metabolic process |  |  | **9** | **0.016** | **12** | **<0.001** |  |  | **9** | **0.015** | **18** | **<0.001** | 8 | 0.076 |
| negative regulation of protein modification process |  |  | **9** | **0.005** | **12** | **<0.001** |  |  | **9** | **0.005** | **18** | **<0.001** |  |  |
| negative regulation of protein ubiquitination |  |  | **6** | **0.019** | **6** | **0.025** |  |  | **7** | **0.018** | **16** | **<0.001** |  |  |
| negative regulation of ubiquitin-protein ligase activity |  |  | **6** | **0.016** | **6** | **0.021** |  |  | **7** | **0.015** | **16** | **<0.001** |  |  |
| negative regulation of ubiquitin-protein ligase activity during mitotic cell cycle |  |  | **6** | **0.015** | **6** | **0.020** |  |  | **7** | **0.014** | **16** | **<0.001** |  |  |
| positive regulation of ligase activity |  |  | **6** | **0.019** | **6** | **0.024** |  |  | **7** | **0.018** | **16** | **<0.001** |  |  |
| positive regulation of protein ubiquitination |  |  | **6** | **0.024** | **6** | **0.032** |  |  | **7** | **0.023** | **16** | **<0.001** |  |  |
| positive regulation of ubiquitin-protein ligase activity |  |  | **6** | **0.017** | **6** | **0.023** |  |  | **7** | **0.016** | **16** | **<0.001** |  |  |
| positive regulation of ubiquitin-protein ligase activity during mitotic cell cycle |  |  | **6** | **0.016** | **6** | **0.021** |  |  | **7** | **0.016** | **16** | **<0.001** |  |  |
| proteasomal protein catabolic process |  |  | **6** | **0.035** | **6** | **0.045** |  |  | **7** | **0.033** | **16** | **<0.001** |  |  |
| proteasomal ubiquitin-dependent protein catabolic process |  |  | **6** | **0.035** | **6** | **0.045** |  |  | **7** | **0.033** | **16** | **<0.001** |  |  |
| regulation of ligase activity |  |  | **6** | **0.023** | **6** | **0.030** |  |  | **7** | **0.022** | **16** | **<0.001** |  |  |
| regulation of protein ubiquitination |  |  | **6** | **0.034** | **6** | **0.044** |  |  | **7** | **0.032** | **16** | **<0.001** |  |  |
| regulation of ubiquitin-protein ligase activity |  |  | **6** | **0.021** | **6** | **0.028** |  |  | **7** | **0.020** | **16** | **<0.001** |  |  |
| regulation of ubiquitin-protein ligase activity during mitotic cell cycle |  |  | **6** | **0.018** | **6** | **0.023** |  |  | **7** | **0.017** | **16** | **<0.001** |  |  |
| positive regulation of cellular protein metabolic process |  |  | **9** | **0.029** | **8** | **0.042** |  |  |  |  | **16** | **<0.001** |  |  |
| positive regulation of protein metabolic process |  |  | **9** | **0.033** | **8** | **0.047** |  |  |  |  | **16** | **<0.001** |  |  |
| regulation of cellular protein metabolic process | 11 | 0.059 | **11** | **0.047** | **16** | **0.001** |  |  |  |  | **20** | **<0.001** |  |  |
| establishment of RNA localization |  |  | **6** | **0.032** | **6** | **0.009** |  |  | **7** | **0.001** |  |  | **9** | **0.003** |
| mRNA export from nucleus |  |  | **6** | **0.004** | **5** | **0.009** |  |  | **4** | **0.011** |  |  | **6** | **0.005** |
| nuclear export |  |  | **6** | **0.013** | **6** | **0.002** |  |  | **6** | **0.003** |  |  | **9** | **0.001** |
| nucleic acid transport |  |  | **6** | **0.032** | **6** | **0.009** |  |  | **7** | **0.001** |  |  | **9** | **0.003** |
| nucleobase. nucleoside. nucleotide and nucleic acid transport | 5 | 0.070 | **9** | **0.004** | **8** | **0.002** |  |  | **9** | **<0.001** |  |  | **11** | **<0.001** |
| RNA export from nucleus |  |  | **6** | **0.006** | **6** | **0.001** |  |  | **6** | **0.001** |  |  | **9** | **<0.001** |
| RNA localization |  |  | **6** | **0.034** | **6** | **0.010** |  |  | **7** | **0.001** |  |  | **9** | **0.004** |
| RNA transport |  |  | **6** | **0.032** | **6** | **0.009** |  |  | **7** | **0.001** |  |  | **9** | **0.003** |
| cellular protein localization | 9 | 0.073 | **11** | **0.030** |  |  | **17** | **0.025** | **13** | **0.001** | **10** | **0.050** |  |  |
| cellular macromolecule localization | 9 | 0.074 | **11** | **0.031** |  |  | **17** | **0.025** | **13** | **0.001** | 10 | 0.051 |  |  |
| protein localization |  |  | **15** | **0.039** |  |  | **22** | **0.044** | **14** | **0.022** |  |  |  |  |
| intracellular protein transport | 9 | 0.055 | **11** | **0.022** | 8 | 0.084 | 12 | 0.049 | **13** | **0.000** | **10** | **0.038** |  |  |
| negative regulation of catalytic activity |  |  | **9** | **0.045** |  |  |  |  | **9** | **0.042** | **16** | **0.000** |  |  |
| mRNA transport |  |  | **6** | **0.026** | 5 | 0.057 |  |  | **6** | **0.008** |  |  | **6** | **0.030** |
| establishment of protein localization |  |  | **15** | **0.022** |  |  |  |  | **14** | **0.010** |  |  | 13 | 0.088 |
| oxidative phosphorylation |  |  | **6** | **0.032** |  |  |  |  | **6** | **0.011** |  |  |  |  |
| protein import into nucleus. docking |  |  | **4** | **0.048** |  |  |  |  | **4** | **0.003** | 3 | 0.066 |  |  |
| protein transport |  |  | **15** | **0.021** |  |  |  |  | **14** | **0.009** |  |  | 13 | 0.085 |
| ubiquitin-dependent protein catabolic process |  |  | **9** | **0.032** |  |  |  |  |  |  | **16** | **0.000** |  |  |
| regulation of protein modification process |  |  | 9 | 0.053 | **12** | **0.003** |  |  | **9** | **0.049** | **18** | **0.000** |  |  |
| negative regulation of macromolecule metabolic process |  |  | 13 | 0.058 | **18** | **0.003** |  |  |  |  | **18** | **0.003** |  |  |
| positive regulation of protein modification process |  |  |  |  | **8** | **0.024** |  |  | 7 | 0.097 | **16** | **<0.001** |  |  |
| posttranscriptional regulation of gene expression |  |  | 11 | 0.062 | **8** | **0.033** |  |  |  |  |  |  | 8 | 0.093 |
| Golgi organization |  |  | 4 | 0.059 | **5** | **0.004** | **6** | **0.046** | 3 | 0.095 | 3 | 0.081 |  |  |
| nucleoside triphosphate metabolic process | 5 | 0.090 |  |  | **6** | **0.020** |  |  | **6** | **0.024** | **7** | **0.015** | **9** | **0.008** |
| Golgi vesicle transport |  |  |  |  | **6** | **0.020** |  |  | **6** | **0.024** |  |  |  |  |
| nucleobase. nucleoside and nucleotide biosynthetic process |  |  |  |  | **8** | **0.011** |  |  |  |  | **7** | **0.042** | **9** | **0.022** |
| nucleobase. nucleoside. nucleotide and nucleic acid biosynthetic process |  |  |  |  | **8** | **0.011** |  |  |  |  | **7** | **0.042** | **9** | **0.022** |
| nucleoside triphosphate biosynthetic process | 5 | 0.059 |  |  | **6** | **0.010** |  |  | 4 | 0.084 | **7** | **0.008** | **6** | **0.040** |
| nucleotide biosynthetic process |  |  |  |  | **8** | **0.009** |  |  |  |  | **7** | **0.038** | **9** | **0.020** |
| nucleoside metabolic process |  |  |  |  | **5** | **0.033** |  |  |  |  |  |  |  |  |
| activation of caspase activity |  |  |  |  |  |  | **13** | **0.003** |  |  |  |  |  |  |
| activation of caspase activity by cytochrome c |  |  |  |  |  |  | **9** | **0.014** |  |  |  |  |  |  |
| mitochondrial membrane organization |  |  |  |  |  |  | **9** | **0.049** |  |  |  |  |  |  |
| mitochondrion organization |  |  |  |  |  |  | **13** | **0.019** |  |  |  |  |  |  |
| positive regulation of caspase activity |  |  |  |  |  |  | **13** | **0.004** |  |  |  |  |  |  |
| positive regulation of hydrolase activity |  |  |  |  |  |  | **13** | **0.031** |  |  |  |  |  |  |
| positive regulation of peptidase activity |  |  |  |  |  |  | **13** | **0.004** |  |  |  |  |  |  |
| regulation of caspase activity |  |  |  |  |  |  | **13** | **0.007** |  |  |  |  |  |  |
| regulation of endopeptidase activity |  |  |  |  |  |  | **13** | **0.007** |  |  |  |  |  |  |
| regulation of peptidase activity |  |  |  |  |  |  | **13** | **0.008** |  |  |  |  |  |  |
| response to hypoxia |  |  |  |  |  |  | **9** | **0.035** |  |  | 5 | 0.100 | 6 | 0.065 |
| response to oxygen levels |  |  |  |  |  |  | **9** | **0.039** |  |  |  |  | 6 | 0.071 |
| vesicle-mediated transport |  |  | 11 | 0.083 |  |  | **15** | **0.037** | 10 | 0.054 |  |  |  |  |
| macromolecular complex assembly |  |  |  |  |  |  |  |  | **13** | **0.013** | **11** | **0.048** |  |  |
| nuclear import |  |  |  |  |  |  |  |  | **6** | **0.008** | **5** | **0.048** |  |  |
| protein import into nucleus |  |  |  |  |  |  |  |  | **6** | **0.008** | **5** | **0.046** |  |  |
| protein oligomerization |  |  | 6 | 0.089 |  |  |  |  | **6** | **0.049** | **7** | **0.032** |  |  |
| cellular protein complex assembly |  |  |  |  |  |  |  |  | **6** | **0.041** |  |  |  |  |
| cellular respiration |  |  |  |  |  |  |  |  | **6** | **0.011** |  |  |  |  |
| COPI coating of Golgi vesicle |  |  |  |  |  |  |  |  | **3** | **0.046** |  |  |  |  |
| energy derivation by oxidation of organic compounds |  |  |  |  |  |  |  |  | **7** | **0.005** |  |  |  |  |
| Golgi transport vesicle coating |  |  |  |  |  |  |  |  | **3** | **0.046** |  |  |  |  |
| Golgi vesicle budding |  |  |  |  |  |  |  |  | **3** | **0.046** |  |  |  |  |
| macromolecular complex subunit organization |  |  |  |  |  |  |  |  | **13** | **0.018** | 11 | 0.063 |  |  |
| protein import |  |  |  |  |  |  |  |  | **6** | **0.024** | 5 | 0.096 |  |  |
| protein localization in nucleus |  |  |  |  |  |  |  |  | **6** | **0.010** | 5 | 0.054 |  |  |
| protein localization in organelle |  |  |  |  |  |  |  |  | **6** | **0.032** |  |  |  |  |
| protein targeting |  |  |  |  |  |  |  |  | **7** | **0.019** |  |  |  |  |
| respiratory electron transport chain |  |  |  |  |  |  |  |  | **4** | **0.037** |  |  |  |  |
| mRNA stabilization |  |  |  |  |  |  |  |  |  |  | **4** | **0.050** | **6** | **0.036** |
| RNA stabilization |  |  |  |  |  |  |  |  |  |  | **4** | **0.050** | **6** | **0.036** |
| rRNA metabolic process |  |  |  |  |  |  |  |  |  |  | **6** | **0.042** | **8** | **0.023** |
| rRNA processing |  |  |  |  |  |  |  |  |  |  | **6** | **0.039** | **8** | **0.021** |
| anti-apoptosis |  |  |  |  |  |  |  |  |  |  | **8** | **0.033** |  |  |
| apoptosis |  |  |  |  |  |  |  |  |  |  | **16** | **0.004** |  |  |
| axon guidance |  |  |  |  |  |  |  |  |  |  | **8** | **0.006** |  |  |
| axonogenesis |  |  |  |  |  |  |  |  |  |  | **10** | **0.004** |  |  |
| cell cycle |  |  |  |  |  |  |  |  |  |  | **22** | **0.000** | 13 | 0.091 |
| cell cycle process |  |  |  |  |  |  |  |  |  |  | **20** | **0.000** |  |  |
| cell death |  |  |  |  |  |  |  |  |  |  | **16** | **0.010** |  |  |
| cell morphogenesis |  |  |  |  |  |  |  |  |  |  | **10** | **0.032** |  |  |
| cell morphogenesis involved in differentiation |  |  |  |  |  |  |  |  |  |  | **10** | **0.009** |  |  |
| cell morphogenesis involved in neuron differentiation |  |  |  |  |  |  |  |  |  |  | **10** | **0.005** |  |  |
| cell motion |  |  |  |  |  |  |  |  |  |  | **12** | **0.022** |  |  |
| cell part morphogenesis |  |  |  |  |  |  |  |  |  |  | **10** | **0.011** |  |  |
| cell projection morphogenesis |  |  |  |  |  |  |  |  |  |  | **10** | **0.009** |  |  |
| cell projection organization |  |  |  |  |  |  |  |  |  |  | **12** | **0.008** |  |  |
| cellular component morphogenesis |  |  |  |  |  |  |  |  |  |  | **12** | **0.011** |  |  |
| cellular macromolecule catabolic process |  |  |  |  |  |  |  |  |  |  | **16** | **0.011** |  |  |
| cellular protein catabolic process |  |  |  |  |  |  |  |  |  |  | **16** | **0.004** |  |  |
| death |  |  |  |  |  |  |  |  |  |  | **16** | **0.011** |  |  |
| ER-associated protein catabolic process |  |  |  |  |  |  |  |  |  |  | **8** | **0.000** |  |  |
| induction of apoptosis |  |  |  |  |  |  | 13 | 0.087 |  |  | **12** | **0.004** |  |  |
| induction of apoptosis by extracellular signals |  |  |  |  |  |  |  |  |  |  | **8** | **0.007** |  |  |
| induction of programmed cell death |  |  |  |  |  |  | 13 | 0.088 |  |  | **12** | **0.005** |  |  |
| long-term strengthening of neuromuscular junction |  |  |  |  |  |  |  |  |  |  | **8** | **0.000** |  |  |
| macromolecule catabolic process |  |  |  |  |  |  |  |  |  |  | **16** | **0.016** |  |  |
| mitotic cell cycle |  |  |  |  | 8 | 0.081 |  |  |  |  | **20** | **0.000** |  |  |
| modification-dependent macromolecule catabolic process |  |  |  |  |  |  |  |  |  |  | **16** | **0.003** |  |  |
| modification-dependent protein catabolic process |  |  |  |  |  |  |  |  |  |  | **16** | **0.003** |  |  |
| ncRNA metabolic process |  |  |  |  |  |  | 9 | 0.092 |  |  | **8** | **0.043** |  |  |
| negative regulation of molecular function |  |  | 9 | 0.071 | 8 | 0.099 |  |  | 9 | 0.067 | **16** | **0.000** |  |  |
| neuron development |  |  |  |  |  |  |  |  |  |  | **12** | **0.006** |  |  |
| neuron differentiation |  |  |  |  |  |  |  |  |  |  | **12** | **0.016** |  |  |
| neuron projection development |  |  |  |  |  |  |  |  |  |  | **12** | **0.002** |  |  |
| neuron projection morphogenesis |  |  |  |  |  |  |  |  |  |  | **10** | **0.006** |  |  |
| positive regulation of apoptosis |  |  |  |  |  |  |  |  |  |  | **16** | **0.001** |  |  |
| positive regulation of catalytic activity |  |  |  |  | 10 | 0.094 |  |  |  |  | **18** | **0.000** |  |  |
| positive regulation of cell communication |  |  |  |  |  |  |  |  |  |  | **14** | **0.001** |  |  |
| positive regulation of cell death |  |  |  |  |  |  |  |  |  |  | **16** | **0.001** |  |  |
| positive regulation of macromolecule metabolic process |  |  | 13 | 0.098 |  |  |  |  |  |  | **18** | **0.008** |  |  |
| positive regulation of molecular function |  |  |  |  |  |  |  |  |  |  | **18** | **0.001** |  |  |
| positive regulation of multicellular organismal process |  |  |  |  |  |  |  |  |  |  | **8** | **0.050** |  |  |
| positive regulation of neurological system process |  |  |  |  |  |  |  |  |  |  | **8** | **0.000** |  |  |
| positive regulation of programmed cell death |  |  |  |  |  |  |  |  |  |  | **16** | **0.001** |  |  |
| positive regulation of synaptic transmission |  |  |  |  |  |  |  |  |  |  | **8** | **0.000** |  |  |
| positive regulation of transmission of nerve impulse |  |  |  |  |  |  |  |  |  |  | **8** | **0.000** |  |  |
| programmed cell death |  |  |  |  |  |  |  |  |  |  | **16** | **0.004** |  |  |
| protein catabolic process |  |  |  |  |  |  |  |  |  |  | **16** | **0.005** |  |  |
| protein modification by small protein conjugation |  |  |  |  |  |  |  |  |  |  | **8** | **0.010** |  |  |
| protein modification by small protein conjugation or removal |  |  |  |  |  |  |  |  |  |  | **8** | **0.017** |  |  |
| protein ubiquitination |  |  |  |  |  |  |  |  |  |  | **8** | **0.008** |  |  |
| proteolysis involved in cellular protein catabolic process |  |  |  |  |  |  |  |  |  |  | **16** | **0.004** |  |  |
| regulation of apoptosis |  |  |  |  |  |  |  |  |  |  | **18** | **0.005** |  |  |
| regulation of cell death |  |  |  |  |  |  |  |  |  |  | **18** | **0.006** |  |  |
| regulation of cell development |  |  |  |  |  |  |  |  |  |  | **8** | **0.032** |  |  |
| regulation of cellular component biogenesis |  |  |  |  |  |  |  |  |  |  | **8** | **0.012** |  |  |
| regulation of developmental growth |  |  |  |  |  |  |  |  |  |  | **8** | **0.001** |  |  |
| regulation of growth |  |  |  |  |  |  |  |  |  |  | **10** | **0.028** |  |  |
| regulation of muscle cell differentiation |  |  |  |  |  |  |  |  |  |  | **8** | **0.000** |  |  |
| regulation of muscle development |  |  |  |  |  |  |  |  |  |  | **8** | **0.001** |  |  |
| regulation of nervous system development |  |  |  |  |  |  |  |  |  |  | **8** | **0.027** |  |  |
| regulation of neurological system process |  |  |  |  |  |  |  |  |  |  | **8** | **0.015** |  |  |
| regulation of programmed cell death |  |  |  |  |  |  |  |  |  |  | **18** | **0.006** |  |  |
| regulation of skeletal muscle fiber development |  |  |  |  |  |  |  |  |  |  | **8** | **0.000** |  |  |
| regulation of skeletal muscle tissue development |  |  |  |  |  |  |  |  |  |  | **8** | **0.000** |  |  |
| regulation of striated muscle cell differentiation |  |  |  |  |  |  |  |  |  |  | **8** | **0.000** |  |  |
| regulation of striated muscle tissue development |  |  |  |  |  |  |  |  |  |  | **8** | **0.001** |  |  |
| regulation of synapse organization |  |  |  |  |  |  |  |  |  |  | **8** | **0.000** |  |  |
| regulation of synapse structure and activity |  |  |  |  |  |  |  |  |  |  | **8** | **0.000** |  |  |
| regulation of synaptic growth at neuromuscular junction |  |  |  |  |  |  |  |  |  |  | **8** | **0.000** |  |  |
| regulation of synaptic plasticity |  |  |  |  |  |  |  |  |  |  | **8** | **0.001** |  |  |
| regulation of synaptic transmission |  |  |  |  |  |  |  |  |  |  | **8** | **0.011** |  |  |
| regulation of synaptogenesis |  |  |  |  |  |  |  |  |  |  | **8** | **0.000** |  |  |
| regulation of transmission of nerve impulse |  |  |  |  |  |  |  |  |  |  | **8** | **0.014** |  |  |
| synaptic transmission |  |  |  |  |  |  |  |  |  |  | **10** | **0.018** |  |  |
| transmission of nerve impulse |  |  |  |  |  |  |  |  |  |  | **10** | **0.031** |  |  |
| response to temperature stimulus |  |  |  |  |  |  |  |  |  |  | **5** | **0.043** |  |  |
| antigen processing and presentation of exogenous antigen |  |  |  |  | 3 | 0.060 |  |  |  |  |  |  | **4** | **0.043** |
| ATP metabolic process |  |  |  |  |  |  |  |  |  |  |  |  | **6** | **0.042** |
| coenzyme biosynthetic process |  |  |  |  |  |  |  |  |  |  |  |  | **6** | **0.019** |
| cofactor biosynthetic process |  |  |  |  |  |  |  |  |  |  |  |  | **6** | **0.036** |
| positive regulation of cell activation |  |  |  |  | 5 | 0.087 |  |  |  |  |  |  | **6** | **0.046** |
| positive regulation of leukocyte activation |  |  |  |  | 5 | 0.080 |  |  |  |  |  |  | **6** | **0.043** |
| positive regulation of lymphocyte activation |  |  |  |  | 5 | 0.069 |  |  |  |  |  |  | **6** | **0.036** |
| purine nucleoside triphosphate biosynthetic process |  |  |  |  | 5 | 0.071 |  |  | 4 | 0.080 | 5 | 0.059 | **6** | **0.038** |
| purine nucleoside triphosphate metabolic process |  |  |  |  |  |  |  |  |  |  | 5 | 0.085 | **9** | **0.006** |
| purine nucleotide metabolic process |  |  |  |  |  |  |  |  |  |  |  |  | **9** | **0.020** |
| purine ribonucleoside triphosphate biosynthetic process |  |  |  |  | 5 | 0.070 |  |  | 4 | 0.078 | 5 | 0.058 | **6** | **0.037** |
| purine ribonucleoside triphosphate metabolic process |  |  |  |  | 5 | 0.095 |  |  |  |  | 5 | 0.079 | **9** | **0.006** |
| purine ribonucleotide metabolic process |  |  |  |  |  |  |  |  |  |  |  |  | **9** | **0.009** |
| ribonucleoside triphosphate biosynthetic process |  |  |  |  | 5 | 0.071 |  |  | 4 | 0.080 | 5 | 0.059 | **6** | **0.038** |
| ribonucleoside triphosphate metabolic process |  |  |  |  | 5 | 0.096 |  |  |  |  | 5 | 0.081 | **9** | **0.006** |
| ribonucleotide metabolic process |  |  |  |  |  |  |  |  |  |  |  |  | **9** | **0.011** |
| actin cytoskeleton organization | 7 | 0.056 |  |  |  |  |  |  |  |  |  |  |  |  |
| actin filament-based process | 7 | 0.065 |  |  |  |  |  |  |  |  |  |  |  |  |
| amino acid activation |  |  |  |  |  |  | 6 | 0.097 |  |  |  |  |  |  |
| carbohydrate catabolic process | 5 | 0.066 |  |  |  |  |  |  | 4 | 0.094 |  |  |  |  |
| cell proliferation |  |  |  |  |  |  |  |  |  |  | 8 | 0.096 |  |  |
| cellular aldehyde metabolic process | 4 | 0.083 |  |  |  |  |  |  |  |  |  |  |  |  |
| cellular homeostasis |  |  |  |  | 9 | 0.055 |  |  |  |  |  |  |  |  |
| cholesterol biosynthetic process | 7 | 0.190 |  |  |  |  |  |  |  |  |  |  |  |  |
| coenzyme metabolic process |  |  |  |  |  |  |  |  |  |  |  |  | 6 | 0.082 |
| eating behavior | 3 | 0.085 | 4 | 0.064 | 3 | 0.097 |  |  |  |  | 3 | 0.088 |  |  |
| fructose metabolic process | 3 | 0.063 |  |  |  |  |  |  | 3 | 0.077 |  |  |  |  |
| glutamine family amino acid biosynthetic process | 3 | 0.071 |  |  | 3 | 0.081 |  |  |  |  | 3 | 0.073 |  |  |
| induction of apoptosis by intracellular signals |  |  |  |  |  |  | 9 | 0.081 |  |  |  |  |  |  |
| membrane budding |  |  |  |  |  |  |  |  | 3 | 0.073 |  |  |  |  |
| monosaccharide metabolic process | 7 | 0.054 |  |  |  |  | 9 | 0.086 | 6 | 0.088 |  |  |  |  |
| ncRNA processing |  |  |  |  |  |  |  |  |  |  |  |  | 8 | 0.076 |
| negative regulation of protein amino acid phosphorylation | 4 | 0.095 |  |  |  |  |  |  |  |  |  |  |  |  |
| nucleoside diphosphate metabolic process | 3 | 0.063 |  |  | 3 | 0.073 |  |  |  |  | 3 | 0.066 |  |  |
| nucleotide-excision repair. DNA damage removal |  |  |  |  |  |  |  |  |  |  | 3 | 0.084 |  |  |
| positive regulation of alpha-beta T cell activation |  |  |  |  |  |  |  |  |  |  |  |  | 4 | 0.078 |
| positive regulation of alpha-beta T cell differentiation |  |  |  |  | 3 | 0.085 |  |  |  |  |  |  | 4 | 0.060 |
| positive regulation of calcium ion transport |  |  | 7 | 0.062 |  |  |  |  |  |  |  |  |  |  |
| positive regulation of ion transport |  |  | 7 | 0.078 |  |  |  |  |  |  |  |  |  |  |
| positive regulation of T cell differentiation |  |  |  |  |  |  |  |  |  |  |  |  | 4 | 0.095 |
| protein export from nucleus |  |  |  |  | 3 | 0.077 |  |  | 3 | 0.082 |  |  | 4 | 0.054 |
| protein homooligomerization |  |  |  |  | 5 | 0.066 |  |  | 4 | 0.074 |  |  |  |  |
| protein ubiquitination during ubiquitin-dependent protein catabolic process |  |  |  |  |  |  |  |  |  |  | 3 | 0.066 |  |  |
| proteolysis |  |  |  |  |  |  |  |  |  |  | 16 | 0.064 |  |  |
| purine nucleotide biosynthetic process |  |  |  |  |  |  |  |  |  |  |  |  | 6 | 0.077 |
| purine ribonucleotide biosynthetic process |  |  |  |  | 5 | 0.095 |  |  |  |  | 5 | 0.079 | 6 | 0.051 |
| pyrimidine nucleoside metabolic process | 3 | 0.092 |  |  |  |  |  |  |  |  | 3 | 0.095 |  |  |
| pyrimidine nucleoside triphosphate metabolic process | 3 | 0.053 |  |  | 3 | 0.060 |  |  | 3 | 0.064 | 3 | 0.054 |  |  |
| pyrimidine nucleotide biosynthetic process | 3 | 0.081 |  |  | 3 | 0.093 |  |  |  |  | 3 | 0.084 |  |  |
| regulation of alpha-beta T cell differentiation |  |  |  |  |  |  |  |  |  |  |  |  | 4 | 0.075 |
| regulation of hydrolase activity |  |  |  |  |  |  | 13 | 0.095 |  |  |  |  |  |  |
| regulation of leukocyte activation |  |  |  |  |  |  |  |  |  |  |  |  | 6 | 0.094 |
| regulation of lymphocyte activation |  |  |  |  |  |  |  |  |  |  |  |  | 6 | 0.077 |
| regulation of mRNA stability |  |  |  |  |  |  |  |  |  |  | 4 | 0.072 | 6 | 0.052 |
| regulation of RNA stability |  |  |  |  |  |  |  |  |  |  | 4 | 0.079 | 6 | 0.057 |
| regulation of system process |  |  |  |  |  |  |  |  |  |  | 8 | 0.087 |  |  |
| response to cold |  |  |  |  | 4 | 0.068 |  |  |  |  |  |  |  |  |
| response to organic substance |  |  |  |  |  |  |  |  |  |  | 11 | 0.066 |  |  |
| retrograde vesicle-mediated transport. Golgi to ER |  |  |  |  |  |  |  |  | 3 | 0.095 |  |  |  |  |
| ribonucleotide biosynthetic process |  |  |  |  |  |  |  |  |  |  | 5 | 0.088 | 6 | 0.057 |
| steroid metabolic process |  |  |  |  |  |  |  |  | 6 | 0.071 |  |  |  |  |
| transmembrane transport |  |  |  |  |  |  |  |  |  |  |  |  | 11 | 0.099 |
| tRNA aminoacylation |  |  |  |  |  |  | 6 | 0.097 |  |  |  |  |  |  |
| tRNA aminoacylation for protein translation |  |  |  |  |  |  | 6 | 0.097 |  |  |  |  |  |  |
| tRNA metabolic process |  |  |  |  |  |  |  |  |  |  | 5 | 0.081 |  |  |
| vesicle coating |  |  |  |  |  |  |  |  | 3 | 0.069 |  |  |  |  |
| vesicle targeting |  |  |  |  |  |  |  |  | 3 | 0.099 |  |  |  |  |
| vesicle targeting. to. from or within Golgi |  |  |  |  |  |  |  |  | 3 | 0.055 |  |  |  |  |

**Table S3**

| **Gene ontology analysis** | **TSA**  **Normoxia** | | **NAM**  **Normoxia** | | **TSA + NAM Normoxia** | | **Hypoxia** | | **TSA**  **Hypoxia** | | **NAM**  **Hypoxia** | | **TSA + NAM Hypoxia** | |
| --- | --- | --- | --- | --- | --- | --- | --- | --- | --- | --- | --- | --- | --- | --- |
| **Protein Information Resource (PIR) Keywords** | **Percentage of proteins** | **p-value** | **Percentage of proteins** | **p-value** | **Percentage of proteins** | **p-value** | **Percentage of proteins** | **p-value** | **Percentage of proteins** | **p-value** | **Percentage of proteins** | **p-value** | **Percentage of proteins** | **p-value** |
| Acetylation | **70** | **<0.001** | **76** | **<0.001** | **63** | **<0.001** | **61** | **<0.001** | **68** | **<0.001** | **66** | **<0.001** | **64** | **<0.001** |
| Ribosome | **12** | **<0.001** | **4** | **0.031** | **1** | **<0.001** | **5** | **0.019** | **8** | **<0.001** | **14** | **<0.001** | **10** | **<0.001** |
| Protein Biosynthesis | **16** | **<0.001** | **15** | **<0.001** | **17** | **<0.001** | **14** | **<0.001** | **11** | **<0.001** | **19** | **<0.001** | **16** | **<0.001** |
| Ribonucleoprotein | **16** | **<0.001** |  |  | **16** | **<0.001** | **14** | **<0.001** | **11** | **<0.001** | **20** | **<0.001** | **12** | **<0.001** |
| Ribosomal protein | **13** | **<0.001** | **7** | **0.005** | **12** | **<0.001** | **11** | **<0.001** | **10** | **<0.001** | **16** | **<0.001** | **10** | **<0.001** |
| Phosphoprotein | **74** | **<0.001** | **61** | **<0.001** | **65** | **<0.001** | **68** | **<0.001** | **68** | **<0.001** | **64** | **<0.001** | **72** | **<0.001** |
| Cytoplasm | **42** | **<0.001** | **34** | **<0.001** | **35** | **<0.001** | **43** | **<0.001** | **37** | **<0.001** | **4** | **<0.001** | **4** | **<0.001** |
| RNA-binding | **13** | **<0.001** | **12** | **<0.001** | **13** | **<0.001** | **13** | **0.004** | **9** | **0.005** | **12** | **<0.001** | **15** | **<0.001** |
| mRNA splicing | **9** | **<0.001** | **10** | **<0.001** | **8** | **<0.001** | **7** | **0.020** | **8** | **<0.001** | **7** | **<0.001** | **10** | **<0.001** |
| mRNA procesing | **9** | **<0.001** | **10** | **<0.001** |  |  | **5** | **0.019** | **8** | **<0.001** | **7** | **<0.001** | **10** | **<0.001** |
| Methylation | **8** | **<0.001** | **8** | **0.002** | **6** | **0.003** |  |  |  |  | **7** | **<0.001** | **6** | **0.020** |
| Blocked amino end | **5** | **0.002** | **5** | **0.005** | **6** | **<0.001** | **5** | **0.029** | **4** | **0.002** | **4** | **0.002** | **6** | **<0.001** |
| NADP | **6** | **0.002** |  |  |  |  |  |  |  |  |  |  |  |  |
| Glycolysis | **4** | **0.002** |  |  |  |  |  |  | **3** | **0.028** |  |  | **4** | **0.015** |
| Actin-binding | **7** | **0.004** |  |  |  |  |  |  | **7** | **<0.001** |  |  |  |  |
| Oxidoreductase | **9** | **0.004** | 8 | 0.062 |  |  |  |  |  |  | **7** | **0.046** |  |  |
| Spliceosome | **5** | **0.006** | 4 | 0.076 | **6** | **<0.001** | **5** | **0.046** |  |  | **4** | **0.005** | **7** | **<0.001** |
| Mitochondrion | **11** | **0.008** | 10 | 0.096 | **11** | **0.004** | **13** | **0.031** | **17** | **<0.001** | **10** | **0.025** | **15** | **<0.001** |
