# Supplementary figures and images for: Quantitative Proteomic Approach Reveals Altered Metabolic Pathways in Response to the Inhibition of Lysine Deacetylases in A549 Cells under Normoxia and Hypoxia
Source: Int J Mol Sci. 2021 Mar 25;22(7):3378. doi: 10.3390/ijms22073378 (PMC8036653; doi:10.3390/ijms22073378)

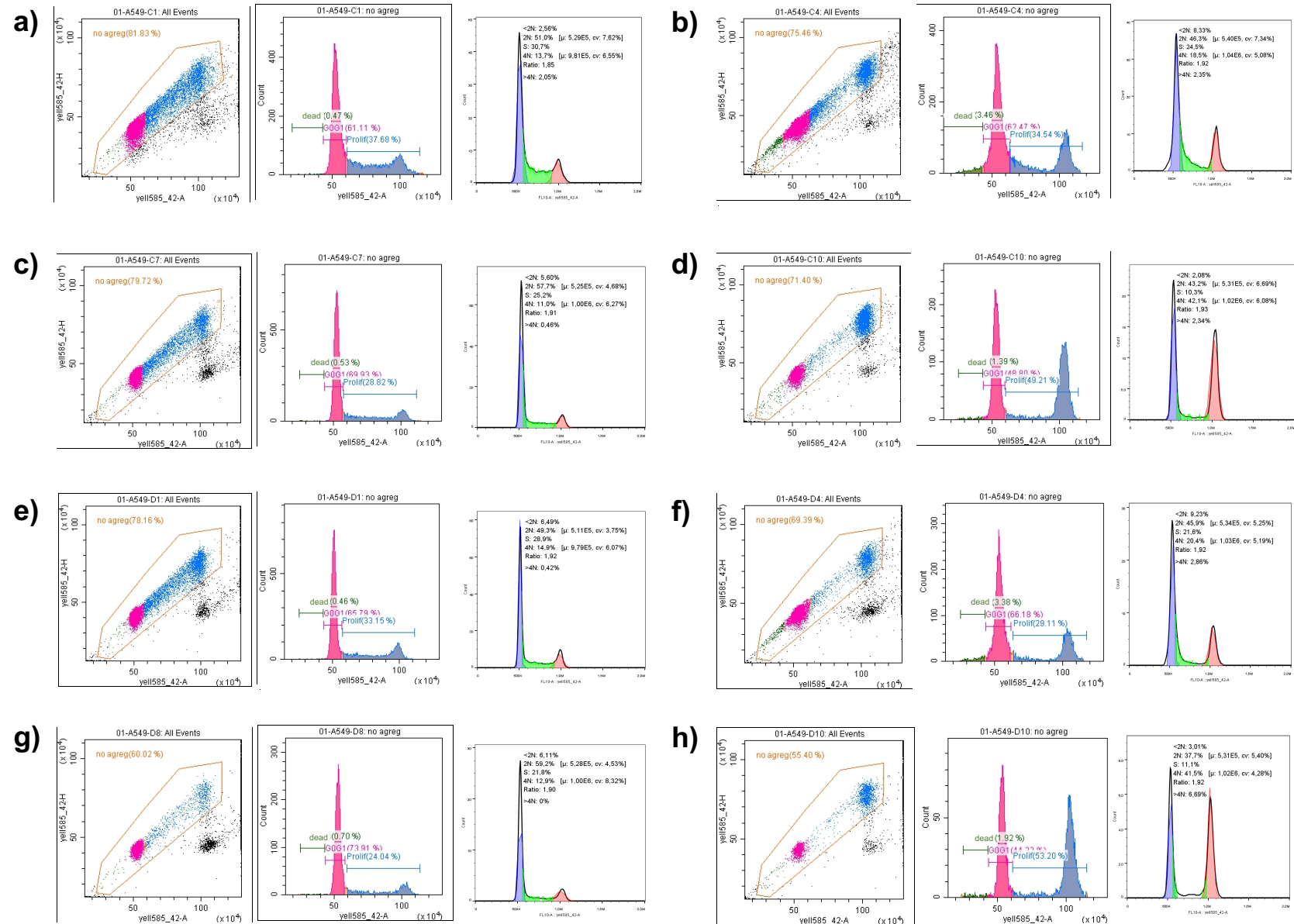

Supplement: Supplementary file 1 [file ijms-22-03378-s001.zip › 20210323 Supplementary Files IJMS/FigureS4-new.pdf]

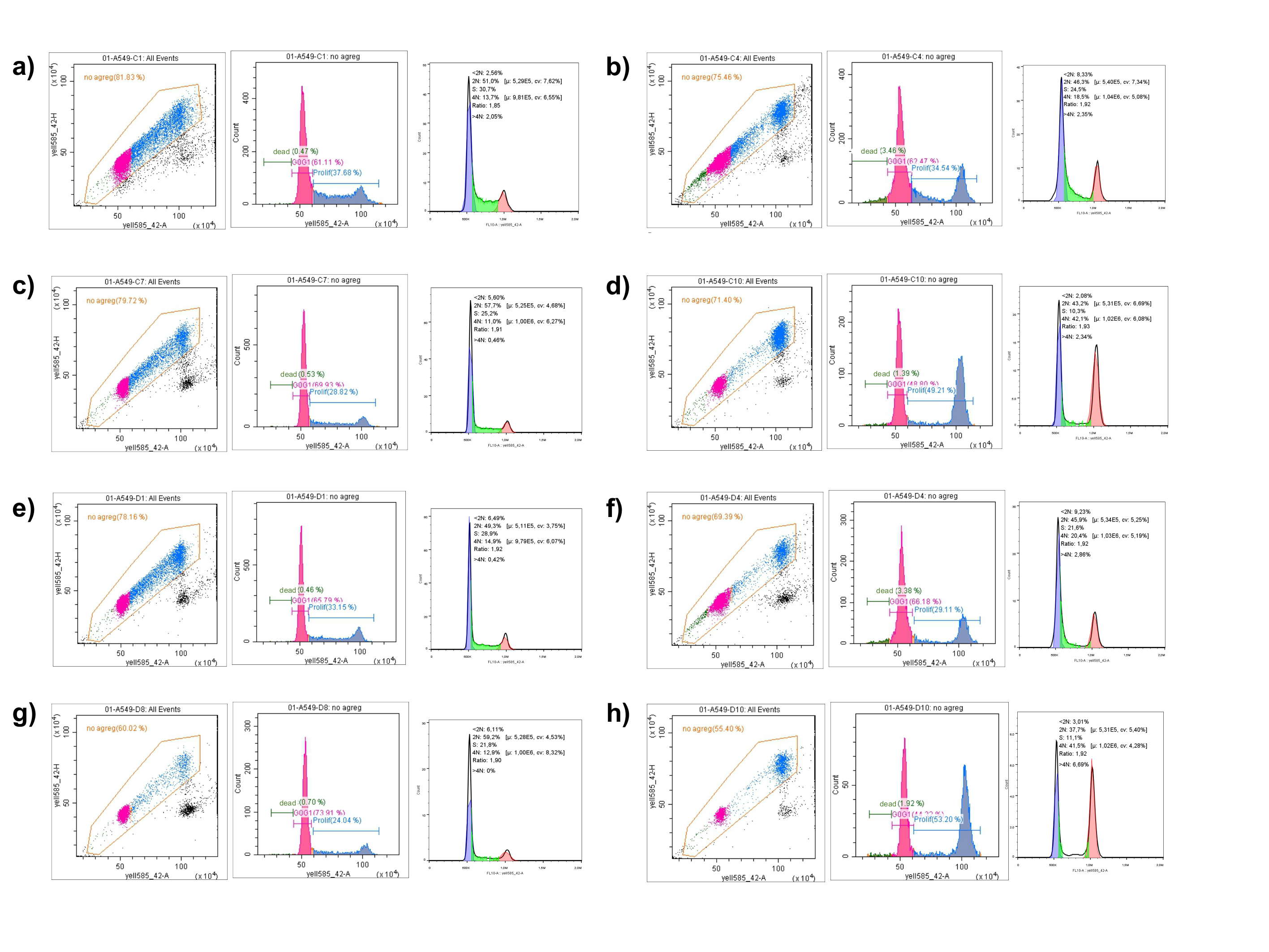

Supplement: Supplementary file 1 [file ijms-22-03378-s001.zip › 20210323 Supplementary Files IJMS/FigureS4-new.tif]

**A)**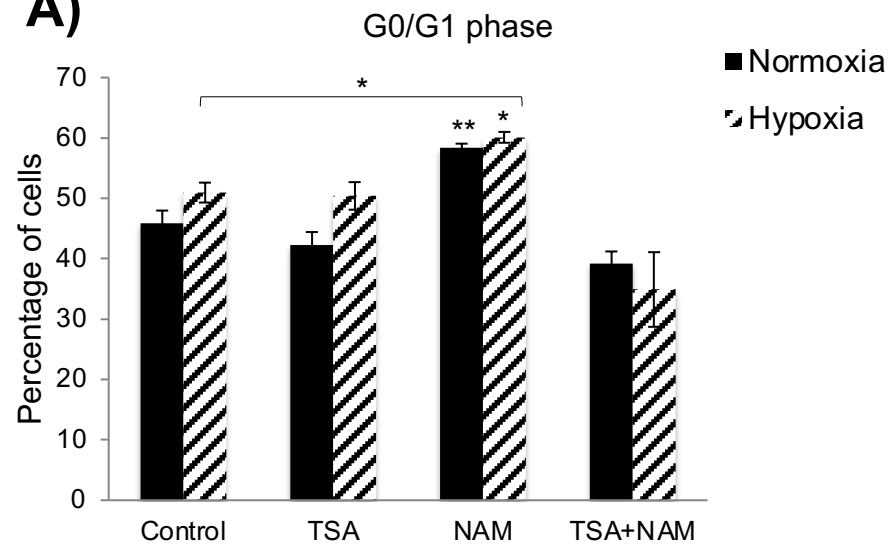**B)**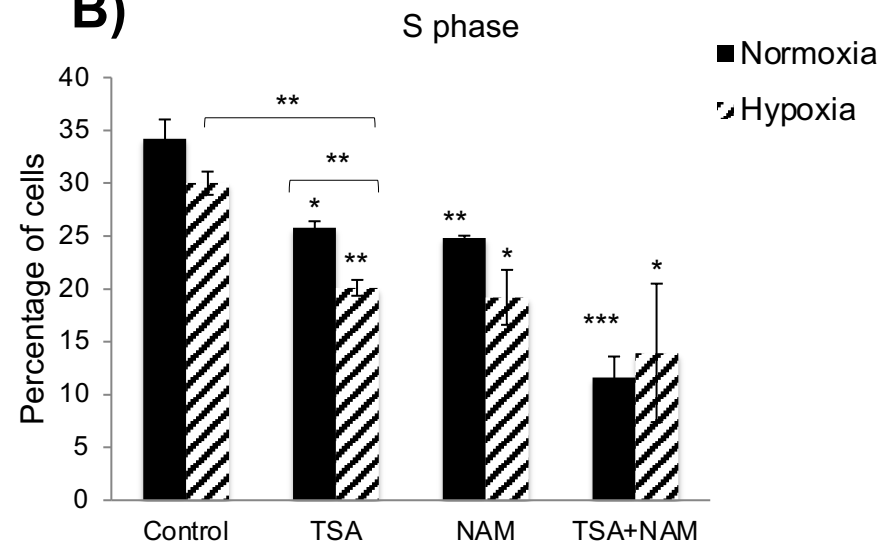**C)**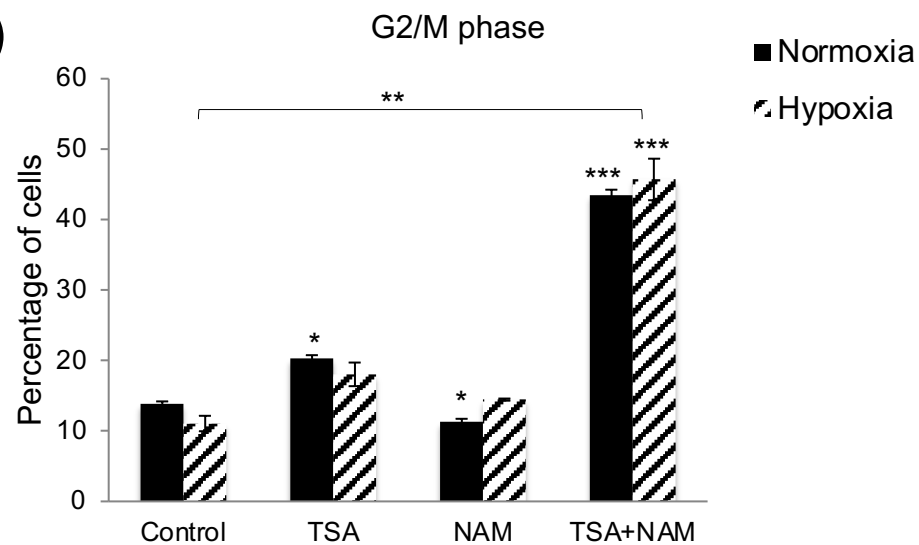

Supplement: Supplementary file 1 [file ijms-22-03378-s001.zip › 20210323 Supplementary Files IJMS/FigureS2-new.pdf]

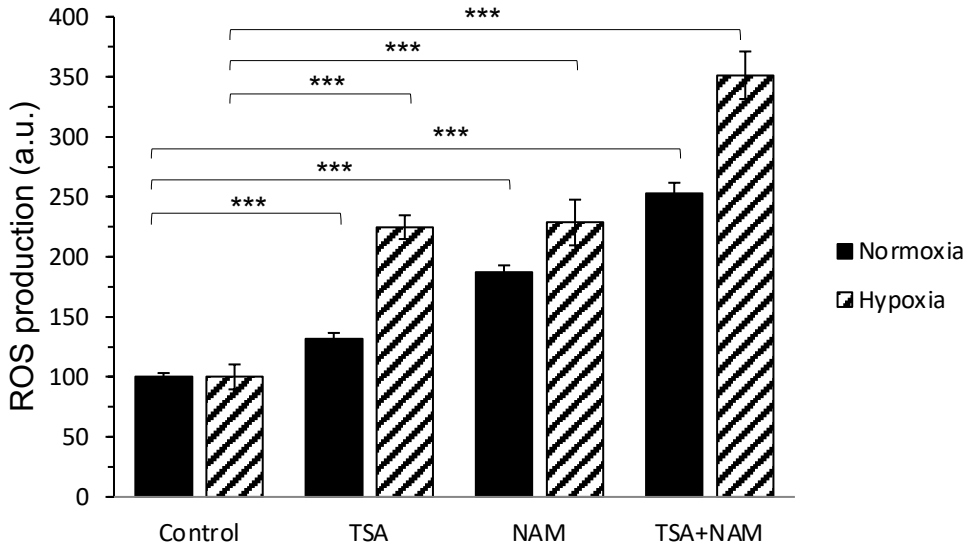

Supplement: Supplementary file 1 [file ijms-22-03378-s001.zip › 20210323 Supplementary Files IJMS/FigureS3-new.pdf]

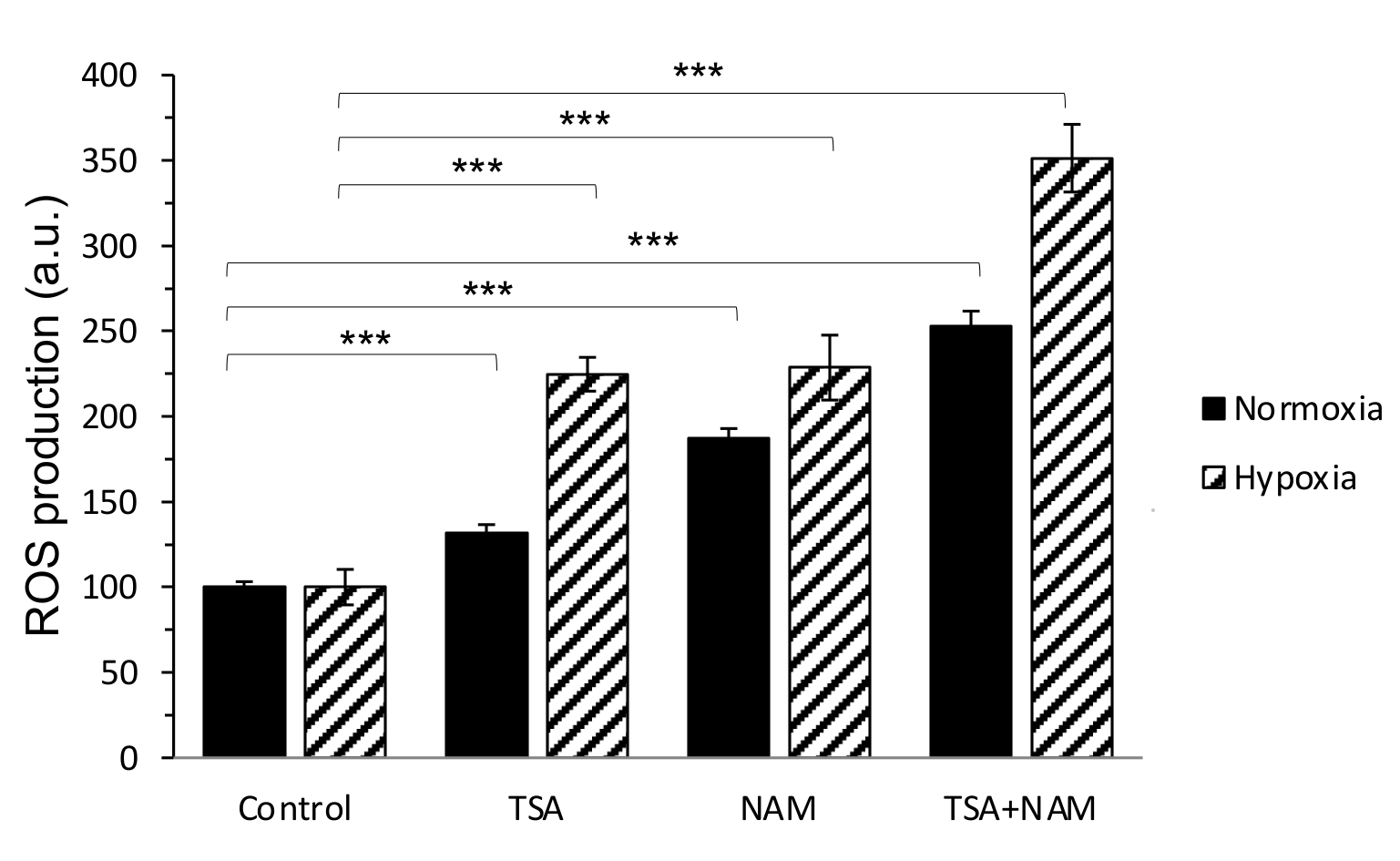

Supplement: Supplementary file 1 [file ijms-22-03378-s001.zip › 20210323 Supplementary Files IJMS/FigureS3-new.tif]

**A**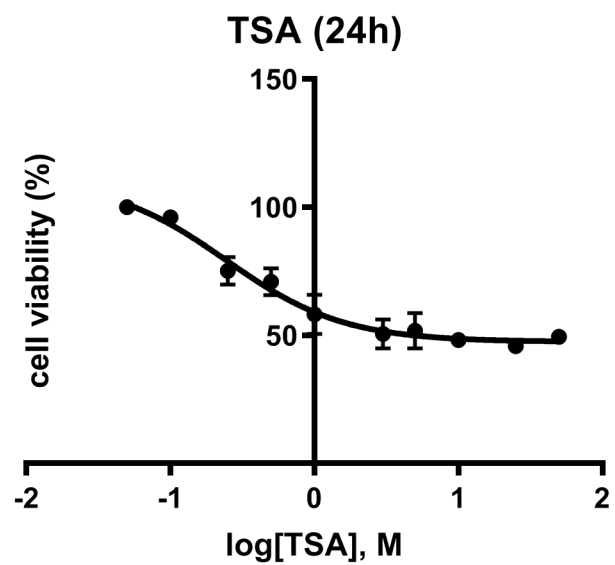**B**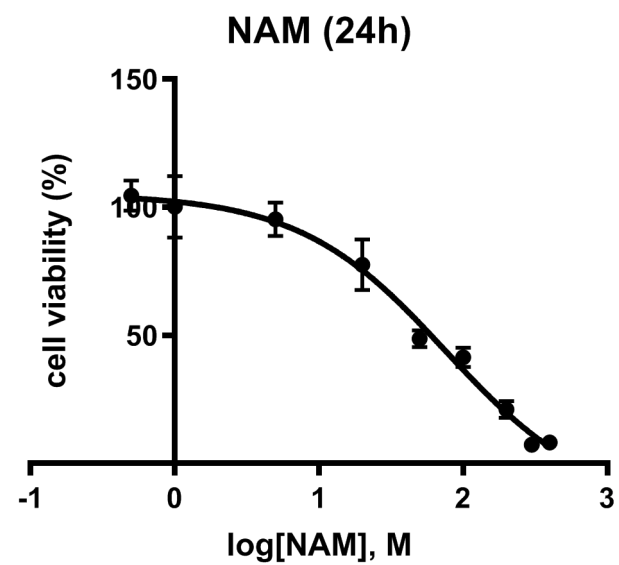

Supplement: Supplementary file 1 [file ijms-22-03378-s001.zip › 20210323 Supplementary Files IJMS/FigureS1.pdf]

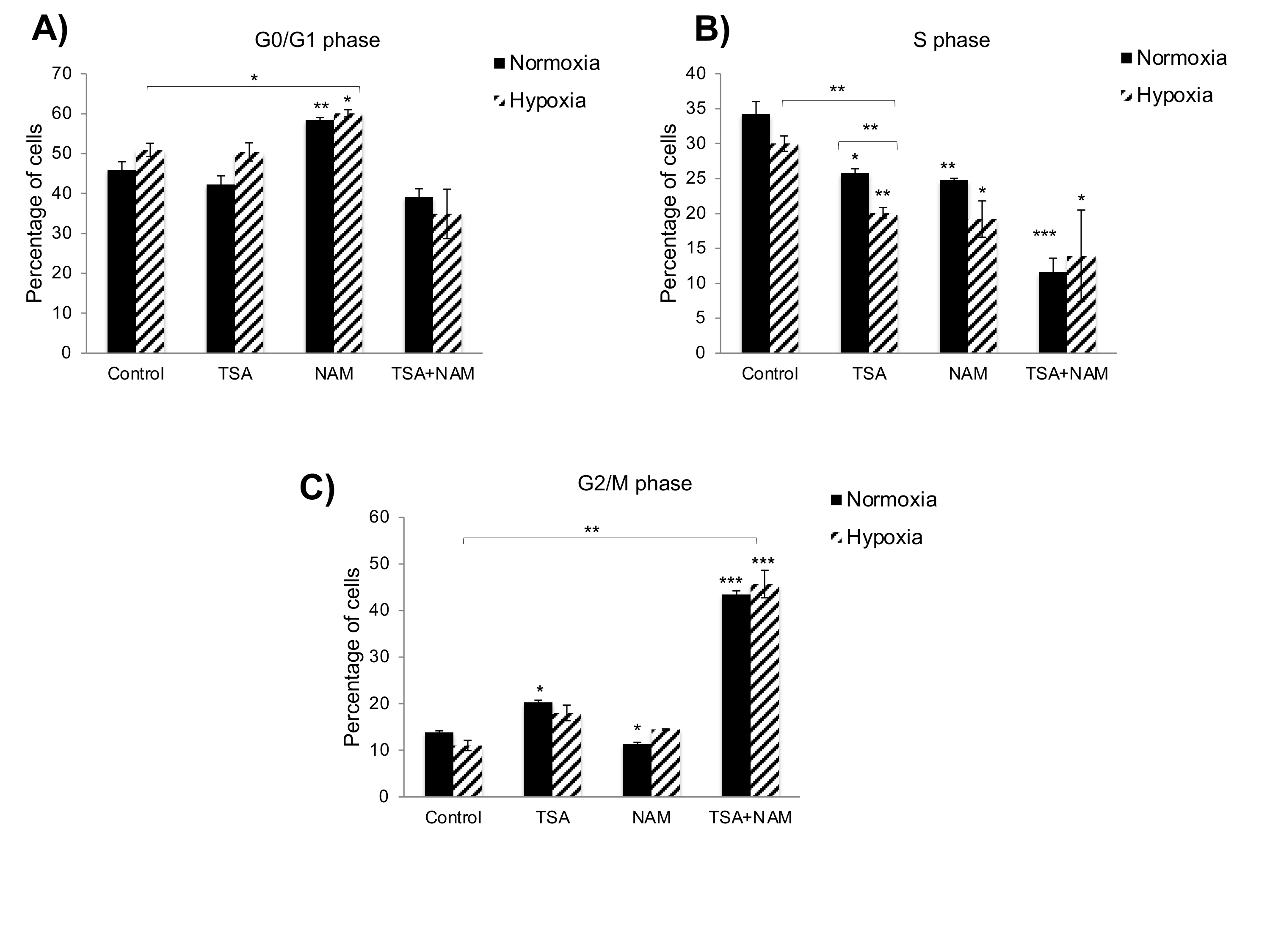

Supplement: Supplementary file 1 [file ijms-22-03378-s001.zip › 20210323 Supplementary Files IJMS/FigureS2-new.tif]
